# Cancer cachexia: molecular basis and therapeutic advances

**DOI:** 10.1038/s41392-025-02331-7

**Published:** 2026-01-13

**Authors:** Yuting Tan, Rui Xue, Yuwei Pan, Zongsheng He, Xiao Hu, Yaping Li, Ke Li, Xuan Zhang, Xiu-wu Bian, Bin Wang

**Affiliations:** 1 School of Medicine, Chongqing University, Chongqing, PR China; 2 Jinfeng Laboratory, Chongqing, PR China; 3 Department of Gastroenterology & Chongqing Key Laboratory of Digestive Malignancies, Daping Hospital, Army Medical University (Third Military Medical University), Chongqing, PR China; 4 Department of Oncology, Chongqing Hospital of Traditional Chinese Medicine, Chongqing, PR China; 5 Institute of Pathology and Southwest Cancer Center, and Key Laboratory of Tumor Immunopathology of Ministry of Education of China, Southwest Hospital, Army Medical University (Third Military Medical University), Chongqing, PR China

**Keywords:** Cancer microenvironment, Tumour immunology

## Abstract

The dynamic interplay between neoplastic cells and the host has been increasingly recognized as important players in the pathogenesis of cancer cachexia, a syndrome affecting ~50–80% of cancer patients with various incidences of different types of malignancies. Despite its prevalence, a comprehensive understanding of cancer cachexia progression, with a holistic view at the cross-organismal, cellular and molecular levels, remains elusive. In this review, we undertake an in-depth exploration of the relevant target organs and their regulatory roles in cancer cachexia, with a particular focus on macroenvironmental interactions *via* various organismal crosstalk axes. Moreover, we highlight how systemic metabolic remodeling, a hallmark of cancer cachexia, plays essential roles in modulating the inflammatory responses of immune and stromal cells in the tumor microenvironment (TME). These cellular responses, in turn, disrupt energy metabolism in distant organs and perturb organismal homeostasis by secreting a variety of mediators that activate specific signaling pathways, thereby fostering a vicious cycle that exacerbates cancer cachexia. We comprehensively summarize these complex cellular and molecular networks that constitute reciprocally regulatory dynamics between systemic metabolic reprogramming and inflammatory cascades. Notably, targeting the multifaceted interplay of organismal metabolic remodeling and cancer-associated inflammation holds great promise for clinical translation, as illustrated by a series of innovative therapeutic strategies and ongoing clinical trials aimed at mitigating cachexia in cancer patients.

## Introduction

Cancer cachexia is a systemic inflammatory response that is commonly characterized by body weight loss and atrophy of muscle and adipose tissues during the progression of cancer. These features stem primarily from increased energy expenditure, hypermetabolism, and anorexia.^1,2^ Clinical criteria have been proposed for individuals with cancer cachexia,^3^ including (1) ≥ 5% weight loss within six months; (2) ≥ 2% weight loss in patients with a body mass index (BMI) < 20 kg/m²; and (3) ≥ 2% weight loss in sarcopenic patients. Some studies have revealed that diagnostic sensitivity differs from cancer type to cancer type. Unsettlingly, the clinical diagnosis of cancer cachexia in obese individuals may be delayed because their high BMI can obscure fat loss and skeletal muscle atrophy,^4^ preventing them from meeting the diagnostic criteria for cachexia. This is particularly concerning in patients with skeletal muscle depletion, which is an independent risk factor for mortality.^5^ Therefore, refining BMI into specifics of body composition, such as lean and fat mass, can be important for achieving more accurate diagnoses. Cancer cachexia negatively affects patients’ quality of life, exacerbates treatment-related toxicity, and significantly increases the mortality rate of cancer by 20–30%.^1,6^ The incidence of cancer cachexia varies among tumor types, with pancreatic, gastrointestinal, and lung cancers being more prominently associated with cachexia, accounting for ~40–70% of all cases.^1,2^ In patients with these tumors, the deterioration of cachexia is accompanied by the progression of cancer, which may be attributed to reduced food intake and deranged digestion, influenced by the tumor in the upper digestive tract.^2^

**Fig. 1:**
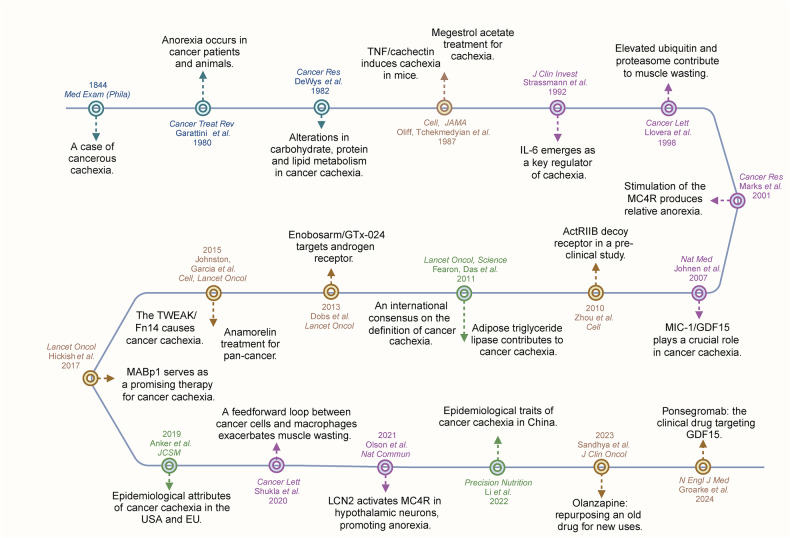
Timeline and milestones in the study of cancer cachexia. The figure presents a comprehensive timeline depicting the significant milestones achieved in cancer cachexia research. Early studies identified the clinical manifestations, underlying mechanisms, animal models, and novel therapeutic strategies. The figure was generated with BioRender (https://biorender.com)

Although cancer cachexia has been documented in humans for centuries, our comprehensive understanding of this disease has emerged only in the last few decades. The term ‘cachexia’ has etymological roots in the ancient Greek lexemes kakós (bad) and hexis (habit), and it refers to the loss of appetite and a general wasting condition. Hippocrates (~460–377 BC) described it as linked to conditions such as hydropsy (edema or fluid retention).^7^ Owing to the relentless efforts and significant research advancements made by numerous scientific researchers, we now have a deeper understanding of cancer cachexia (Fig. 1). Currently, cancer cachexia is recognized as a systemic metabolic syndrome that involves multiple tissues and organs, including musculoskeletal, skeletal, adipose, neurological, gastrointestinal, and hepatic tissues.^8^ This raises the following question: How do tumors affect distant organs? Recently, some studies reported that tumors can communicate with distant organs through neural, blood, and lymphatic networks via metabolites and inflammatory factors.^9,10^ Consequently, both inflammatory cytokines and metabolites may play regulatory roles in mediating cross-organ crosstalk. We hypothesize that catabolism activation and anabolic suppression are important features in cachexia patients and that metabolic remodeling potentially triggers an inflammatory response in various cells. The altered immune and stromal cells then disseminate from primary organs via the circulatory system and secrete various inflammatory factors, such as tumor necrosis factor α (TNF-α), interleukin 6 (IL-6) and members of the transforming growth factor β (TGF-β) family, which induce skeletal muscle and adipose tissue catabolism,^11–13^ forming a regulatory loop. Thus, multiorgan interactions or cellular crosstalk and, consequently, inflammatory factor-mediated systemic perturbations may drive cachexia. In this review, we highlight the importance of crosstalk among distinct organs and metabolite-inflammatory factors in the cachexia macroenvironment and microenvironment.

The tumor microenvironment (TME) is an intricate ecosystem in which cancer cells thrive and can influence tumor growth, metastatic spread, and response to treatment. It comprises mesenchymal elements such as immune cells, cancer-associated fibroblasts (CAFs), endothelial cells (ECs), pericytes, and tissue-specific cells such as adipocytes and neurons, as well as noncellular constituents such as the extracellular matrix (ECM), extracellular vesicles, and soluble factors.^14^ The cancer cachexia microenvironment focuses on the local tissue and cellular milieu where cachexia-related changes occur, including the impact of metabolic products and inflammatory imbalances on cellular signaling pathways and functional states. However, changes in the tumor host body extend beyond the TME. They involve the crosstalk between multiple distal compartments at places beyond tumor beds through hormonal signals, inflammatory mediators, and circulating immune cells,^15^ which also serve as critical contributors to the pathophysiology of cancer cachexia. Understanding the interactions between the microenvironment and macroenvironment offers novel avenues to enhance clinical outcomes in cancer cachexia patients.

## Clinical manifestations and prevalence

### Clinical diagnosis

The diagnostic criteria for cancer cachexia have undergone continuous refinement. The progression in these standards facilitated earlier intervention and management for patients with cachexia. In 2008, a consensus conference on cachexia was held in Washington, D.C., USA, and preliminary diagnostic criteria for this condition were proposed.^16^ Currently, the most widely used diagnostic criteria for cachexia are the European Palliative Care Research Collaboration (EPCRC), upon which cancer cachexia management guidelines have been developed^3^ (Fig. 1). Nevertheless, these criteria incorporate thresholds based on Western populations, which may not accurately reflect the situation for Asians due to variations in body composition, dietary patterns, lifestyles, and metabolic characteristics. To address this issue, the Asian Working Group for Cachexia (AWGC) established the Asian criteria for cachexia: patients with chronic wasting disease and a BMI < 21 kg/m^2^ or weight loss >2% over the preceding 3–6 months, coupled with anorexia, diminished handgrip strength (<28 kg for males and <18 kg for females), or C-reactive protein (CRP) > 0.5 mg/dL.^17^ The differences between the AWGC and EPCRC criteria were the addition of anorexia, grip strength, and CRP level. Xie et al. established the utility of the AWGC2023 criteria in predicting survival and medical burden among Chinese cancer patients.^18^ In a study comparing both the AWGC and EPCRC criteria in lung cancer patients, the AWGC criteria were found to be more effective in diagnosing cancer cachexia in the Asian population and provided superior prognostic indicators.^19^ These findings underscore the pivotal role of racial characteristics in the diagnosis of cachexia and underscore the potential of the AWGC criteria for improved patient assessment and management in Asian populations.

The international consensus group delineated cachexia as a continuous process encompassing three clinically relevant stages: precachexia, cachexia and refractory cachexia^3^ (Fig. 1). On the basis of these stages, Vigano et al. assessed the outcomes of 207 patients with advanced non-small cell lung cancer (NSCLC) or gastrointestinal cancer and reported that precachexia and cachectic patients presented similar outcomes but were significantly different from noncachectic and refractory cachexia patients.^20^ These findings imply that the clinical application of this staging system, such as treatment planning and prognosis assessment, has certain limitations. Notably, there are emerging strategies for staging cachexia. For example, the Glasgow prognostic score, which integrates CRP and albumin levels, provides a straightforward and objective framework for assessing and managing cancer cachexia.^21^ A novel cachexia classification system based on the modified Glasgow prognostic score was used to classify cancer cachexia into four different stages: no cachexia, undernourishment, precachexia, and refractory cachexia. These stages exhibited robust correlations with poor clinical outcomes and demonstrated the capacity to predict overall survival.^22^ Recently, Jin et al. identified cancer cachexia by characterizing longitudinal body composition trajectories and categorized cancer patients into three phases. This classification can also effectively predict survival prognosis and the occurrence of adverse events.^23^ However, the clinical application of these staging systems requires further research.

In summary, the diagnostic criteria for cancer cachexia remain controversial, posing considerable challenges for clinical practice and driving the continuous exploration of novel diagnostic techniques. The diversity and complexity observed in cancer cachexia patients may be attributable to variations across tumor types, ethnicities, and geographical regions.

### Prevalence of cancer cachexia among various geographical regions and cancer types

**Fig. 2:**
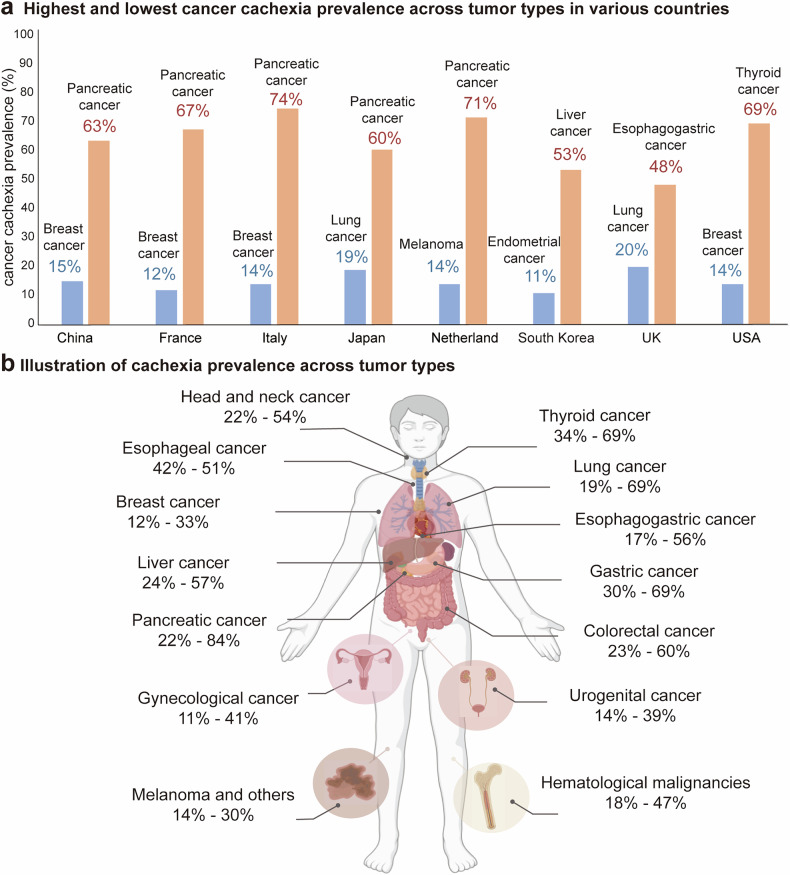
Epidemiological statistics of cancer cachexia patients based on studies published in the past 5 years. The prevalence of cancer cachexia varies by region and tumor type. **a** The prevalence of cachexia among pancreatic cancer patients is high in various countries, and a significantly lower prevalence has been reported in breast cancer patients. In the chart, the blue column highlights the tumor type with the lowest cachexia incidence rate, whereas the yellow column denotes the highest incidence. **b** Digestive system and lung cancer have a high prevalence, whereas breast cancer and melanoma have the lowest prevalence on the basis of available data. The figure was generated with BioRender (https://biorender.com)

The prevalence of cancer cachexia significantly varies among different tumor types and demonstrates geographical disparities across countries and regions (Fig. 2). In 2019, the prevalence of cancer cachexia in the USA and European Union was documented on the basis of 21 studies published between 1980 and 2017 involving 31,047 cancer patients.^24^ A study in China enrolled 47,604 patients with 16 common cancers from June 2012 to December 2020 to investigate the prevalence of cancer cachexia (Fig. 1). These findings indicate that, irrespective of the cancer site, advanced TNM stages are linked to a notably higher incidence of cachexia among the general cancer patient population. Furthermore, compared with younger individuals and males, elderly individuals and males are more likely to develop cachexia.^25^

To obtain more accurate epidemiological data on cancer cachexia, we incorporated dozens of additional studies published in the past five years,^19,25–45^ which provide comprehensive information for the 14 selected cancer entities analyzed (liver cancer, pancreatic cancer, gastric cancer, esophageal cancer, esophagogastric cancer, colorectal cancer, lung cancer, thyroid cancer, head and neck cancer, gynecological tumors, urogenital cancer, hematologic malignancies, breast cancer, melanoma and others) (Fig. 2). These data, unweighted for patient origin, age, tumor stage and treatment strategy, offer a realistic midpoint reflecting clinical practice scenarios. In these studies, the cachexia prevalence in patients with pancreatic cancer was the highest in numerous countries, including China (63%),^25^ France (67%),^24^ Germany (41%), Italy (74%), Japan (60%)^27^ and the Netherlands (71%).^26^ Conversely, the incidence of cachexia in breast cancer patients was lower, with rates of 15% reported in China,^25^ 12% in France,^24^ and 14% reported in both Italy and the USA (Fig. 2a).

In terms of cancer type, the incidence of cachexia was markedly greater in patients diagnosed with pancreatic, gastrointestinal, or lung cancer than in those with various types of cancer. Conversely, the cachexia incidence rate was notably lower in those with breast cancer and melanoma (Fig. 2b). For example, clinical studies reported that cachexia incidence rates in pancreatic cancer patients range from 22% to 84%,^28,29^ whereas melanoma patients presented significantly lower rates, varying between 14% and 30%.^24^ The variations in diagnostic criteria, patient staging, and treatment phases across studies significantly contribute to discrepancies in cachexia prevalence rates. Furthermore, publication biases, particularly the tendency to publish positive results (indicating high prevalence), may introduce limitations in data interpretation. Collectively, these observations underscore the importance of implementing targeted interventions to address cachexia in high-risk cancer patients.

Patients with gastrointestinal cancer are more prone to cachexia, which is potentially attributed to tumor blocking or anatomical changes after esophagogastrostomy, exacerbating the imbalance between inadequate food intake and increased tumor metabolism. Notably, one study evaluated the impact of demographic and socioeconomic factors on cachexia in gastrointestinal tract cancer patients and revealed an elevated risk among socioeconomically disadvantaged and uninsured patients.^41^ Liver cancer, particularly hepatocellular carcinoma (HCC), predominantly arises from cirrhosis, leading to liver dysfunction, such as protein synthesis disorders, and often results in muscle wasting and physical debility in advanced stages. This may explain the high prevalence of cancer cachexia within the liver cancer patient population. Importantly, Rich et al. reported that cachexia and precachexia are prevalent across all HCC stages, including patients with early-stage tumors.^45^ Similarly, cachexia in pancreatic cancer patients is highly prevalent and clinically relevant, primarily because of alterations in both exocrine and endocrine pancreatic functions, as well as potential impacts on the structure of other digestive organs or indirect modifications to gut physiology.^46^

Despite the scarcity of research on thyroid cancer cachexia, the available data revealed a strikingly high prevalence of thyroid cancer cachexia among at-risk patients, with 39.9% and even 69% in one cohort study.^24^ This elevated incidence is partly due to the adverse effects caused by the administration of multikinase inhibitors, which include weight loss, nausea, and diarrhea.^47^ Furthermore, the anatomical location of the tumor, for instance, head and neck cancer, may have an impact on nutritional intake. Malignancy and its treatment frequently compromise essential physiological functions critical for chewing, swallowing, and saliva production, thereby exacerbating the risk of cachexia in patients with thyroid cancer.^48^

The relatively high prevalence of cachexia in lung cancer patients is potentially due to anorexia, cytokines, and metabolic abnormalities.^49^ Interestingly, patients with *epidermal growth factor receptor* (*EGFR)*-mutated lung cancers have a lower risk of developing cachexia, possibly because these cancer cells tend to progress more slowly than those without *EGFR* mutations.^30^ However, comprehensive elucidation of the mechanisms driving cachexia in lung cancer patients may facilitate the development of targeted interventions and better outcomes for this vulnerable population.

Breast cancer patients exhibit the lowest incidence of cancer cachexia and often avoid weight loss after diagnosis. In contrast, they face a risk of weight gain. This may be attributed to reduced metabolism caused by treatment-related ovarian failure and premature menopause, coupled with decreased physical activity after diagnosis.^50^ These findings highlight the necessity of implementing tailored weight management strategies for patients with distinct types of cancer.

In summary, the prevalence of cachexia in these studies was between 11% (endometrial cancer)^24^ and 84% (pancreatic cancer).^28^ Notably, the lack of standardized definitions across the studies precluded precise conclusions. However, we can deduce that the incidence of cancer cachexia is potentially influenced by the unique characteristics of the primary tumor. Therefore, when devising treatment plans, it is imperative to consider the differences among various tumor types.

### Prognostic impacts on clinical outcomes

Cancer cachexia frequently results in adverse prognoses and elevated mortality rates among patients. The 12-month mortality rate for patients with cancer cachexia has reached 30.2%.^51^ After adjusting for various factors, such as age, sex, race/ethnicity, stage, and treatment, cachexia remained an independent predictor of poorer survival across diverse cancer patient populations.^45^ Despite the unclear causal relationship, there is a notable connection between cancer treatment and cachexia. While chemotherapy is effective in improving the 5-year survival probability, it leads to various adverse events, including skeletal muscle deconditioning.^52^ Several studies have indicated that approximately half of patients with advanced colorectal cancer develop cachexia within 24 weeks after receiving first-line systemic chemotherapy.^53^ Importantly, cancer cachexia and its metabolic alterations may impair patients’ tolerance to cancer therapies. Patients with cachexia experience more severe appetite loss and fatigue, potentially disrupting their ability to continue therapy and adversely affecting their well-being and quality of life.^30,31^ These findings underscore the importance of continuous monitoring of cachexia during cancer treatment.

Given the detrimental impacts of cancer cachexia on clinical outcomes, increasing attention has been given to elucidating its pathogenesis. Therefore, our review aims to consolidate and analyze existing research to offer comprehensive insight into the underlying mechanisms of cancer cachexia. However, as mentioned above, the characteristics of cachexia vary among different types of tumors and are closely tied to the primary tumor. Considering the complexity, our discussion will focus primarily on the common mechanisms for most types of cancer cachexia.

## Regulatory roles and interactions of multiple organs in cancer cachexia

**Fig. 3:**
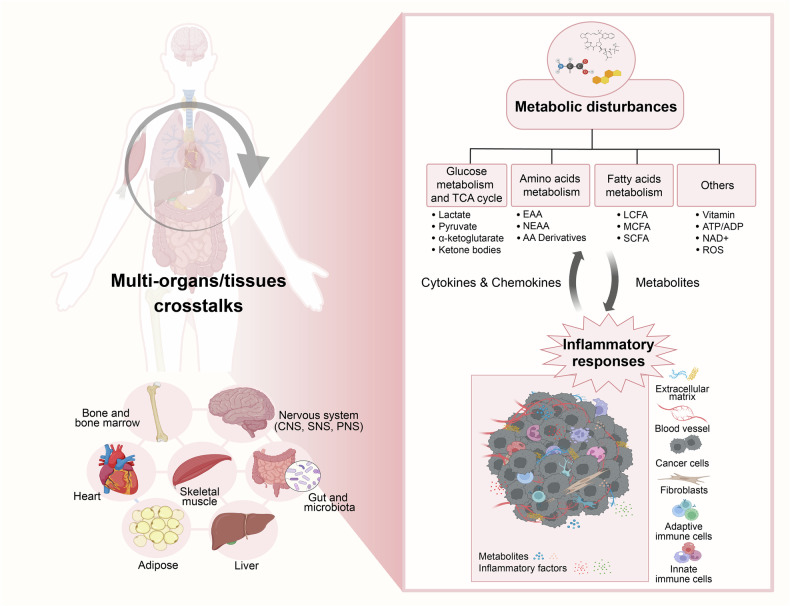
Overview of the interactions between systemic metabolic remodeling and the cellular inflammatory response in cancer cachexia. Crosstalk among various tissues and organs contributes to the complex clinical manifestations observed in cancer cachexia, which is characterized by metabolic disturbances. Systemic metabolic remodeling is potentially associated with the cellular inflammatory response. Subsequently, the cells that undergo functional and quantitative alterations, along with the cachexia-associated factors they secrete, penetrate the vascular system and influence remote target organs/tissues. These inflammatory alterations can lead to a shift toward a catabolic state in terms of systemic metabolism, thereby exacerbating the progression of cancer cachexia. The network diagram above illustrates the intricate interplay between organs and tissues. The left chart below depicts the metabolic reprogramming that occurs in the state of cachexia, whereas the right chart below reveals the specific impacts of these metabolites on various cell types, which in turn affect target organs, further exacerbating the systemic metabolic imbalance. This figure was created with BioRender (https://biorender.com)

Over 3500 years ago, ancient biblical accounts documented a monarch affected by primary carcinoma of the prostate or kidney with subsequent metastases to bones. The textual descriptions—including “I forgot to eat my bread” (anorexia), “My knees are weak through fasting, and my flesh has lost its fatness” (asthenia and adipose depletion), “My strength has failed… and my bones are consumed” (osteolysis), “My bones wasted away through my anguished roaring all day long” (chronic pain-induced demineralization), and “I am feeble and depressed” (affective disturbances)—collectively suggest a clinical presentation congruent with cancer-related cachexia.^54^ It is evident that cancer cachexia, arising from uncontrolled and disrupted interactions among multiple organs or tissues, frequently manifests as systemic metabolic disturbances and augmented inflammatory responses.^1,2,6^ However, the complex mechanism by which diverse organs or tissues drive cancer cachexia through the excretion of various molecular signals and jointly regulating the metabolic and immune landscapes within organs is still poorly understood (Fig. 3). A systematic review of multifactorial alterations, along with their underlying molecular mechanisms, may offer crucial insights for developing therapeutic interventions and optimizing the efficacy of pharmacological treatment for cachexia management in oncology.

### Involvement and regulatory roles of multiple organs

Cachexia involves distinct clinical stages, and each stage can display different clinical symptoms. In the initial stage, patients may experience anorexia and metabolic changes, with insignificant weight loss but gradual adipose tissue depletion. As cachexia progresses, patients experience unconscious weight loss exceeding 5% within six months, accompanied by skeletal muscle atrophy.^3^ Metabolic changes in the liver, such as bile acid, may occur before cachexia, which is attributed to gut microbial dysbiosis.^55^ Therefore, we may conclude that in some cases, gut microbiota disorders may occur before liver metabolic disorders. Petruzzelli et al. identified a phenomenon termed white adipose tissue (WAT) browning, which takes place in the initial stages of cancer cachexia before skeletal muscle atrophy.^56^ A study of 68 pancreatic cancer patients via CT revealed that the loss of subcutaneous adipose tissue was first observed in cancer cachexia patients, followed by skeletal muscle and visceral adipose tissue loss.^57^ This finding was corroborated in a larger cohort of 1690 patients with pancreatic ductal adenocarcinoma (PDAC).^58^ However, another study employing age-, sex-, and race-standardized tissue measurements reported that skeletal muscle decline precedes adipose tissue loss, occurring 18 months and 6 months before pancreatic cancer diagnosis, respectively.^59^ These inconsistencies could be ascribed to differences in evaluation methodologies and sample sizes. Notably, both muscle and bone volume loss become evident in the late stage of cancer cachexia.^58^ In this review, we succinctly outline the crucial roles of target organs in cancer cachexia in the sequence of the nervous system, gut, liver, adipose tissue, skeletal muscle, and bone. However, the sequence of organ involvement may differ on the basis of individual variations and research approaches, necessitating further exploration in future studies to derive more definitive conclusions.

#### Central nervous system dysfunction

The effects of the neuroendocrine system on anorexia and appetite control have garnered considerable attention in cancer cachexia research.^60^ Appetite regulation is involved in many neural circuits and regions within the central nervous system (CNS). Neurons stimulate appetite by secreting neuropeptide Y (NPY) and agouti-related protein (AgRP). In contrast, proopiomelanocortin (POMC) and cocaine- and amphetamine-regulated transcript (CART) neurons exert opposite effects.^61^ POMC and AgRP neurons residing in the hypothalamic arcuate nucleus serve as critical regulators of melanocortin signaling in the CNS, which is associated with cachexia-related appetite dysfunction through POMC activation and AgRP suppression.^62,63^ There are five melanocortin receptors (MCRs) known to exist. Among them, MC3R and MC4R are expressed primarily in the brain. α-MSH is considered an anorexigenic agonist, whereas AgRP is considered an orexigenic antagonist/inverse agonist. The activation of MC4R by α-melanocyte-stimulating hormone (α-MSH) released from POMC neurons inhibits appetite and food intake.^64,65^ Conversely, MC4R antagonists mitigate cancer cachexia through antagonizing central melanocortin signaling, stimulating appetite, and promoting anabolism.^62,63^ Recent research highlighted the anorectic effects of diverse biopeptides, especially lipocalin 2 (LCN2)^66,67^ and glucagon-like peptide-1 (GLP-1),^68^ which directly or indirectly modulate the melanocortin system to suppress appetite. In addition to the melanocortin system, the area postrema and nucleus of the solitary tract (AP/NTS) have also emerged as targets for cancer anorexia-cachexia syndrome. A study demonstrated that pharmacological blockade of brainstem GLP-1 signaling attenuates cachexia in rats.^69^ Similarly, the knockdown of brainstem *prolactin-releasing peptide* reduced weight reduction and appetite suppression in tumor-bearing rats, which might be involved in hypothalamic‒pituitary‒adrenal (HPA) axis inhibition.^70^ Circulating growth differentiation factor 15 (GDF15) traverses the blood‒brain barrier (BBB) to activate the GDNF family receptor alpha-like and Ret proto-oncogene (GFRAL‒RET) receptors in the AP/NTS of the hindbrain, initiating a neural cascade culminating in anorexia through parabrachial nucleus activation.^71^

The current scientific consensus supports a strong correlation between chronic inflammatory processes and anorexia. The hypothalamus, the key regulator of appetite, contains numerous neurons that recognize multiple inflammatory signals, which trigger skeletal muscle atrophy, anorexia, and weight loss.^72^ TIR-domain-containing adaptor-inducing interferon-β (TRIF) has been reported to induce the production of several cytokines and chemokines in the hypothalamus, as well as microglial activation and neutrophil mobilization into the brain. The activation of these inflammatory signals induces anorexia and gastrocnemius catabolism.^73^ Dilp8/INSL3 derived from tumor tissue activates the Lgr3 receptor in the hypothalamus, which increases the expression of anorexigenic nucleobinding 1 and decreases the expression of *the* orexigenic neuropeptides short neuropeptide F (sNPF) and NPF, triggering cachexia-associated anorexia.^74^ In addition to peripheral tissue-derived inflammatory molecules, the neuroimmune axis has recently been implicated in cachexia in mouse models and human specimens. For example, the C-C motif chemokine ligand 2/C-C motif chemokine receptor 2 (CCL2/CCR2) axis recruits neutrophils to the brain to induce anorexia and sarcopenia in patients with pancreatic cancer.^75^ Since inflammation plays a pivotal role in cachexia-associated anorexia, elevated intestinal permeability may represent a potential mechanism underlying central inflammation.

#### Gut microbiota dysbiosis and intestinal barrier disruption

The presence of cancer located outside the gut can disturb the delicate balance of intestinal homeostasis and modify the gut microbiome. By adjusting the gut microbiota composition and fortifying gut barrier function in mice with cachexia, alleviating the distinctive physiological characteristics associated with this condition may be feasible.^76^ For a long period, chemotherapy was previously believed to disrupt intestinal barrier function and the gut microbiota composition among cancer patients, at least in part, by killing intestinal stem cells and the microbiome to affect the mucus layer, epithelium, and immune system.^77^ However, gut barrier dysfunction is also observed in mouse models of cancer cachexia without chemotherapy.^78^ The gut microbiota, as a component of the intestinal microenvironment, regulates host–tumor interactions through the inflammatory response and metabolism regulation. Not surprisingly, gut dysbiosis impairs host immune function by decreasing immune activation markers, including CD11b (innate immune system), CD11c (proinflammatory macrophage), CD3γ (T lymphocytes), Tbet (Th1 lymphocytes), and IL-17A (Th17 lymphocytes).^76^ Therefore, enhancing gut microbial diversity effectively alleviates cachexia-related symptoms partly through regulating the expression of T-cell differentiation-related genes and inflammation-related genes in the colonic mucosa and inhibiting the abundance of FoxP3+ regulatory T cells (Tregs) and Th17 cells in mesenteric lymph nodes.^79^ Additionally, the gut microbiota produces short-chain fatty acids (SCFAs) and other metabolites that support barrier function and energy metabolism.^80^ Given the importance of the intestinal barrier in intestinal homeostasis, an increase in its permeability often leads to systemic inflammation and metabolic disturbances.

#### Hepatic metabolic reprogramming

The liver, as the central organ of metabolism and energy homeostasis, coordinates the intricate balance of energy and nutrient demands by mediating energy status and glucose, amino acid, and lipid metabolism.^81–84^ Rats with cancer cachexia induced by peritoneal carcinosis exhibit impaired hepatic mitochondrial oxidative phosphorylation efficiency, which is attributed to elevated fatty acid incorporation into hepatic mitochondrial cardiolipin, suggesting that there is an increased energy demand for adenosine‒triphosphate (ATP) synthesis.^81^ Reduced glycogen, glucose, and lactate levels, coupled with downregulated glucokinase expression, indicate impaired hepatic glycolysis in patients with cancer cachexia. Despite an overall increase in hepatic amino acid levels, the downregulation of the gluconeogenic enzymes phosphoenolpyruvate kinase and glucose-6-phosphatase in the liver suggests that amino acids are mainly used for biosynthesis of acute-phase reactants rather than gluconeogenesis or tricarboxylic acid (TCA) cycle activity.^82^ Cachexia model mice with colon carcinoma 26 (C26) exhibit severe hepatic steatosis, which is potentially attributed to reduced carnitine levels and hepatic phosphatidylcholine synthesis. The decrease in carnitine levels impairs β-oxidation in mitochondria, making them unable to provide the necessary energy. Moreover, reduced levels of phosphatidylcholine promote triglyceride accumulation in the liver by impeding very low-density lipoprotein-dependent triglyceride export.^82^ Another study reported that the transcription factor TGF-β1-stimulated clone (TSC) 22D4 is increased in the hepatic tissue of individuals with cancer cachexia, which is associated with hepatic lipid accumulation and decreased serum triglyceride levels via the inhibition of hepatic very low-density lipoprotein release and lipogenic gene expression.^83^ Consistent with these findings, hepatic dysfunction is observed in cancer patients with cachexia, as decreased B vitamin-related liver enzymes are detected in blood samples from these patients.^84^ These findings suggest that cachexia is accompanied by a spectrum of metabolic disturbances within the liver. Addressing these metabolic abnormalities may hold potential for both preventive and therapeutic interventions.

#### Fat mobilization and browning

Fat tissue, which is composed of WAT and brown adipose tissue (BAT), serves as a vital metabolic and secretory organ in cancer cachexia. In higher vertebrates, WAT and BAT typically exhibit opposing physiological functions. WAT primarily serves as an energy reservoir, storing triglycerides within white adipocytes. During periods of nutrient scarcity, triglycerides are catalyzed into fatty acids through a lipolytic cascade involving hormone-sensitive lipase (HSL), adipose triglyceride lipase (ATGL), and monoacylglycerol lipase (MGL).^85^ Suppressing lipolysis through targeted genetic inactivation of *Atgl* or *Hsl* can ameliorate certain symptoms of cancer cachexia.^86^ Conversely, BAT dissipates energy as heat during nonshivering thermogenesis, featuring multilocular lipid droplets, abundant mitochondria, and high expression of uncoupling protein 1 (UCP1), contributing to its high metabolic activity.^87^ WAT cells can reversibly transform into BAT cells to adapt to the environment, including cold exposure or some pharmacological agents, such as β3-adrenergic receptor agonists and thiazolidinediones, as well as various peptides and hormones, which is often called “WAT browning”.^56,88^ Locally activated thermogenic adipocytes in the TME not only accelerate cancer progression by providing a fuel source, potentially leading to chemotherapy resistance but also result in weight loss.^89^ Researchers discovered that the complex formed by glucose-regulated protein 75 and adenine nucleotide translocase 2 serves as a critical determinant of UCP1 transcriptional upregulation, ultimately facilitating the browning of adipocytes in cancer cachexia.^90^ However, a previous study demonstrated that neither *Ucp1* knockout nor thermoneutral housing conditions prevented the loss of fat, sparking a debate regarding the importance of *Ucp1*-mediated thermogenesis in cancer cachexia.^91^ While WAT browning positively impacts health by favorably influencing energy expenditure, obesity-related metabolic disorders, insulin resistance, and hyperlipidemia, it has also been implicated as a potential driver of cancer cachexia.^56,88^ The process of WAT browning in cancer cachexia can be triggered by proinflammatory mediators^56^ and hormones (parathyroid hormone/parathyroid hormone-related protein, PTH/PTHrP).^92,93^ Furthermore, adipose tissue is now recognized not only as a fuel reservoir or fat depot but also as an endocrine organ that releases hormones such as adiponectin and leptin, regulating energy balance and inflammation.^94^ In conclusion, adipose tissue loss in cachexia patients involves multiple mechanisms spanning neurological, endocrine, and metabolic domains.

#### Myofiber catabolism induced skeletal muscle atrophy

Skeletal muscle atrophy is the most prominent characteristic of cancer cachexia and is attributed to muscle protein degradation involving multiple molecular pathways.^95^ Notably, the ubiquitin‒proteasome system (UPS) and the autophagy‒lysosome pathway (ALP) play pivotal roles in this process, with calcium (Ca²⁺) serving as a significant modulator.^96^ The UPS is a conserved and dynamic cascade process involving ubiquitin, ubiquitin-activating enzymes (E1), ubiquitin-conjugating enzymes (E2), ubiquitin ligases (E3), and the proteasome. The UPS begins with the ubiquitination of target proteins, after which E3 ligases recognize the degraded protein and ligate ubiquitin to the substrate for degradation.^97^ Two pivotal E3 ligases, i.e., muscle RING-finger protein-1 (MuRF1) and muscle atrophy F-box (MAFbx/Atrogin-1), were shown to regulate skeletal muscle atrophy by triggering the degradation of different muscle proteins. MuRF1 selectively targets sarcomeric proteins such as actin, myosin heavy chain (MHC), and troponin, whereas MAFbx/Atrogin-1 mainly degrades regulatory proteins such as myogenic differentiation (MyoD).^98^ Furthermore, the autophagic machinery orchestrates skeletal muscle atrophy by encapsulating proteins and organelles destined for degradation within autophagosomes. The upregulation of autophagy-related genes such as *Atg5*, *Atg7*, *Beclin1*, and *Lc3b*, along with an increase in the number of autophagosomes observed in cachectic muscle, supports the role of autophagy in atrophy.^99,100^ A clear link was found between alterations in Ca^2+^ homeostasis and decreases in muscle performance. For example, imbalances between calpains and their inhibitors and reduced 130 kDa Ca²^+^-ATPase activity were observed in skeletal muscle or heart tissue in a cancer cachexia model, which suggests that Ca^2+^-dependent proteolysis was activated in tumor-bearing animals.^101^

Cancer-associated skeletal muscle atrophy directly fuels tumor progression by serving as a metabolic substrate for cancer cell proliferation. Specifically, Zhou et al. reported that acetyl-coenzyme A synthetase short-chain family member 2 promotes muscle wasting in pancreatic cancer patients through the GSK3β/TRAIL signaling pathway and augments tumor cell macropinocytosis, a vital mechanism for amino acid supply.^102^ This dual mechanism creates a vicious cycle: muscle wasting supplies tumors with essential nutrients via paracrine signaling and direct metabolic coupling, while tumor-secreted factors further exacerbate muscle catabolism. Consequently, therapeutic strategies targeting muscle atrophy may not only preserve lean body mass but also disrupt tumor metabolic dependency.

#### Bone resorption, marrow adiposity, and hematopoietic failure

Patients with cancer cachexia exhibit aberrant bone metabolism, which is attributed to primary tumors and bone metastases.^103^ Additionally, chemotherapeutic agents such as carboplatin and cisplatin can inflict varying degrees of bone loss.^104,105^ Bone abnormalities are always accompanied by muscle atrophy in various cachexia mouse models.^106^ Cancer cell-derived factors increase the expression of osteoclast markers such as *Acp5*, *CtsK*, *Atp6v0d2*, and *Mmp13* in IDG-SW3 osteocytes, which causes weight loss and muscle atrophy.^107^ Receptor activator of nuclear factor-κB (NF-κb) ligand (RANKL)-induced inflammation is responsible for osteoclast differentiation and bone resorption. Tumor-derived RANKL promotes bone loss and skeletal muscle atrophy in a cachexia mouse model of ovarian cancer.^108^ In addition to peripheral bone destruction, the bone marrow microenvironment may also suffer dysfunction in cancer cachexia. The differentiation of bone marrow hematopoietic stem cells (HSCs) into various immune cells is crucial for systemic immune responses and is tightly regulated by the microenvironment, which includes various cells, such as mesenchymal stem cells (MSCs). MSCs possess multilineage differentiation potential, enabling them to differentiate into various types of tissue cells, encompassing adipogenic, chondrogenic, and osteogenic lineages.^109^ Some inflammatory factors, such as IL-6, can regulate the differentiation trajectory of MSCs, such as toward an adipogenic state, via the Janus kinase/signal transducer and activator of transcription (JAK/STAT) pathway and glucocorticoid signaling.^110^ Osteoclast-releasing factors such as TGF-β can also disrupt the ecological niche of HSCs, leading to cancer-related anemia.^111^ In conclusion, skeletal tissue plays a pivotal role in metabolic and endocrine homeostasis and coordinates the differentiation of various cell types.

### Complex organismal crosstalk *via* diverse axes in cancer cachexia

**Fig. 4:**
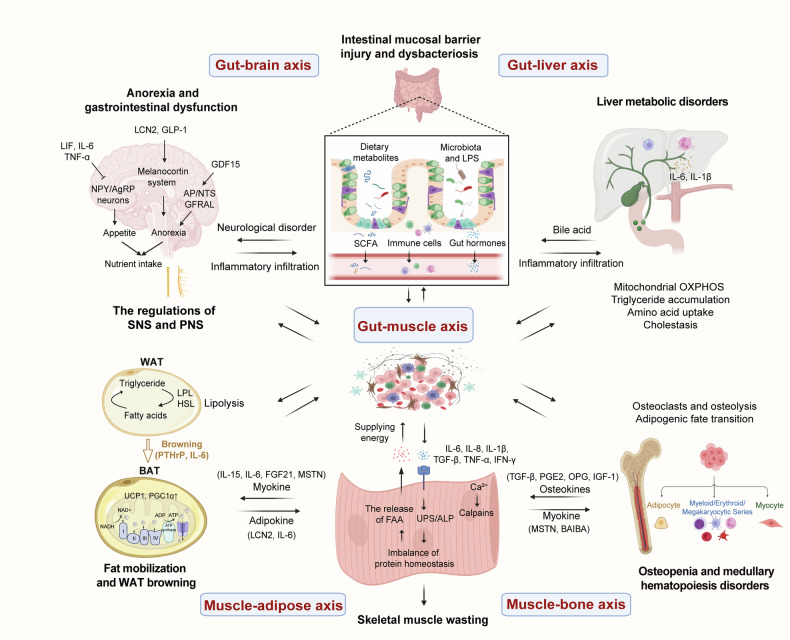
Cross-organismal interactions in cancer cachexia. Cancer cachexia involves multiple organs/tissues, including the nervous system, liver, gut and microbiota, adipose tissue, skeletal muscle, and bone. Notably, the intricate interaction among these organs and tissues exacerbates the abnormal physiological balance of the body, involving the gut‒brain axis, gut‒liver axis, gut‒muscle axis, muscle‒adipose axis, muscle‒bone axis, and dysregulated communication between peripheral tissues and the autonomic nervous system. Furthermore, the interplay of metabolic products and inflammatory signaling pathways within and among these organs creates a complex network, which contributes to the overall decline in body weight and functional capacity observed in cancer cachexia patients. The arrow line represents activation, and the blunt-headed arrow represents inhibition. Abbreviations: OXPHOS oxidative phosphorylation, LPL lipoprotein lipase, FAA free amino acid, BAIBA β-aminoisobutyric acid, OPG osteoprotegerin. This figure was created with BioRender (https://biorender.com)

Cancer cachexia is a multisystem disorder driven by systemic inflammation, metabolic derangements, and neuroendocrine alterations, whose pathological progression is amplified through multiorgan crosstalk. For example, disturbances in gut epithelial tight junction barriers may involve intact bacteria, lipopolysaccharides (LPS), and other bacterial components and digestive enzymes, which further lead to bacterial translocation, immune activation, and systemic inflammation. Notably, inflammation resulting from intestinal dysbiosis often impacts multiple organs throughout the body, including the gut‒brain axis, gut‒liver axis, and gut‒muscle axis, thereby eliciting a cascade effect. Skeletal muscle is the predominant tissue that undergoes catabolic remodeling in cancer cachexia.^96^ Skeletal muscle is no longer considered to be just a motor or energy organ but rather an endocrine organ that produces myokines,^112^ allowing for crosstalk between the muscle and other organs, such as the muscle‒adipose axis and the muscle‒bone axis. Furthermore, the role of the nervous system in influencing peripheral tissues has become increasingly clear in cancer cachexia (Fig. 4).

Although we focused on dissecting the contributions of the gut and skeletal muscle to crosstalk, the importance of other organs in the cachexia process should not be ignored. Focusing on the multiorgan axes of these two organs enables a clearer elucidation of the various axes involved in cancer cachexia.

#### The gut‒brain axis

Multiple lines of evidence implicate gut–brain axis dysfunction as a central mediator in the pathological process of cancer cachexia (Fig. 4). The axis often modulates intricate biological mechanisms via bidirectional communication between the enteric nervous system (ENS) and the CNS, which is facilitated by proinflammatory cytokines, neuropeptides and neurotransmitters, vagal afferents, stress hormones, immune-mediators, SCFAs and other microbiome metabolites.^113^ When intestinal barrier permeability increases, bacterial LPS stimulates the secretion of IL-6 in the hypothalamus, which can be amplified by tumor-derived prostaglandin E2 (PGE2).^114^ Furthermore, increased plasma levels of LPS are related to hypothalamic inflammatory status, where decreased expression of the immune checkpoint receptors programmed death receptor-1 (PD-1) and CD112R, as well as increased levels of corticosterone and LCN2, occurs.^115^ These results indicate that intestinal barrier dysfunction ascribed to gut microbiota alterations may leak LPS into the plasma during cachexia, leading to central inflammation and triggering cachectic phenotypes. Notably, gut dysbiosis not only affects the intestinal barrier but also directly increases BBB permeability. Therapeutic interventions with *Lactobacillus* and sodium butyrate can reduce BBB permeability, upregulate tight junction protein expression, and reinstate endothelial barrier integrity.^116^ Increased BBB permeability may facilitate the penetration of circulating factors, such as GDF15, LCN2, TNF-α, and IL-6, thereby reducing food intake.

Hormones such as ghrelin, cholecystokinin (CCK), GLP-1, and peptide YY (PYY), which are derived from the gastrointestinal tract, have been shown to interact in the modulation of food consumption and body weight. For example, ghrelin participates in cancer cachexia mainly through three mechanisms: (1) stimulation of numerous hypothalamic and brainstem neurons, as well as gastric acid secretion and gastrointestinal motility; (2) increased secretion of growth hormone and reduced energy expenditure; and (3) decreased expression of anorectic cytokines.^117^ Elevated levels of CCK were observed in cachexia models associated with chronic heart failure, leading to cardiac cachexia, which is characterized by significant weight loss and muscle wasting.^118^ Choi et al. reported that Sipjeondaebo-tang, a traditional Chinese medicine, improves cancer-related anorexia and anemia by modulating cytokines (IL-6 and CCL2) and hormones (GLP-1 and PYY) in cachectic tumor-bearing mice, further emphasizing the essential regulatory role of gastrointestinal hormones in cancer cachexia.^119^ The nervous system controls intestinal function, whereas both inflammatory mediators resulting from gut microbiota dysbiosis and gastrointestinal hormones can affect central appetite regulation. These findings underscore the complex bidirectional communication along the gut‒brain axis during cachexia pathogenesis.

#### The gut–liver axis

The gut‒liver axis comprises the biliary tract, portal vein, and systemic circulation. The liver regulates nutrient absorption, metabolism, the intestinal microbiota, and gut wall permeability by releasing bile acids and other bioactive mediators into the intestine. Reciprocally, gut-derived dietary and microbial metabolites modulate bile acid biosynthesis and glucose‒lipid metabolism in the liver via the portal and systemic circulation. During cancer cachexia, the equilibrium of the gut‒liver axis, including the gut microbiota ecology, hepatic function, metabolic processes and immune responses, is frequently perturbed (Fig. 4). Li et al. reported that progressive damage to the intestinal structure during tumor progression in a liver cancer zebrafish model manifested as villus injury, intestinal wall thinning, increased goblet cell numbers, eosinophil infiltration and disrupted intestinal epithelial cell renewal, suggesting that intestinal inflammation may be a promising therapeutic target in cachexia management.^120^ It is conceivable that gut dysbiosis increases intestinal permeability, allowing harmful factors to traverse the gut barrier into the bloodstream and subsequently activate Kupffer cells to release proinflammatory cytokines, thereby initiating hepatic inflammatory responses. Studies have shown that CD68^+^ macrophage infiltration and hepatic inflammasome pathway activation trigger systemic chronic inflammation and cachectic phenotypes by increasing IL-1β activity in liver tissue.^121^

Enterohepatic circulation, as a bridge between the gut and liver, has emerged as an important driver of the progression of cachexia. Farnesoid X receptor (FXR) is widely expressed in the liver and intestine. Activated by bile acid in the gut, FXR induces the expression of fibroblast growth factor 15 (FGF15), which is secreted into the liver via the portal system to activate liver FXR, thus regulating the expression of genes governing enterohepatic bile acid homeostasis.^122,123^ Notably, oral administration of tauroursodeoxycholic acid alleviates cancer cachexia and reverses cholestasis by increasing bile acid secretion in a mouse model.^123^ A recent study similarly highlighted the contribution of gut microbial dysbiosis to perturbing bile acid dysmetabolism in individuals with cancer cachexia, especially the reduction in the levels of several secondary bile acids, mainly taurodeoxycholic bile acids, which occur before the development of cachexia.^55^ These findings underscore the therapeutic potential of taurodeoxycholic acid for hepatic cholesterol homeostasis in cachexia. Abnormal bile secretion may further impact the gut environment, increasing the accumulation of harmful hepatic metabolites and perpetuating the cycle of bile acid production and inflammation. However, Thibaut et al*.* reported that ursodeoxycholic acid-induced bile acid secretion fails to alleviate liver inflammation in cachectic mice but exacerbates muscle wasting due to diminished Takeda G protein-coupled receptor 5 (TGR5) activity, which can promote muscle differentiation and hypertrophy.^124^ The differences in the structural and functional characteristics of bile acids should be further considered.

Intestinal barrier disruption and bile acid metabolism disturbances damage liver function; however, certain factors may safeguard liver functions. Aryl hydrocarbon receptor (AHR), a ligand-dependent transcription factor, senses tryptophan metabolites and promotes intestinal barrier integrity through the upregulation of tight junction proteins and decreased levels of inflammatory factors,^125^ suggesting that the gut‒liver axis is essential for maintaining liver functions. Dolly et al. revealed that, compared with those in normal mice, several indole derivatives and AHR agonists are much lower in the feces of cachectic mice. The authors further reported that the AHR target gene is downregulated in the liver by the IL-6/STAT3/hypoxia-inducible factor 1α pathway. Despite failing to reverse alterations in intestinal permeability or barrier function, AHR agonists ameliorate liver inflammation and glycemic disturbances.^126^

In conclusion, gut microbiota dysbiosis and abnormal bile acid secretion can modify the intestinal milieu, facilitating the translocation of harmful metabolites across the intestinal barrier into the bloodstream. Consequently, immune cells are activated to release proinflammatory cytokines, sparking hepatic inflammatory responses and augmenting the accumulation of deleterious metabolites in the liver. Addressing intestinal permeability or abnormal bile acid secretion may confer protection against cancer cachexia.

#### The gut–muscle axis

Recent studies highlighted the interaction between the gut microbiota and skeletal muscle in cancer cachexia through metabolic and inflammatory regulation (Fig. 4). For example, reduced levels of *Lactobacillus* species were observed in mouse models of cancer cachexia, and *Lactobacillus* supplementation attenuated muscle wasting in these models, suggesting that *Lactobacillus* supplementation may serve as a potential therapeutic avenue for cachexia-related muscle atrophy.^76^ They also maintain gut barrier integrity by reducing intestinal permeability and upregulating antimicrobial proteins,^76^ emphasizing the profound impact of the gut microbiota on skeletal muscle health. Similarly, cisplatin and docetaxel chemotherapy induce cachexia-related muscle wasting in mice by reducing *Ruminococcaceae* and *Bacteroides*.^127^ These studies indicate the presence of widespread gut microbial dysbiosis across various malignancies.

Mechanistically, the gut microbiota plays dual regulatory roles in maintaining the homeostasis of skeletal muscle: (1) modulating nutrient digestion, nutrient absorption^128^ and systemic metabolism through bioactive metabolites^80^ and (2) stimulating host inflammatory responses. Metabolism and inflammation regulation frequently do not function as independent entities but rather interconnect with each other. Fiber supplementation obviously reinforces the colonic mucus layer and reduces circulating LPS-binding protein and IL-6 levels in cachexia model mice, suggesting that the modification of the colonic mucus barrier is a major contributor to the alleviation of systemic inflammation. Furthermore, dietary fiber reverses skeletal muscle wasting by inhibiting Atrogin-1, MuRF1, and autophagy markers such as LC3 and Bnip3.^129^ Conversely, antibiotic-mediated gut dysbiosis and aberrant bile acid metabolism induce muscle atrophy through the repression of skeletal muscle protein synthesis, which is mediated by the FXR-FGF15/19-extracellular-signal-regulated protein kinase (ERK)1/2 signaling pathway.^130^ Under pathological conditions, the endogenous components of the intestinal microbiota, such as LPS and flagella, may mediate inflammatory responses. The release of LPS, which passes through the compromised gut barrier into the circulation, can activate toll-like receptors (TLRs) and subsequently trigger the synthesis of proinflammatory factors, such as IL-6.^131^ TRIF also orchestrates critical signal transduction cascades in TLR responses to LPS.^73^ Once recognized by pattern recognition receptors, gut microbiota-derived flagellin promotes the secretion of CCL2 and IL-6 in C26-induced cachexia models, thereby significantly exacerbating myogenesis in C2C12 myoblasts.^132^ In addition to inflammatory factors, gut barrier disruption promotes the infiltration of eosinophils, M1/M2 macrophages, and fibroblasts, which is accompanied by increased concentrations of IL-13 and TGF-β3 in the colon mucosa, supporting the hypothesis that the intestinal barrier is impaired in cancer cachexia. The depletion of goblet cells in the colon epithelium may further affect the integrity of the mucus barrier.^133^ In summary, inflammatory signals and metabolic imbalance within the context of gut barrier impairment and microbiota dysbiosis exert a long-range effect on skeletal muscle homeostasis, exacerbating their negative nitrogen balance, wherein the synthesis of skeletal muscle proteins falls below their breakdown rates.

#### The muscle‒adipose axis

Skeletal muscle and adipose tissue function as dual-role organs with respect to metabolic and endocrine roles. Muscle‒adipose tissue crosstalk maintains the response to nutritional and physical stimuli. Dysregulation of this crosstalk contributes to the pathogenesis of various diseases, including cancer cachexia (Fig. 4). IL-15, a highly expressed cytokine in muscle tissue, regulates muscle‒adipose interactions by reducing visceral fat mass.^134^ However, Molanouri et al. reported that aerobic interval training and antioxidant therapy prevent muscle atrophy and preserve adipose mass through increased IL-15 expression within the skeletal muscle of cachexia-bearing mice,^135^ highlighting the complexity of IL-15 in regulating adipose tissue. Another myokine, myostatin (MSTN), can cause muscle atrophy, but plasma Mstn concentrations negatively correlate with the development of the cachexia phenotype in colorectal or lung cancer patients.^136^ Consistently, patients with cachexia presented the highest FGF21 levels.^137^ Fu et al. revealed that ablation of FUNDC1 in skeletal muscle, a mitophagy mediator, promotes adipose tissue thermogenesis by increasing FGF21 levels,^138^ suggesting that FGF21-mediated energy metabolism modulates muscle‒adipose interactions. Although this regulatory relationship has not been definitively confirmed in tumors, the possibility cannot be ruled out. In addition to skeletal muscle-derived myokines, adipose tissue also secretes adipokines with potential effects on cachexia (Fig. 4). Adipocyte-derived LCN2 promotes lipolysis and muscle atrophy through transcriptional activation of MuRF-1 and Atrogin-1 expression during pancreatic cancer cachexia.^139^ Some factors, such as FGF21, can be secreted by both skeletal muscle and adipocytes. Under cold conditions, FGF21 derived from BAT serves as a mediator of β3-adrenergic-dependent GDF15 gene transcription and is released in BAT, which suppresses proinflammatory gene expression and decreases TNF and CCL2 secretion in macrophages.^140^ However, GDF15 appears to exert a proinflammatory effect in cancer cachexia,^141^ implying that the same factor can have various effects depending on the physiological or pathological context. Recent studies have demonstrated that tumor epithelial cells not only increase IL-6 production in adipocytes but also increase IL-6R levels in myocytes and subsequently sIL-6R levels in plasma, which induce myotube wasting and adipocyte lipolysis. It highlights the complex crosstalk between tumors, adipose tissue, and muscle.^142^ Another study reported that adipocyte-derived IL-6 promotes adipose tissue inflammation, whereas IL-6 derived from muscle inhibits macrophage infiltration in adipose tissue, triggering an anti-inflammatory response.^143^ These findings indicate that the source of IL6 influences the type of inflammatory response. Overall, energy metabolism disruption and endocrine dysregulation within adipose tissues and muscle are particularly important for the development of many diseases, including cancer cachexia.

#### The muscle‒bone axis

The intricate interplay between bone and skeletal muscle is underscored by their anatomical proximity and reciprocal signaling mediated through bone- and muscle-derived factors (Fig. 4). Research has demonstrated that cisplatin-treated bone-conditioned media elicits profound atrophy in muscle tubules, indicating that bone-derived soluble factors induce muscle wasting. Phosphonates mitigate bone damage and ameliorate muscle atrophy by inhibiting osteoclast-mediated bone resorption.^105^ Within the pathogenic milieu of bone metastases, bone-derived TGF-β contributes to muscle weakness by decreasing Ca^2+^-induced muscle contractility.^144^ Collectively, these findings underscore the implications of bone-derived factors for muscle function, emphasizing that interventions aimed at preventing bone destruction may effectively preserve skeletal muscle mass and safeguard contractile performance.

Notably, the role of muscle‒bone interactions in other physiological and pathological processes may reveal potential roles in cancer cachexia. Osteoblast/osteocyte-derived Connexin43 modulates muscle growth and function, potentially via an endocrine effect of the undercarboxylated isoform of osteocalcin.^145^ Additionally, RANKL inhibitors and osteoprotegerin antagonists synergistically regulate muscle insulin sensitivity and glucose uptake.^146^ These findings should inspire further investigations to explore treatments for muscle diseases characterized by elevated muscle wasting, including cancer cachexia. Conversely, skeletal muscle releases regulatory factors that act on bone. For example, skeletal muscle secretes MSTN to induce RANKL-mediated osteoclast formation in vitro through Smad2-dependent regulation of the nuclear factor of activated T cells.^147^ The exercise-induced muscle factor β-aminoisobutyric acid prevents bone loss and muscle dysfunction by counteracting ROS-mediated mitochondrial breakdown and osteocyte apoptosis.^148^ The regulation of muscle‒bone interactions in cancer cachexia remains elusive, and new investigations should be pursued to reveal the complex mechanisms involved.

#### Regulatory role of the SNS and PNS in several tissues

The CNS, along with the sympathetic nervous system (SNS) and parasympathetic nervous system (PNS), collaboratively maintains body homeostasis by integrating body information, triggering “fight or flight” responses, and promoting rest and digestion, respectively. SNS and the PNS can influence multiple distant organs, especially the CNS, muscles, and adipose tissues (Fig. 4).

Hypothalamic POMC neurons can regulate energy metabolism through SNS activation. This pathway suggests a potential mechanistic link between POMC-mediated SNS hyperactivity and the pathogenesis of cachexia. For example, cannabinoid 1 receptors in the hypothalamus and in the limbic system mediate orexigenic effects. Notably, the conditional knockout of cannabinoid 1 receptors in sympathetic neurons led to sympathetic activation, which resulted in a lean phenotype.^149^ These results demonstrate the importance of SNS activity in cancer cachexia. The vagus nerve, an important component of the PNS, can sense tumor growth potentially via proinflammatory cytokines. This sensory information may subsequently be conveyed to brainstem areas and ultimately to the hypothalamus, triggering the activation of the melanocortin system to decrease food intake.^150^ Indeed, subdiaphragmatic vagotomy can prevent the cancer cachexia phenotype,^151^ which further implies a potential contribution of the vagal PNS to the pathogenesis of cancer cachexia. However, recent research revealed that subdiaphragmatic vagal deafferentation failed to alleviate anorexia or prevent body weight loss, suggesting that vagal afferents may not be obligatory for mediating cancer cachexia syndrome in this experimental model.^152^ These findings collectively underscore the potential interactions with several orexigenic and anorexigenic neuropeptides and hormones, the CNS, the SNS, and the PNS in cancer cachexia.

Some studies have demonstrated that β-2 adrenergic agonists can effectively counteract the accelerated muscle protein catabolism observed in cancer cachexia models, including myocardium and skeletal muscles,^153,154^ suggesting the potential role of SNS activity in modulating skeletal muscle wasting during cancer cachexia. Consistently, a study demonstrated that the use of a neutralizing antibody targeting GDF15 signaling or sympathectomy alone prevented cancer cachexia by reducing excessive β-oxidation in adipose tissue.^155^ Interestingly, Xie et al. reported that increased peripheral sympathetic activity may be induced by intraadipose macrophages. Specifically, IL-4 receptor deficiency hindered the alternative activation of these macrophages, resulting in decreased sympathetic activity and inhibited WAT browning.^156^ These findings suggest that the SNS contributes to the lipolytic response in cancer cachexia. In general, the SNS and PNS, as the two primary constituents of the autonomic nervous system, are extensively distributed across various organs and tissues. They regulate the functional activities of these organs by releasing distinct neurotransmitters, thereby potentially modulating the progression of cachexia.

#### Other cross-organ interactions

In addition to the aforementioned crosstalk, other tissues have intricate interconnections. The heart, as the pivotal organ of the circulatory system, is responsible for circulating blood throughout systemic tissues and organs. Myocardial atrophy, ventricular remodeling, and decreased cardiac function have been consistently documented in murine models of cancer cachexia. These cardiac alterations may be attributed, at least in part, to oxidative stress-mediated pathways.^157^ Compromised cardiac function may lead to inadequate blood supply, thereby exacerbating tissue hypoxia and nutrient deprivation in peripheral organs. Simultaneously, cachexia-induced metabolic reprogramming drives aberrant interorgan communication: the augmented Cori cycle exemplifies this cross-talk, where lactate shuttled from cachectic skeletal muscle to the liver fuels compensatory gluconeogenesis. This diverted metabolic flux not only supports tumor growth but also perpetuates a vicious cycle, which further strains cardiac function through energy-consuming futile cycles and accelerates cachexia progression.^158^

Owing to space limitations, we focus more on elucidating the primary roles of select organs and their interactions in cancer cachexia. Specifically, we focused on molecules beneficial to tissues and organs under other conditions but unreported in cancer cachexia. Furthermore, we observed that the same molecules may exhibit opposite effects in different contexts, on which unraveling the underlying mechanisms offers new insights into the pathology of cachexia. In summary, intricate tissue‒organ interactions are paramount in cancer cachexia; therefore, interfering with these interactions could contribute to the treatment of cachexia.

## Molecular and cellular mechanisms: metabolic alterations, inflammatory responses, key mediators, and signaling pathways

**Fig. 5:**
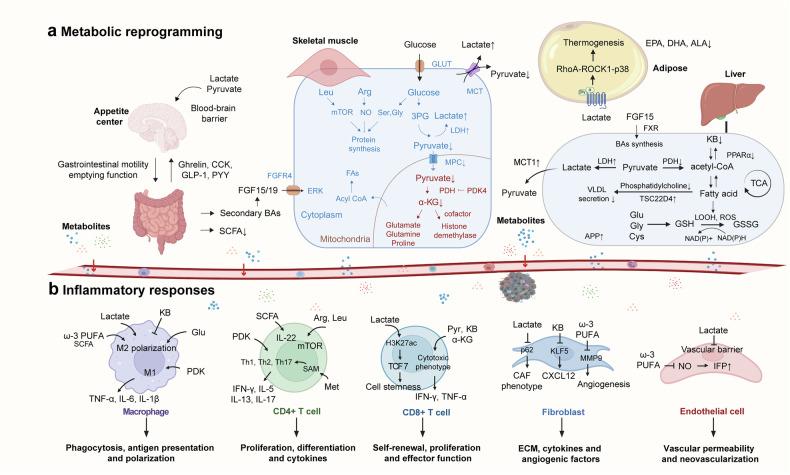
Metabolic remodeling in cancer cachexia and its regulatory effects on cellular functions. **a** The network diagram above illustrates the profound metabolic dysregulation associated with cancer cachexia, such as decreased hepatic ketogenesis, impaired lactate metabolism, elevated hepatic triglyceride accumulation, reduced gut microbiota-derived SCFA production, and suppressed protein synthesis. In glucose metabolism and the TCA cycle, an increase in energy consumption accompanied by an abnormal shift toward anaerobic glycolysis exacerbates the imbalance between energy supply and demand. In fat metabolism, accelerated fat breakdown coexists with WAT browning, resulting in rapid depletion of body fat reserves. In protein metabolism, intensified protein degradation coupled with weakened synthetic capacity leads to significant muscle wasting. **b** The effects of metabolites on immune and stromal cells include phagocytosis and polarization of macrophages, proliferation and effector functions of T cells, ECM production by fibroblasts, and angiogenesis in endothelial cells. The arrow line represents activation, and the blunt-headed arrow represents inhibition. Abbreviations: BAs bile acid, CoA coenzyme A, 3PG 3-phosphoglycerate, GLUT glucose transporter type, GSSG oxidized glutathione, KB ketone body, Pyr pyruvate, α-KG α-ketoglutarate, MPC mitochondrial pyruvate carrier, APP acute phase protein, FAs fatty acids, VLDL very low-density lipoprotein, TSC22D4 transforming growth factor β1-stimulated clone 22D4, Arg arginine, Leu leucine, Met methionine, Ser serine, Gly glycine, Cys cysteine, Glu glutamate, SAM S-adenosylmethionine, TCF7 transcription factor 7, MMP9 matrix metalloproteinases-9, IFP interstitial fluid pressure. This figure was created with BioRender (https://biorender.com)

**Fig. 6:**
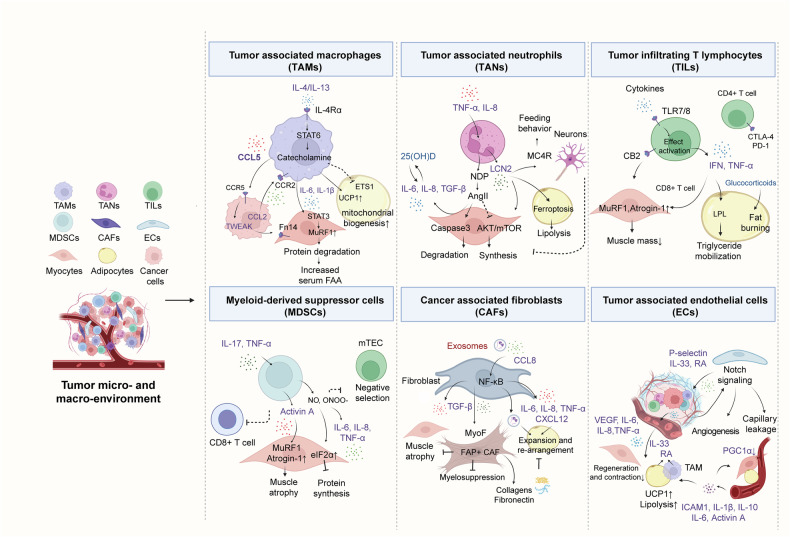
Tumor-associated inflammation alters organismal metabolic status in cancer cachexia. Cancer cachexia is closely associated with the dysfunction of multiple cell types, including TAMs, TANs, TILs, MDSCs, CAFs, and ECs, all of which are currently the subject of extensive research interest. The metabolic burden on these cells fosters a macroenvironment that is more conducive to tumor growth and the progression of cachexia. Mechanistically, they reach distant organs/tissues through the circulatory system to exert their effects or exert a broader influence by releasing cachexia-related factors. The arrow line represents activation, and the blunt-headed arrow represents inhibition. Abbreviations: ETS1 V-et erythroblastosis virus E26 oncogene homolog 1, NDP neutrophil-derived proteases, FAA free amino acid, CB2 cannabinoid 2, LPL lipoprotein lipase, mTEC medullary thymic epithelial cell, ONOO^-^ peroxynitrite, eIF2α eukaryotic translation initiation factor 2α, MyoF myofibroblast, RA retinoic acid, ICAM1 intercellular cell adhesion molecule-1. This figure was created with BioRender (https://biorender.com)

**Fig. 7:**
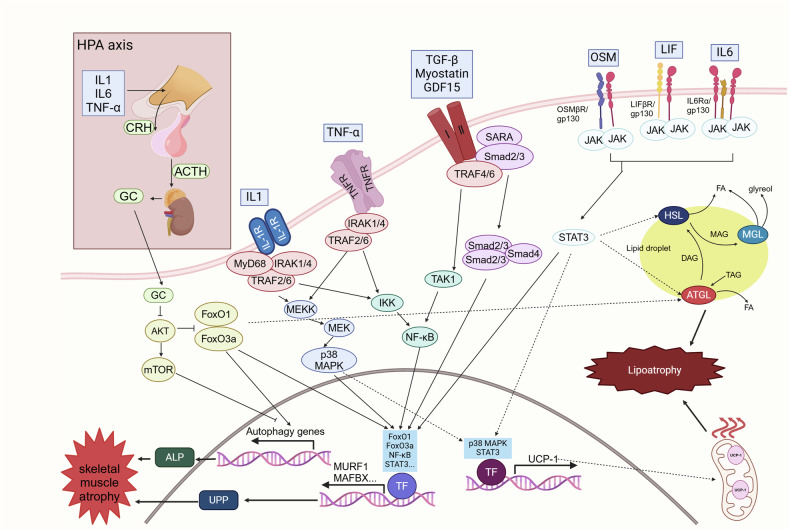
Inflammatory ligand-activated signaling pathways in cachexia-related muscle and fat atrophy. In patients with cachexia, elevated cytokine levels activate or inhibit multiple signaling pathways, leading to muscle and fat wasting. IL-1, IL-6, and TNF-α stimulate the release of CRH in the hypothalamus, which promotes the secretion of glucocorticoids through the HPA axis. This process inhibits the Akt signaling pathway while upregulating the expression of MuRF1, MAFbx, and autophagy-related genes. IL-1 and TNF-α recruit TRAF2/6 to activate the MAPK and NF-κB signaling pathways, facilitating the nuclear translocation of p38 MAPK and NF-κB, respectively, which upregulates the expression of MuRF1 and MAFbx. The TGF-β family engages its receptors, recruiting TRAF4/6 and Smad2/3 to activate the NF-κB and Smad signaling pathways. This promotes the nuclear translocation of the NF-κB and Smad2/3/4 complexes, increasing the expression of MuRF1 and MAFbx. The IL-6 family binds to its receptors to activate the JAK/STAT signaling pathway, leading to the nuclear translocation of STAT3 and the subsequent upregulation of MuRF1 and MAFbx expression. Moreover, the nuclear translocation of p38 MAPK and STAT3 induced by IL-1, TNF-α, and the IL-6 family can also upregulate UCP-1 expression, contributing to fat wasting through mitochondrial involvement. Additionally, the IL-1-, TNF-α-, and IL-6-mediated FoxO and STAT3 pathways directly participate in ATGL- and HSL-dependent lipolysis. This figure was created with BioRender (https://biorender.com)

The extensively reported organ interactions involved in cancer cachexia, such as the gut‒brain axis, gut‒liver axis, gut‒muscle axis, muscle‒adipose axis, and muscle‒bone axis, and the regulatory role of the SNS and PNS in several tissues have been thoroughly described in recent decades. However, inflammation and metabolic disorders are intertwined in the crosstalk of these organs/tissues, which raises the chicken-and-egg question of whether it is a disturbance of metabolism or inflammation that initiates cancer cachexia. Metabolic disorders, characterized by disruptions in carbohydrates, lipids, and proteins, play essential roles in cancer cachexia.^159^ The notable abnormalities in host metabolism diminish nutrient and oxygen availability within the TME and lead to the accumulation of metabolic byproducts, which undoubtedly pose additional threats to cellular health and function. The increased metabolic burden subsequently accelerates cellular damage and dysfunction, resulting in inflammatory responses among immune cells and stromal cells (Fig. 5). In this scenario, the involved cells release certain cachexia-inducing factors (Fig. 6), which disrupt homeostasis by activating relevant signaling pathways (Fig. 7) and ultimately drive the aggressive progression of cachexia. The formation of the feedback loop underscores the intricate interplay between cellular functional transformation and dynamic metabolic‒inflammatory interactions during cancer cachexia progression.

### Alterations in metabolites and their potential impacts on immune and stromal cells

Metabolic imbalance in cancer cachexia cannot be overlooked, as it may reshape cellular function and exacerbate the progression of cachexia. Macroenvironmental metabolites are crucial for maintaining homeostasis and serve as essential constituents of energy sources. In addition to their classic roles via the allosteric regulation of enzymes involved in metabolism, some metabolites also convey a specific signaling message through protein receptors.^160^ Furthermore, metabolites function as substrates or cofactors for chromatin-modifying enzymes, and they regulate gene expression through both transcriptional and epigenetic mechanisms.^161^ Importantly, the cellular inflammatory response is considered a key hallmark of cancer progression and can be induced by abnormal metabolism. Given the vast array of metabolites, we classified them into three main categories: glucose metabolism and the TCA cycle, amino acid metabolism, and fatty acid metabolism. Variations in the dynamic levels of these components can result in modifications to the immune response and stromal cell functionality, thereby fostering a microenvironment conducive to tumorigenesis (Table 1). Thus, metabolic imbalance and inflammatory responses in cancer cachexia play pivotal roles in shaping disease progression.

**Table 1:** Regulatory effects of systemic metabolites on immune cells and stromal cells in the TME

Cell types	Metabolites	Cellular functions	Major Mechanisms	References
Macrophages	Lactate	Promote M2 macrophage transition; drive expression of M2-like genes during M1 macrophage polarization	Enhancing H3K9ac of M2-associated gene; promoting histone lysine lactylation	^175,176^
	Pyruvate	Upregulate PD-L1 in TAMs; reduce infiltration of CD45^+^ immune cells, cytotoxic T cells, and NK cells	Intervening PC under hypoxic conditions	^184^
	Alpha -ketoglutarate	Activate M2 phenotype and suppress proinflammatory cytokine production	Activating mTORC1 signaling	^189^
	Glutamine	Enhance M2 polarization and CCL22 secretion; repress TNF production, lysosomal function, and antigen presentation	Maintaining TCA cycle activity; inhibiting the NF-κB, and enhancing STAT3 signaling	^207,208^
	ω-3 PUFA	Reduce COX-2 expression and PGE2 synthesis; inhibit the secretions of TNF-α and IL-6	Inhibiting the phosphorylations of IKKβ and JNK, preventing IκB degradation; repressing TLR4 signaling via Akt/JNK phosphorylation and the nuclear translocation of p65	^257^
	MCFAs	Enhance inflammatory responses and macrophage phagocytosis	Activating GPR84 signaling	^264^
CD4^+^ T cells	Alpha -ketoglutarate	Attenuate FoxP3^+^ Treg differentiation and increase inflammatory cytokines (e.g., TNF, GM-CSF, IFN-γ, and IL-17A)	Increasing fatty acid synthesis, triacylglycerol stores, and OXPHOS	^190^
	Methionine	Induce differentiation; secrete higher levels of IFN-γ	Generating methyl donors to sustain RNA, protein, and DNA synthesis	^209^
	SCFA	Promote IL-22 production that protects intestines from inflammation	Increasing the accessibility of HIF1α-binding sites in the *Il22* promoter through histone modification	^266^
CD8^+^ T cells	Lactate	Promote cell stemness and efficacy of anti-PD-1 therapy; diminish IFN-γ production	Enhancing H3K27ac of the *Tcf7*; disturbing glycolysis and NFAT translocation	^174,177^
	Pyruvate	Reduce cytotoxicity; support CD8^+^ T-cell antitumor function	Inhibiting autocrine signaling through SUCNR1 by reducing PC activity; MPC facilitates nutritional metabolism	^183,185^
	Alpha -ketoglutarate	Increase infiltration of IFNγ ^+^ CD8 ^+^ T cells	Activating STAT1 to promote PD-L1 expression	^188^
	Ketone bodies	Enhance metabolism of CD8^+^ T cells; increase production of inflammatory cytokines; enhance anti-tumor immunity	Increasing permissive H3K27Ac at effector gene loci (e.g., *Ifng*, *Gzmb*, *Tbx21*); promoting the synthesis of TCA cycle	^199^
NK cells	Lactate	Diminish IFN-γ production	Disturbing glycolysis and NFAT translocation	^177^
	ω-3 PUFA	Increase cytotoxicity and IFN-γ production	Promoting mitochondrial OXPHOS activity by regulating PGC-1α expression	^258^
T cells	Arginine	Promote T-cell proliferation and cytokines production such as IFN-γ, IL-5, and IL-10	Facilitating the synthesis of CD3ζ or other components of the TCR	^205^
	Glutathione	T-cell activation and proliferation; Th17 development and Th17-driven CNS inflammation	Producing GSH and suppressing ROS upon TCR stimulation	^206^
MDSCs	Glutamine	Increase generation and recruitment of MDSCs	Increasing active caspase-3 on MDSCs and CSF3 expression in tumor cells.	^208^
Dendritic cells	Alpha -ketoglutarate	Promote activation of DC; increase proinflammatory cytokines CCL5 and CXCL10	Reducing autophagy in tumor cells	^188^
	ω-6 PUFA	Inhibit differentiation and maturation	Skewing dendritic cell metabolism toward glycolysis and reducing immune stimulation	^259^
Fibroblasts	Lactate	Stimulate CAF phenotype	Downregulating p62 transcriptionally	^178^
	Ketone bodies	Inhibit CAF proliferation and increase NK and cytotoxic T-cell infiltration	Suppressing CXCL12 expression in CAFs and CXCL12/CXCR4/CXCR7 signaling in tumor cells	^198^
Endothelial cells	ω-3 PUFA	Enhance anticancer drug delivery efficiency	Disrupting the NO signaling cascades, lowering IFP	^260^

*H3K9ac* lysine 9 acetylation*, PD-L1* programmed death ligand 1, *TAM* tumor-associated macrophages, *PC* pyruvate carboxylase, *mTORC1* mechanistic target of rapamycin complex 1, *CCL22* C-C motif chemokine ligand 22, *TNF* tumor necrosis factor, *TCA* tricarboxylic acid cycle, *NF-κB* nuclear factor kappa-light-chain-enhancer of activated B cells, *STAT3* signal transducer and activator of transcription 3, *PUFA* polyunsaturated fatty acid, *COX-2* cyclooxygenase-2, *PGE2* prostaglandin E2, *IL6* interleukin-6, *IKKβ* inhibitory kappa B kinase beta, *JNK* c-Jun N-terminal kinase 22, *IκB* inhibitors of kappa B, *TLR4* toll-like receptor 4, *Akt* protein kinase B, *p65* NF-κBp65, *GPR84* G protein-coupled receptor 84, *FoxP3* forkhead box protein P3, *GM-CSF* granulocyte‒macrophage colony-stimulating factor, *IFNγ* interferon-γ, *OXPHOS* oxidative phosphorylation, *SCFA* short-chain fatty acid, *HIF1α* hypoxia-inducible factor, *TCF7* transcription factor 7, *NFAT* nuclear factor of activated T cells, *SUCNR1* succinate receptor 1, *MPC* mitochondrial pyruvate carrier, *Ifng* interferon gamma, *Gzmb* granzyme B, *Tbx21* T-box

#### Glucose metabolism and the TCA cycle

Metabolites constitute an extensive and intricate system that is indispensable for understanding numerous diseases, ranging from traditional metabolic disorders to cancers. During the metabolic process, they not only release energy to serve as a power source but also modulate enzyme activity through intricate feedback mechanisms governing key metabolic pathways to influence the metabolic rate. Crucially, cancer metabolites can affect epigenetic modifications, posttranscriptional modifications, and signal transduction processes.^162^ In this discussion, we focus on lactate, pyruvate, alpha-ketoglutarate, and ketone bodies, which are frequently reported in the context of cancer cachexia.

##### Lactate

Organisms harness energy from glucose via fermentation and respiration, both of which initiate glycolysis to yield pyruvate. During fermentation, lactic dehydrogenase (LDH) reduces pyruvate to lactate, which is then expelled into the cytoplasm. The oxidative respiration channel nicotinamide adenine dinucleotide reduces (NADH) and pyruvate to the mitochondria, which is facilitated by pyruvate dehydrogenase (PDH).^163^ Warburg’s seminal work in the 1920s highlighted that cancer cells preferentially convert glycolytic pyruvate to lactate, even under aerobic conditions.^164^

During cancer cachexia progression, abnormal metabolism of lactate can occur in various tissues, including skeletal muscle, adipose tissue, the liver, and the CNS (Fig. 5). LDH reportedly correlates with systemic inflammation and survival rates in advanced cancer patients.^165^ The decreased activity of PDH and succinate dehydrogenase in cachectic skeletal muscle indicates a reduced flux of glycolytic pyruvate into the TCA cycle.^166^ Under conditions of metabolic stress, such as nutrient deprivation and hypoxia, a reduction in PDH activity is anticipated. This reduction occurs due to the inactivation of PDH by PDH kinase (PDK) 4, which is upregulated in cancer cachexia. This upregulation can subsequently lead to the shrinkage of C2C12 myotubes and the induction of muscle atrophy in vivo.^167^ Increased glucose uptake, decreased oxygen consumption, upregulated LDH expression, and enhanced lactate production in cachectic myotubes and adipose tissue were identified. Inhibition of LDH can alleviate the cancer cachexia phenotype.^168^ Monocarboxylate transporters (MCTs) 1--4 are proton-dependent membrane proteins that serve as critical mediators of transmembrane flux for glycolysis-derived metabolites (i.e., lactate and pyruvate) and ketone bodies (i.e., acetoacetate and β-hydroxybutyrate).^169^ Lactate exerts metabolic effects and activates signaling cascades in neurons through transmembrane transport via MCT2 or by acting on specific receptors,^170^ implicating the critical modulator of lactate in the nervous system. The infusion of lactate into mice via the hepatic portal vein or the vena cava augments postprandial satiety and reduces feeding, implying that increased peripheral lactate may induce neurogenic anorexia, promoting weight loss and tissue wasting.^171^

The multifaceted roles beyond the energy supply of lactate have been well elucidated. For example, lactate-induced G protein-coupled receptor (GPR) 81-mediated cachexia in adipose tissue occurs via the Gi-Gβγ-RhoA/ROCK1-p38 signaling cascade. This cascade enhances adipose browning and lipolysis and ultimately leads to muscle wasting and systemic hypercatabolism.^172^ Lactate and other metabolites may also modulate autophagy through reactive oxygen species (ROS)-mediated signaling cascades involving ERK1/2/mammalian target of rapamycin (mTOR)/p70S6K.^173^

The increased levels of lactate in the circulatory system are closely related to the deterioration of cachexia. Furthermore, at the cellular level, increased lactate profoundly influences diverse cellular states and functionalities (Table 1). At relatively high concentrations, lactate enhances histone H3 lysine 27 acetylation (H3K27ac) at *Tcf7* superenhancer sites by inhibiting histone deacetylase activity, thereby increasing *Tcf7* gene expression in CD8^+^ T cells to increase their stemness and improve the efficacy of anti-PD-1 therapy.^174^ The addition of lactate to glucose-starved mouse bone marrow-derived macrophages (BMDMs) increases H3K9ac at promoter regions of typical M2-associated genes, indicating an epigenetic role of lactate in promoting macrophage polarization and immunosuppressive function.^175^ Furthermore, lactylation, identified as a novel epigenetic modification of histone lysine residues, can also directly stimulate gene transcription in mouse and human cells, such as arginase 1 expression in mouse BMDMs.^176^ Lactate also inhibits tumor surveillance by suppressing the function of natural killer (NK) cells.^177^ Linares et al. demonstrated that lactate impairs poly(ADP‒ribose) polymerase 1 (PARP-1) activity by reducing the NAD^+^/NADH ratio. PARP-1 inhibition blocks the poly(ADP-ribosyl)ation of c-FOS and c-JUN, leading to p62 downregulation, which is associated with the induction of the CAF phenotype.^178^ Furthermore, several studies have shown that lactic acidosis in hypoxic tumor tissue can activate TGF-β signaling, which subsequently stimulates neovascularization by regulating the expression of vascular endothelial growth factor (VEGF) and matrix metalloproteinases. This process facilitates tumor cell invasion and progression.^179^

Elevated lactate levels in cancer cachexia can induce skeletal muscle atrophy, anorexia nervosa, hepatic energy deficiency, and WAT browning. Various studies have demonstrated that lactate in the microenvironment impacts multiple cellular functions, including but not limited to T cells, NK cells, macrophages, fibroblasts, and ECs. However, these findings have yet to be confirmed in cancer cachexia patients and necessitate further investigation.

##### Pyruvate

Pyruvate, along with its corresponding metabolic enzymes, can restore energy balance by entering the TCA cycle and therefore regulate genes associated with skeletal muscle differentiation and degradation, ultimately impacting cancer cachexia (Fig. 5). Myotubes treated with conditioned media containing sodium pyruvate from CT26 colon carcinoma cells presented normalized lactic acid production, oxygen consumption, and PDH activity. Notably, the mitochondrial pyruvate carrier serves as the sole entry point for the entry of pyruvate into mitochondria. Inhibition of the mitochondrial pyruvate carrier in myotubes recapitulates metabolic perturbations and cachectic traits in addition to those observed with conditioned media from CT26 cells, thereby emphasizing the importance of the mitochondrial pyruvate content in mitigating cachexia.^180^ A previous study revealed that PDHB knockout causes lactate accumulation by reducing pyruvate metabolism and suppressing genes related to muscle differentiation. Ariadne RBR E3 ubiquitin protein ligase 2 is a pivotal downstream mediator in this process and is involved in aging-associated muscle degeneration and ubiquitin-dependent modification in skeletal muscle.^181^ PDK4 promotes gluconeogenesis through the preservation of PDH substrates such as pyruvate, lactate, and alanine. However, recent experimental evidence has demonstrated that virus-mediated PDK4 overexpression in myotube cultures is sufficient to promote myofiber shrinkage, which is consistent with increased protein catabolism and mitochondrial abnormalities.^167^

Multiple studies have highlighted the effects of pyruvate metabolism on functional phenotypes across diverse cellular populations (Table 1), such as macrophage polarization, mitochondrial dynamics, and the formation of mitochondria-associated membranes, all of which are indispensable for macrophage function. T-cell differentiation is also linked to pyruvate metabolism, with PDK inhibiting pyruvate oxidation and promoting proinflammatory T-cell polarization.^182^ In cytotoxic CD8^+^ T cells, pyruvate fuels the TCA cycle via pyruvate carboxylase and PDH. Pyruvate carboxylase, a pivotal gluconeogenic enzyme, converts pyruvate to mitochondrial oxaloacetate, which maintains succinate secretion and initiates autocrine signaling through succinate receptor 1, triggering the production of cytotoxic molecules in T cells to augment their tumoricidal capacity. Conversely, tumor-derived lactate perturbs pyruvate flux within the TCA cycle, favoring PDH-mediated reactions over pyruvate carboxylase activity, highlighting the metabolic orchestration of the TME.^183^ Similarly, pyruvate carboxylase inhibition in TAMs induced by a hypoxic tumor environment has immunosuppressive effects on TAMs, which is mainly mediated by CD8^+^ T cells and macrophages.^184^ Intriguingly, studies have shown that inhibiting mitochondrial pyruvate import in CD8^+^ T cells promotes glutamine and fatty acid oxidation, leading to elevated CoA levels, particularly H3K27ac, which enhances histone acetylation and chromatin accessibility. This shift facilitates CD8^+^ T-cell differentiation toward memory T cells, partly through the transcriptional regulation of RUNX1.^185^

Pyruvate metabolism dysfunction in cancer cachexia involves multiple enzymes, such as PDH, PDK and pyruvate carboxylase, which affect metabolite levels along the metabolic axis and orchestrate critical processes in myogenic differentiation and muscle proteolysis. Further exploration of the cellular functions of pyruvate in the progression of cancer cachexia is necessary.

##### ***Alpha(*α*)-ketoglutarate***

α-ketoglutarate, a crucial intermediate in the TCA cycle and a product of glutamine deamination, inversely correlates with glucose levels in the gastrocnemius muscle of cachectic mice.^186^ α-ketoglutarate plays a protective role in muscle development, such as promoting C2C12 myoblast proliferation, alleviating glucose deprivation-induced myotube atrophy, and maintaining the antioxidant capacity of cells.^186^ Additionally, Ruiz et al. discovered that α-ketoglutarate, as a cofactor for histone demethylases, inhibits TNF family member *Tnfrsf12a/Fn14* expression-induced cachectic manifestations by reversing the glutamine deficiency-induced tri-methylation of lysine 4 on histone H3 (H3K4me3) enrichment at the *Tnfrsf12a* promoter, indicating the protective role of dimethyl α-ketoglutarate^187^ (Fig. 5).

Dimethyl-α-ketoglutarate reshapes the tumor immune microenvironment by enhancing radiation therapy-induced tumor cell apoptosis and immunogenic cell death, as well as increasing CD8^+^ T-cell infiltration.^188^ However, contrary findings suggest that α-ketoglutarate production activates mTORC1 signaling and sustains an immunosuppressive M2-like macrophage phenotype.^189^ Moreover, α-ketoglutarate significantly alters the DNA methylation profile of naïve CD4^+^ T cells, attenuating the differentiation of Tregs and enhancing the production of inflammatory cytokines and antitumor cytotoxicity.^190^ This multifaceted role of α-ketoglutarate underscores its complexity in regulating immune responses and metabolic pathways in diverse biological contexts (Table 1).

##### ***Ketone bodies***

Ketone bodies, consisting of β-hydroxybutyrate, acetoacetate, and acetone, are intermediary products of fatty acid oxidation and decomposition. Studies have revealed decreased hepatic ketogenesis in cachectic mice compared with healthy food-restricted or starved counterparts, indicating their roles in cancer cachexia (Fig. 5). Indeed, 2-deoxy-D-glucose supplementation enhances hepatic ketogenesis and promotes ketone metabolism in skeletal muscle, which elevates ATP synthesis efficiency and blocks aberrant Cori cycling, thereby effectively preventing muscle atrophy.^158^ Tumor-secreted IL-6 inhibits peroxisome proliferator-activated receptor (PPAR) α, a transcriptional regulator of ketogenesis, diminishing hepatic ketogenic capacity in precachectic mice. This metabolic reprogramming and energy deficit impair the ability of the host to provide alternative endogenous energy sources. More importantly, it exacerbates the stress response, leading to elevated glucocorticoids and potentially undermining the effectiveness of antitumor immunotherapy.^191^ In contrast, a PPARα agonist restores ketone production, mitigating skeletal muscle and body weight loss.^192^ Moreover, research in gastrointestinal cancer patients revealed upregulated serum ketone bodies, potentially driven by increased lipolysis and amino acid breakdown.^193^ Increased β-hydroxybutyrate in the liver, portal vein, and systemic circulation of cachectic mice is accompanied by a reduction in hepatic ketogenesis transcripts, suggesting the activation of alternative ketogenesis pathways such as kidney ketogenesis.^82^ Brain glucose utilization is decreased in tumor-bearing animals, potentially substituted by lactate and 3-hydroxybutyrate, implying a shift to ketone bodies as primary brain energy sources.^194^

Research on ketogenic diets in cachexia patients underscores the transition of energy supply from glucose to fatty acids, which provides therapeutic benefits for cancer cachexia through the targeting of ketone bodies. Supplementation of CT26 cell cultures with 3-hydroxybutyrate antagonizes tumor-induced C2C12 myotube atrophy by increasing TCA cycle activity, protein synthesis, and metabolic balance while attenuating proteolysis and inflammation.^195^ Cumulative evidence indicates that diets fostering ketone body production and anti-inflammatory strategies may serve as promising preventive measures for cancer cachexia patients.^196^ Nevertheless, the effects of ketone bodies are not uniformly beneficial. Ketogenic diet accelerates cachexia onset and shortens survival in IL-6-related cancer cachexia mouse models. This effect may be attributed to increased lipid hydroperoxide (LOOH) production, which saturates the glutathione (GSH) pathway, leading to ferroptosis in cancer cells. Additionally, redox imbalance and NADPH depletion impair the biosynthesis of cortisol, a vital metabolic stress regulator in the adrenal glands, causing relative adrenal insufficiency and metabolic maladaptation in ketogenic diet-fed mice.^197^

Ketone bodies can regulate the functions of different cells. β-hydroxybutyrate levels are reduced in human CRC, whereas increased ketogenesis inhibits KLF5 expression in CAFs by suppressing histone deacetylase 1, causing the downregulation of C-X-C motif chemokine ligand 12 (CXCL12). This mechanism, in turn, inhibits M2 macrophage accumulation, promotes NK cell and cytotoxic T-cell infiltration, and augments the efficacy of anti-PD1 therapy.^198^ Similarly, ketone bodies are essential fuels that support CD8^+^ T-cell metabolism and effector function by enhancing mitochondrial respiratory capacity and TCA cycle synthesis. Furthermore, β-hydroxybutyrate serves as the primary source of acetyl-CoA in T cells, facilitating H3K27ac at promoters of effector gene loci (e.g., *Ifng*, *Gzmb* and *Tbx21*), thereby augmenting cytokine production in CD8^+^ T cells (e.g., IFN-γ, TNF-α) and modulating cellular immune functions.^199^ These findings stress the importance of ketone body supplementation in enhancing antitumor immunity (Table 1).

Decreased hepatic ketogenesis in cancer cachexia patients results in an energy deficit and impairs antitumor immunotherapy efficacy, which aligns with reports that ketones enhance antitumor immunity. While some conflicting results exist, the clinical utility of ketogenic therapy for treating cancer cachexia has been demonstrated.

#### Amino acid metabolism

In addition to intermediate metabolites, amino acids and their derivatives play pivotal roles in metabolic reprogramming, and their metabolic disorders are associated with numerous pathological conditions. Tumor cells exhibit a unique capacity to redirect carbohydrate intermediates from oxidative phosphorylation to various anabolic pathways, a phenomenon known as the Warburg effect,^164^ but emerging evidence underscores the crucial involvement of amino acids, the fundamental building blocks of proteins, in the Warburg effect.^200^ Numerous strategies targeting amino acid depletion have been proposed to repress cancer progression by modulating amino acid transporters, inhibiting amino acid biosynthesis, or consuming amino acids.^201^ While amino acid depletion therapies exhibit broad applicability in cancer treatment, intriguing findings suggest that, in certain contexts, specific amino acids may possess antitumor properties.^202^ The metabolic profiling of cachectic patients revealed significant perturbations in amino acid and protein metabolism.^203^ Targeted amino acid supplementation strategies have the potential to preserve energy homeostasis and alleviate chemotherapy-induced adverse effects in cancer patients.^204^

Emerging evidence suggests that the intricate relationship between altered amino acid metabolism and pivotal tumor characteristics, including growth, metastasis, and therapeutic recalcitrance, is mediated through the orchestration of immune cell fates (Table 1). Numerous amino acids reportedly modulate immune cellular function.^202^ For example, extracellular L-arginine concentrations regulate T-cell antigen receptor ζ-chain expression, thereby influencing the normal function of T cells.^205^ Glutamine enhances GSH de novo synthesis via catabolism, providing essential precursors for glutathione biosynthesis, and inhibits the production of ROS by generating GSH and NADPH, which are required during Th17 cell differentiation to maintain redox balance and support effector function.^206^ Glutamine deprivation inhibits M2-like macrophage differentiation and CCL22 chemokine production,^207^ which may be induced by the inhibited generation and recruitment of MDSCs.^208^ Methionine provides essential methyl groups for DNA and RNA methylation, thereby promoting T-cell differentiation and proliferation by mediating mTORC1 activity.^209^

In conclusion, the metabolic reprogramming of amino acids has been characterized extensively across various tumor types, modulating infiltrating cell functions within the tumor macroenvironment. Further elucidation of the dysregulated amino acid metabolism associated with cancer cachexia could lead to the development of novel immunotherapy strategies.

##### Essential amino acids

In animals, essential amino acids (EAAs) include valine, isoleucine, leucine, phenylalanine, tryptophan, methionine, lysine, threonine, and histidine, whose carbon frameworks cannot be biosynthesized de novo by animal cells and therefore must depend on an exogenous dietary supply to sustain growth and nitrogen homeostasis.^210^ EAA/leucine supplementation in advanced lung cancer patients elicits anabolic responses, which favorably impact patient prognosis.^211^ Among EAAs, BCAA, including valine, leucine, and isoleucine, have garnered extensive attention in cachexia research. Metabolomic analyses linked BCAA degradation to sarcopenia in liver cirrhosis patients.^212^ Accelerated onset of cancer cachexia is observed in tumor-bearing mice with conditional knockout of muscle-specific branched-chain α-ketoacid dehydrogenase kinase due to increased protein ubiquitination, degradation and compromised synthesis. Importantly, supplementation with BCAAs can ameliorate these effects.^213^ Modulating the muscle BCAA transporter LAT1 has emerged as a promising therapeutic approach for cachexia.^214^

Baek et al. identified the C-terminal unique attached sequence motif domain of leucyl-tRNA synthetase as an activating domain of mTORC1, suggesting its potential as a muscle-enhancing mTOR-targeting protein.^215^ However, another study reported that a leucine-rich diet improved muscle function in cachectic tumor-bearing rats without altering mTOR activity, implying that this dietary intervention may exert beneficial effects through an mTOR-independent pathway.^216^ Furthermore, a leucine-rich diet inhibits tumor-induced cardiac injury, myocardial proteolysis, and apoptosis.^217^ Interestingly, maternal leucine nutritional supplementation improved muscle protein balance in cancer cachexia-induced muscle damage in adult offspring rats.^218^ When combined with exercise, a leucine-rich diet improves protein turnover.^219^ Similarly, β-hydroxy β-methyl butyrate, a leucine metabolite, exhibits protective properties.^220^ However, leucine supplementation in a murine model of pancreatic cancer accelerated tumor progression in both lean and overweight phenotypes, albeit with different biological outcomes.^221^ In lean mice, leucine enhances mTOR phosphorylation and subsequent S6 ribosomal protein activation, whereas in overweight mice, it disrupts glucose homeostasis by impairing clearance mechanisms, thereby increasing circulating glucose availability for tumor cell utilization.^221^ Schrems et al. reported that leucine potentially exacerbates mortality in male mice but not in female mice,^222^ and the mechanisms underlying sex-specific disparities in the impact of leucine on cancer cachexia need further study.

Elevated levels of the ketogenic amino acids phenylalanine and tryptophan were observed in cancer cachexia mouse models, suggesting their potential as biomarkers for skeletal muscle loss and reduced protein synthesis.^223^ However, mice subjected to a tryptophan-deficient diet presented a greater fiber diameter in the anterior tibialis than did those fed a standard diet. The reduction in glycolysis due to tryptophan deficiency may suppress mTOR signaling, while the increased levels of amino acids seem to compensate for the diminished pyruvate-derived energy production in skeletal muscle.^224^ Additionally, lysine was shown to serve as a diagnostic biomarker for cancer cachexia.^225^ In summary, EAAs play a multifaceted role in the pathological process of cancer cachexia (Fig. 5). Although supplementation with these amino acids can stimulate protein anabolism, it is important to recognize that certain amino acids may also exacerbate cachexia by modulating inflammation, immunity, and energy metabolism.

##### Nonessential amino acids

In most mammals (e.g., humans, rats, and pigs), the traditionally categorized nonessential amino acids (NEAAs) include alanine, arginine, asparagine, aspartic acid, cysteine, glutamic acid, glutamine, glycine, proline, serine and tyrosine, which can be synthesized through their own metabolic pathways to fulfill normal physiological needs, even if these amino acids are absent from the diet.^210^ Recent investigations underscore their crucial role in cancer cachexia (Fig. 5). For example, elevated levels of methylarginine metabolites in tumor-bearing mice inhibit protein synthesis and mitochondrial protein quality, thereby contributing to muscle wasting in cachexia.^226^ The cationic amino acid transporter, which is an essential factor for the transport of L-arginine into mitochondria, is significantly downregulated in the mitochondrial membranes of cachectic mouse muscles. These findings suggest that mitochondrial dysfunction may impact amino acid metabolism in cachectic skeletal muscles.^227^

Research indicates that gastrointestinal cancer patients receiving cysteine-supplemented parenteral nutrition exhibit reduced overall survival compared with those without cysteine supplementation. In vitro and in vivo mouse experiments confirmed that cystine increases cancer cell proliferation by activating mTORC1 via the GCN2-ATF4-SESN2 axis. Additionally, cystine confers resistance to chemotherapy by quenching chemotherapy-induced oxidative stress through GSH synthesis.^228^ However, other evidence suggests that coadministration of cystine and theanine can significantly prevent weight loss and adipose and skeletal muscle depletion. Therefore, the underlying mechanisms behind this phenomenon remain to be further investigated.^229^

Inhibition of the initial rate-limiting enzyme in serine biosynthesis drastically reduces myofibrillar protein synthesis, thereby emphasizing the crucial role of serine production in maintaining muscle size.^230^ Low serum levels of glutamine, histidine, alanine, and glycine correlate with poor survival among cancer patients, supporting their important roles in cancer progression.^231^ Supplementation with glycine^232^ and β-alanine^233^ reduces oxidative and inflammatory burdens, thereby protecting skeletal muscle from cancer-induced atrophy and functional loss. These studies demonstrate that these amino acids cannot be dismissed as unimportant or completely neglected in the human body. Moderate consumption of NEAAs is necessary for maintaining normal physiological functions. Furthermore, in specific circumstances such as cancer cachexia, the body’s requirement for NEAAs may increase, making it advantageous to moderately increase the intake of relevant foods.

##### Amino acid derivatives

Amino acid derivatives are compounds that possess novel properties and functions, arising from a series of chemical reactions involving amino acids, and play diverse and crucial roles within organisms. Importantly, multiple studies have indicated that supplementation with specific amino acid derivatives can be advantageous for alleviating the phenotypic manifestations of cachexia. Patients suffering from cachexia exhibit low basal circulatory levels of glutamine.^234^ Further studies confirmed that L-glutamine supplementation curtails tumor growth and tumor-induced cachexia.^235^ Tumor progression often elicits oxidative stress, which is linked to stromal neuropathy and intestinal atrophy and is partially attributed to depletion of GSH, the primary endogenous antioxidant. L-GSH supplementation elevates GSH levels and therefore minimizes the overexpression of nNOS and nitric oxide (NO) production in the gut, thereby preventing intestinal neurotoxicity mediated by peroxynitrite formation. This, in turn, helps to preserve the ileal neuronal count and morphology, alleviating cachexia-related gastrointestinal motility impairments.^236^

Taurine, a cysteine derivative, protects myoblasts against cisplatin-mediated cytotoxicity by enhancing ROS scavenging and GSH synthesis. Moreover, it regulates myotube differentiation by modulating the expression of myogenic markers and attenuates myotube atrophy.^237^

Creatine, an amino acid derivative synthesized from L-arginine, glycine, and L-methionine, has been extensively investigated in the context of tumor progression. Creatine supplementation effectively prevents tumor-induced increases in plasma homocysteine levels and hepatic oxidative stress, thereby alleviating tumor growth and weight loss.^238^ Compared with tumor-bearing rats, creatine intake diminishes plasma TNF-α and IL-6 levels and alleviates splenic morphological alterations, such as reduced white pulp and lymph follicle sizes, indicating that creatine may prevent skeletal muscle atrophy by modulating the tumor-induced proinflammatory milieu.^239^

L-carnitine, a derivative of lysine, has been shown to alleviate fatigue, stimulate appetite, and increase lean body mass in advanced cancer patients following supplementation.^240^ L-carnitine can induce the restoration of hepatic lipid metabolic dysfunction, accompanied by a reduction in the circulating concentrations of TNF-α and IL-6.^241,242^

Generally, amino acid metabolites, including glutamine, GHS, taurine, creatine, and L-carnitine, mitigate cachexia phenotypes by mitigating inflammatory reactions, alleviating oxidative stress, increasing energy expenditure, and modulating metabolism.

#### Fatty acid metabolism

Increasing evidence suggests that fatty acids play crucial roles in cancer cachexia via diverse mechanisms, including energy provision, metabolic modulation, anti-inflammatory and immune regulation and intestinal barrier maintenance (Fig. 5). According to their carbon chain length, fatty acids are classified into short-chain (SCFAs, <6 carbons), mid-chain (MCFAs, 6–12 carbons) and long-chain (LCFAs, >12 carbons) fatty acids. Furthermore, fatty acids can be dichotomized on the basis of saturation status into saturated (SFAs) and unsaturated (UFAs) fatty acids, with UFAs further subdivided into monounsaturated (MUFAs) and polyunsaturated (PUFAs).^243^ PUFAs are categorized into Omega (ω)-3 and ω-6 families on the basis of the location of the first double bond from the methyl end. ω-3 PUFAs include alpha-linolenic acid (ALA), eicosapentaenoic acid (EPA), and docosahexaenoic acid (DHA), whereas the ω-6 series includes linoleic acid (LA), gamma-linolenic acid, conjugated linoleic acid, and arachidonic acid.^243^ Elucidating their potential roles in cancer cachexia may promote the development of enhanced nutritional and metabolic interventions.

##### Long-chain fatty acids

During the occurrence and progression of cancer cachexia, alterations in long-chain fatty acid composition, both intracellularly and in plasma, may occur. An investigation utilizing a rodent model of cancer cachexia revealed increased palmitic acid and decreased LA levels in WAT, associated with downregulated expression of ELOVL6, a gene encoding an enzyme crucial for fatty acid elongation. These findings suggest that the modulation of adipocyte lipid composition and content may inhibit excessive lipogenesis.^244^ The level of oleic acid, a type of MUFA, was significantly greater in the plasma of PDAC patients with cachexia, potentially reflecting compensatory mechanisms in response to malnutrition or impaired tissue uptake of oleic acid. Furthermore, LA, an ω-6 PUFA, is positively correlated with albumin and hemoglobin levels in cachexia patients, suggesting potential nutritional support from increased dietary LA.^245^ However, Wang et al. reported elevated LA levels and decreased LA-rich cardiolipins in the hearts of cancer cachexia patients. The degradation of LA-rich cardiolipins may underlie the increased LA levels, which are implicated in inflammation, apoptosis, and atrophy of heart tissues in cancer cachexia. This finding aligns with an elevated ω-6/ω-3 PUFA ratio in heart tissues and indicates that targeting disturbances in glycerophospholipid and fatty acid metabolism in cancer cachexia hearts may be helpful.^246^ Therefore, further research is needed to identify the role of ω-6 PUFAs in cancer cachexia.

The role of ω-3 PUFAs in cachexia has been extensively reported. Among these, EPA and DHA are the most studied because of their potent anti-inflammatory and antioxidant properties.^247^ The plasma level of ω-3 fatty acids is decreased during chemotherapy for lung cancer patients, which may exacerbate muscle wasting.^248^ Additionally, a meta-analysis suggested that low-dose ω-3 FA supplementation stabilizes weight, appetite, and quality of life in cancer patients, especially those with tumors of the upper digestive tract and pancreas.^249^ However, fish oil therapy enriched with ω-3 FAs could have potential gastrointestinal side effects such as nausea, emesis, and diarrhea.^250^ Low-dose marine phospholipid supplementation offers comparable benefits to those of fish oil, with better tolerability and fewer side effects, effectively improving weight and appetite in cancer patients.^251^

In tumor-bearing mice, EPA antagonizes the loss of skeletal muscle proteins in cancer cachexia by suppressing ATP-dependent proteolysis and the expression of 20S proteasome alpha-subunits and the p42 regulator,^252^ which can also prevent NF-κB nuclear accumulation, thereby dampening proteolysis-inducing factor-triggered ubiquitin‒proteasome proteolysis.^253^ DHA-phospholipid and EPA-phospholipid act against TNF-α-triggered lipolysis in adipocytes.^254^ The combination of free fatty acid receptor FFA1/FFA4 agonists (GW9508 and TUG891) with ALA/DHA reduces tumor weight in LLC mice. Furthermore, these findings suggest that ALA and DHA may exert their therapeutic effects on cancer cachexia, at least in part, through the activation of FFA1/FFA4 receptors.^255^ Collectively, ω-6 and ω-3 PUFAs appear to exert opposing effects on cachexia, underscoring the need for further investigation into their mechanistic interplay and the identification of optimal ω-6/ω-3 ratios for therapeutic benefit in cachexia patients.

Accumulating evidence highlights the intricate link between PUFAs and the function of immune cells, which may provide a possible explanation for the anti-inflammatory changes observed after exogenous administration of PUFAs during the progression of cancer cachexia (Table 1). ω-3 PUFAs enhance anti-inflammatory responses by augmenting IL-10 release from macrophages, fostering Treg induction and inhibiting excessive Th17 cell differentiation. They also reduce oxidative stress and NF-*κ*B-mediated inflammation in immune cells.^256^ GPR120/FFA4, a G protein-coupled receptor for long-chain fatty acids, is potently activated by ALA.^257^ Once FFA4 is activated by ALAs, macrophages are polarized toward the M2 phenotype to modulate inflammation in adipose tissues. For example, FFA4 activation reduces TNF-α and IL-6 secretion in mouse macrophages.^257^ ω-3 PUFAs also modulate NK cell function. DHA inhibits tumor growth by increasing peroxisome proliferator-activated receptor-gamma coactivator (PGC)-1α-mediated mitochondrial oxidative phosphorylation activity in NK cells and increasing IFN-γ production.^258^ However, in certain contexts, PUFAs may exert immunosuppressive effects. Tumor-derived alpha-fetoprotein binds more PUFAs than does umbilical blood-derived alpha-fetoprotein, which induces a glycolytic phenotype in dendritic cells. Therefore, ω-3 PUFAs enhance glucose uptake and lactate secretion, leading to suppression of the antitumor capabilities of dendritic cells.^259^ Apart from immune cells, ω-3 PUFAs are crucial in regulating stromal cell functions (Table 1). Interstitial fluid pressure, a significant barrier to anticancer drug diffusion, is elevated by NO-mediated tumor angiogenesis. ω-3 PUFAs diminish endothelial NO synthase activation, thereby lowering interstitial fluid pressure and enhancing anticancer drug delivery efficiency.^260^

LCFAs, particularly ω-3 PUFAs, have been extensively investigated in cancer cachexia, presumably because of their potential immunomodulatory and redox-regulatory capacities. Notably, several studies have indicated their crucial roles in diverse immune and stromal cell functions, warranting further research in the cachexia field.

##### Mid-chain fatty acids

MCFAs include both even-carbon MCFAs (including hexanoic acid, octanoic acid, and decanoic acid and, although controversial, lauric acid due to its twelve carbons) and odd-carbon MCFAs (such as heptanoic acid, nonanoic acid, and undecanoic acid). MCFAs, notably lauric acid, are rapidly assimilated into mitochondria, stimulating oxidative phosphorylation and resulting in the apoptosis of tumor cells. This effect is tumor specific, sparing skeletal muscle and other nontumor cells. The consumption of lauric acid elevates skeletal muscle protein levels and halts tumor growth through a tumor-selective oxidative stress response, mitigating cancer-induced skeletal muscle atrophy.^261^ Furthermore, dietary intervention combining lauric acid and glucose for cancer cachexia may ameliorate cancer-associated myocardial damage by increasing mitochondrial respiration and ATP synthesis without exacerbating oxidative stress.^157^ A study indicated that caprylic acid has a greater capacity than capric acid and lauric acid for improving mitochondrial quality and promoting skeletal muscle maturation. These findings support the therapeutic role of caprylic acid in cancer cachexia.^262^ Diets with medium-chain triglycerides consisting of glycerine and MCFAs also offer an optimal ketogenic regimen for reversing weight loss in cancer cachexia patients, accompanied by a reduction in tumor size.^263^ In the immune microenvironment, the dysregulation of MCFA may affect the functional modulation of immune effector cells. For example, endogenous MCFAs can activate GPR84 to enhance inflammatory responses and macrophage phagocytosis.^264^ Disorders of MCFAs in cancer cachexia may disrupt macrophage function.

##### Short-chain fatty acids

SCFAs are metabolic byproducts generated via microbial fermentation of complex carbohydrates in the gut, which emerge as pivotal players in cancer cachexia, especially butyrate and acetate.^80,82^ The decrease in butyrate and acetate is closely related to the reduction in the abundance of members of the *Ruminococcaceae* and *Lachnospiraceae* families in cachectic cancer mice, thereby exacerbating intestinal inflammation through the impairment of intestinal barrier integrity.^82^ Mechanistically, butyrate not only minimizes endotoxin translocation and oxidative stress but also promotes polarization toward the M2 phenotype, inhibiting inflammation and macrophage infiltration into muscles. Furthermore, it counteracts cachexia-induced muscle atrophy by modulating pathways such as the Akt/mTOR/Foxo3a and Fbox32/Trim63 pathways.^265^ Microbiome-derived SCFAs can induce IL-22 production in CD4^+^ T cells and innate lymphoid cells by enhancing HIF-1α binding to the *Il-22* promoter via histone modification, thereby maintaining intestinal homeostasis.^266^

In addition to preserving intestinal homeostasis, fatty acids play critical roles in orchestrating host immune responses and modulating systemic inflammation, thereby having the potential to attenuate cachexia progression. It is necessary to further understand their importance, and individualized treatments for patients should be developed.

#### Other metabolites

Nevertheless, numerous other metabolites are associated with the progression of cachexia. For example, vitamin D supplementation effectively ameliorates the disruption of UCP1 and ATP levels in adipose tissue and muscle. It modulates biomarkers of beige adipocytes and browning and normalizes the levels of the entire Tlr/NF-κB pathway in inguinal WAT. It also inhibits muscle catabolic signaling and enhances muscle regeneration and the myogenesis process.^267^ Furthermore, supplementation with 25(OH)D₃ may confer more pronounced improvements in muscle atrophy and adipose browning in cachectic mice than supplementation with 1,25(OH)₂D₃.^268^

Decreased ATP concentrations and total adenine nucleotide pools represent frequent features of atrophic muscles in human diseases. In cancer cachexia, energy metabolism is frequently disrupted by tumor proliferation and increased catabolism, resulting in reduced ATP and ADP levels in muscle tissue that normalize after tumor resection.^269^ Xanthine oxidase inhibitors hold promise as potential therapeutic agents to mitigate muscle wasting and cardiac dysfunction in cancer cachexia.^270^ In lung cancer patients, upregulated AMPK contributes to alleviating cancer-induced metabolic perturbations by preventing glucose intolerance and insulin resistance and decreasing glucose disposal in skeletal muscle and WAT. The AMPK-dependent upregulation of PDKs may represent an adaptive metabolic reprogramming strategy to maintain essential metabolic functions within the muscle, emphasizing the metabolic underpinnings of cachexia.^271^

Cells generate several active byproducts of metabolic pathways, such as ROS, NAD + /NADH, and NADP + /NADPH. The concurrent impairment of NAD^+^ and downregulation of the NAD^+^ biosynthetic enzyme *nicotinamide riboside kinase 2* were identified as common features of severe cachexia in both preclinical murine models and cancer patients. The supplementation of vitamin B3 niacin enhances NAD^+^ levels and increases mitochondrial biogenesis to ameliorate muscle mass loss and metabolic disorders.^272^ Preemptive intake of nicotinamide riboside, a substrate for NAD^+^, significantly reduces the levels of the cachexia-inducing cytokines TNF-α and IL-6 in a mouse model, decreases muscle-specific ubiquitin‒proteasome ligase activity, and inhibits lipolysis.^273^

Oxidative stress, intricately linked to cachexia, results from an imbalance between oxidation and antioxidation. Oxidative stress generates excessive ROS, such as superoxide anions, hydrogen peroxide, and hydroxyl radicals, which are harmful to cellular proteins, DNA, and lipids.^274^ Elevated oxidative stress in skeletal muscle disrupts mitochondrial function, energy metabolism and ATP production, leading to mitochondrial autophagy-related muscle damage.^275^ This mitochondrial dysfunction, in turn, leads to increased oxidative stress. Furthermore, oxidative stress can activate the UPS^276^ and calpains^277^ in skeletal muscle to accelerate protein degradation and modulate Akt phosphorylation to affect protein synthesis, such as the phosphoinositide 3-kinase/protein kinase B (PI3K/Akt) pathway,^278^ ultimately exacerbating muscle atrophy.

In conclusion, these metabolites are closely associated with cancer cachexia. These aberrant alterations not only reflect tumor metabolic signatures but also impact host cell function via intricate metabolic networks and signaling cascades, thereby intensifying catabolic processes and cachexia manifestations.

### Regulatory effects of immune and stromal cells on systemic metabolism

These profound and aberrant metabolic transformations are coupled with the progression of cancer cachexia. Importantly, these metabolic alterations strongly impact the internal architecture, functional properties, and dynamic quantity of immune cells and stromal cells. As a result, immune cells such as TAMs, tumor-associated neutrophils (TANs), tumor-infiltrating T lymphocytes (TILs) and MDSCs can modulate cancer cachexia via the following mechanisms: (1) they impact key target organs such as skeletal muscle, adipose tissue and even the nervous system, inducing a cascade of pathological effects within these tissues through signal transduction mechanisms; and (2) they exert a broader influence by secreting a range of bioactive molecules, termed “cachectic factors”, which further propagate their effects (Fig. 6). Upon dissemination through the circulatory system, cachectic factors act as potent signaling molecules, promoting cachexia progression across tissues and organs. By binding to receptors on target cells, they elicit downstream signaling cascades that augment inflammation, accelerate tissue catabolism, and inhibit anabolic processes, thereby exacerbating cachexia. The reconfiguration of stromal cellular phenotypes plays a significant role. CAFs can produce cytokines, secrete extracellular vesicles, and synthesize ECM, all of which influence tumor progression. The destruction of ECs leads to microvascular circulation disorders in various tissues, such as skeletal muscle and adipose tissue, ultimately resulting in malnutrition (Fig. 6). In summary, these cellular alterations exacerbate clinical symptoms such as weight loss, muscle atrophy and fat depletion, severely compromising immune defenses and posing formidable obstacles to disease management and prognosis.

#### Tumor-associated macrophages

TAMs constitute up to 50% of the TME in certain solid tumors and play a pivotal role in immune cell infiltration. Upon receiving signals from the TME, macrophages are differentiated into either proinflammatory or anti-inflammatory phenotypes on the basis of environmental cues. Although macrophages can be classified into two subsets, i.e., M1-like macrophages or M2-like macrophages, they are collectively designated TAMs.^279^ Accumulating evidence underscores the critical role of TAMs in mediating cancer cachexia (Fig. 6). In pancreatic cancer mouse models, macrophage depletion restores muscle and epididymal fat weight, accompanied by increased grip strength and mobility.^280^

Macrophages are major immune cells in adipose tissue. They support the nutrient storage function of adipocytes in the absence of metabolic dysfunction and then release inflammatory factors to promote insulin resistance during the progression of metabolic syndrome.^281^ The activation of beige adipose thermogenesis is orchestrated by both the SNS and adipose tissue macrophages (ATMs), but a detailed mechanism for this process is lacking. In BAT, the minimal fundamental thermogenic unit is the brown adipocyte, with norepinephrine serving as the paramount regulatory factor.^87^ Ajay’s group reported that cold stimuli can recruit macrophages to adipose tissue regions in a CCR2-dependent manner. Moreover, eosinophils induce catecholamine production in macrophages via the IL-4/13-IL-4Rα-STAT6 signaling axis, triggering thermogenic gene expression in BAT and promoting lipolysis in WAT.^282,283^ Furthermore, in a cancer cachexia mouse model, researchers reported an increase in the number of IL-4-induced M2 macrophages, which express neurotrophic factors that promote a neuroprotective milieu, within the inguinal WAT. These factors facilitate catecholamine production by peripheral sympathetic nerves and then orchestrate lipid metabolism, WAT browning, and adipose tissue atrophy in cancer cachexia. These findings highlight the potential role of ATM-SNS crosstalk in cancer cachexia.^156^ However, another study reported that macrophages significantly promote beige adipose thermogenesis independently of the SNS after inducing sympathetic neuronal apoptosis with 6-hydroxydopamine in mice.^284^ M2 macrophage-derived TGF-β induces senescence in adipose progenitor cells and inhibits adipogenesis in aged mice. However, the role of this mechanism in cancer cachexia remains unclear and warrants further investigation.^285^ In the fibrotic regions of subcutaneous adipose tissue from gastrointestinal tumor patients, infiltrated CD68^+^ macrophages and CD3-Ly have been identified, suggesting their potential roles in cachexia-related adipose tissue remodeling.^286^ Inactivation of hypoxia-inducible factor 1α in myeloid cells severely impairs energy generation, leading to robustly compromised cellular function and defective myeloid cell-mediated inflammation. However, reduced levels of proinflammatory cytokines unexpectedly increase cachexia-associated fat loss coupled with reduced ATMs. This finding suggests that ATMs may play a protective role in cancer cachexia-related adipose loss.^287^

In addition to facilitating adipose tissue catabolism, macrophages influence skeletal muscle primarily through the secretion of inflammatory cytokines. For example, the coculture of macrophages and tumor cells accelerates myotube atrophy via macrophage-derived IL-6 and IL-1α.^288^ Another experiment further solidified the intimate connection between macrophages and tumor cells. An increased percentage of CD68^+^ macrophages was observed in tumor tissues from cachectic patients compared with noncachectic individuals. Notably, the transcriptional upregulation of ZXDC in tumor cells facilitates macrophage recruitment through the CCL2/CCR2 signaling axis. These macrophages subsequently secrete CCL5, which activates the NF-κB signaling cascade, leading to the upregulation of TWEAK in tumor cells.^289^ Elevated TWEAK subsequently initiates muscle remodeling and promotes muscle atrophy via MuRF1 activation.^289,290^ However, macrophages can also facilitate muscle recovery and regeneration via insulin-like growth factor 1 (IGF-1) autocrine signaling, which shifts polarization toward the M2 phenotype.^291^ Recent research by Pryce et al. may explain the contrary role of macrophages in cachexia. They identified distinct cellular subtypes of macrophages that are induced by NF-κB. Proinflammatory macrophages promote atrophy by inhibiting muscle regeneration, whereas anti-inflammatory macrophages, to some extent, contribute to maintaining myofiber size.^292^

#### Tumor-associated neutrophils

Neutrophils, some of the most abundant immune cells in the human circulation, perform pivotal functions in combating inflammation caused by infections and injuries. Accumulating evidence indicates that neutrophils also participate in various aspects of tumor biology, including metastasis, drug resistance, and poor prognosis.^279^ Compared with noncachectic patients, cachectic patients exhibit a marked elevation in absolute neutrophil count coupled with an elevated neutrophil‒lymphocyte ratio (NLR),^51,293–296^ accompanied by the upregulation of angiotensin II and neutrophil-derived proteases.^293^ Currently, several studies have reported the important role of neutrophil-related indicators in evaluating inflammatory status, such as the NLR-to-handgrip strength ratio,^294^ the neutrophil-to-albumin ratio,^296^ and the advanced lung cancer inflammation index, which is calculated as BMI (kg/m^2^) × albumin (g/dl)/NLR.^295^ Additionally, the modified advanced lung cancer inflammation index, which is defined as the appendicular skeletal muscle index × serum albumin/NLR, was also introduced.^297^ These indices were confirmed to be negatively correlated with survival rates in cancer cachexia patients, suggesting the prognostic significance of neutrophil-mediated processes in cancer cachexia patients (Fig. 6).

As tumors proliferate, tissues gradually undergo damage, necrosis, and hypoxia, triggering the release of numerous chemokines, such as TNF-α, which facilitate the recruitment and activation of neutrophils and macrophages.^298^ During the precachexia stage, neutrophils are elevated in tissues such as the liver and lungs and increase in number in the circulatory system.^299^ Upon recruitment, activated neutrophils unleash neutrophil-derived proteases and angiotensin II. Elevated plasma levels of angiotensin II are associated with increased production of inflammatory cytokines such as IL-6, IL-8, and TGF-β1, which contribute to the inflammatory state and cause the activation of proteolytic pathways and/or disruption of protein synthesis, thus promoting cancer cachexia.^293^ Recently, researchers defined a subset of neutrophil-like monocytes, termed cachexia-inducible monocytes, which express CD38^+^ and induce muscle atrophy by producing IL-36G, thereby exacerbating cachexia phenotypes in advanced cancer models.^300^

In murine models of cancer cachexia, neutrophils are increased in various tissues and secrete cytotoxic proteins such as LCNs, which can suppress appetite or promote ferroptosis-related inflammation.^67,301^ Anorexia, a common symptom in cachexia patients, is driven by cerebral inflammation. Studies have revealed that neutrophils can be recruited to the brain through TRIF-dependent mechanisms.^73^ Afterward, neutrophils accumulate in a CCR2-rich meningeal region of the velum interpositum, which is adjacent to the hippocampus and habenula areas that are crucial for appetite regulation. Here, neutrophils contribute to anorexia and muscle catabolism through neuroimmune circuits.^75^

Neutrophils contribute to skeletal muscle atrophy and anorexia nervosa through the release of substantial amounts of inflammatory cytokines and cytotoxic proteins, highlighting the importance of preventing their detrimental effects on cancer cachexia.

#### Tumor-infiltrating T lymphocytes

Emerging evidence underscores the pivotal role of adaptive immune cells, particularly T cells, in the pathology of cachexia. Studies have revealed a positive relationship between muscle strength and the frequencies of CD8^+^ naïve T cells, CD8^+^ effector T cells, and CD197^+^CD45RA^+^ Tregs. Conversely, CD8^+^ memory T cells and CD95^+^CD8^+^ T cells inversely correlate with muscle function, suggesting that the infusion of specific T-cell subsets may restore a balanced T-cell profile, thereby mitigating muscle atrophy and alleviating cachexia in cancer-bearing mice.^302^ Nevertheless, the intricate nature of the immune system implies that multiple factors concurrently influence muscle function and mass, resulting in inconsistencies in research findings and thereby obscuring the precise role of T cells in cachexia (Fig. 6). The thymus and spleen, which are crucial for T-cell progenitor generation and differentiation, exhibit atrophy and quantitative reductions in Tlymphocyte populations in cachexia-bearing mice. Notably, upregulated expression of immune checkpoint markers, such as cytotoxic T-lymphocyte-associated antigen 4 (CTLA-4) and PD-1, was found within CD4^+^ T cells, highlighting the involvement of immunodeficiency in cancer cachexia.^303^ The engineered rimiducid-inducible MyD88- and CD40-driven chimeric antigen receptor (CAR)-T cells elicited antitumor responses, but these CAR-T cells also caused cachexia in mice through dose-dependent weight loss. The authors further demonstrated that CD4^+^ T helper cells are the primary source of cachexia-related proinflammatory cytokines such as TNF-α. Conversely, the purification of CD8^+^ T cells enhances antitumor efficacy while minimizing cytokine-related toxicity.^304^ However, other studies have shown that CD4^+^ T cells play a protective role in cachexia. For example, cachectic mice display notable depletion of CD44^low^ CD4^+^ T cells, a type of quiescent naïve cell, in lymph nodes and spleens compared with noncachectic diabetic tumor-bearing mice. Intriguingly, infusion of these CD44^low^ CD4 + T cells attenuated body weight reduction and preserved skeletal muscle mass in tumor-bearing mice.^305^

The role of CD8^+^ T cells in cachexia is equally complex. A study demonstrated that the transcriptional signature of CD8^+^ T cells is negatively correlated with genes involved in muscle wasting pathways, the ubiquitin proteasome, and apoptosis/autophagy, suggesting a potential protective role of CD8^+^ T cells in cachexia.^306^ In a mouse model of PDAC, a TLR7/8 agonist showed an antitumor response and improved cachexia by increasing CD8^+^ T-cell infiltration and decreasing the number of Tregs.^307^ Interestingly, CD8^+^ T cells trigger cachexia through antigen-specific activation in a reversible cachexia mouse model induced by chronic viral infection.^308^ Mechanistically, CD8^+^ T cells modulate adipose tissue lipid metabolism in a type I IFN-dependent manner but not the levels of the cytokines IL-6, TNF, and IFN-γ, which often mediate cancer cachexia.^308^ These findings suggest that the mechanisms causing cachexia may differ among various diseases, suggesting that when studying and treating cachexia, we need to consider the specific pathological background and avoid generalization.

Notably, the abnormal metabolism associated with cancer cachexia can reciprocally influence the function of immune cells. In advanced lung patients, cachexia status inversely correlates with immunotherapy response. Mechanistically, nutrient intake and energy utilization are largely impaired by chronic inflammation, metabolic disturbances, anemia, and anorexia, which result in defective T-cell activation and exhaustion, thereby hindering antitumour activity and immunotherapy responsiveness.^309^ These findings further support the bidirectional crosstalk between metabolic and inflammatory regulation in the cancer cachexia process.

#### Myeloid-derived suppressor cells

MDSCs, which originate from bone marrow progenitors and immature myeloid cells, possess the ability to differentiate along myeloid lineages, including dendritic cells, macrophages and granulocytes, thereby exhibiting a remarkable capacity to suppress immune cell responses, thereby accelerating the progression of cachexia (Fig. 6). During the development of cancer cachexia, a marked increase in MDSC counts is observed in the circulation of cachectic cancer patients, indicating the importance of MDSCs in cancer cachexia.^310^ Chronic inflammation mediated by IL-17 may contribute to immunosuppression or cachexia by promoting MDSC infiltration in cancer patients.^311^ Synergistic blockade of MEK and PI3K signaling reduces splenic MDSC counts, effectively alleviating weight loss and skeletal muscle atrophy.^312^ In 4T1-bearing animals, MDSC expansion increases susceptibility to inflammation-induced organ injury and death due to increased energy expenditure, adipose tissue depletion, and increased expression of acute-phase hepatic proteins.^313^

Although MDSCs alone may not be sufficient to induce cachexia, they can exaggerate cancer cachexia by suppressing adaptive immunity. For example, MDSCs inhibit T-cell activation and function, allowing tumor cells to release cachexia-related factors.^314^ A study demonstrated that partial inhibition of PARP-1 is sufficient to block the suppressive activity of MDSCs, such as impairing their ability to inhibit T-cell proliferation. Therefore, this treatment synergizes with anti-PD-1 immunotherapy and other immune checkpoint inhibitors to enhance CD8^+^ T-cell infiltration, offering protection against cachexia in a colon cancer mouse model.^315^

MDSCs frequently represent a pivotal source of cachexia-associated factors that regulate cachexia. In cachectic mice, Ly6G^+^CD244^+^ polymorphonuclear MDSCs can secrete activin A in skeletal and cardiac muscles, leading to muscle atrophy via the upregulation of E3 ubiquitin ligases.^316^ Notably, therapeutic intervention with anti-Ly6G antibodies targeting MDSCs and neutrophils reduces weight loss and muscular atrophy in both skeletal and cardiac tissues.^316,317^

However, another study reported that increased infiltration of CD11b^+^Ly6g^+^ cells is observed in skeletal muscle and adipose tissue in a colon cancer mouse model. When 5-FU chemotherapy reduces this infiltration, cachectic phenotypes are not alleviated, indicating that MDSC cells may not be the primary driver of tumor- and chemotherapy-induced cachexia.^318^ Similarly, in a preclinical cancer cachexia model, soluble ACVR2B can alleviate the cachectic phenotype by enhancing hepatic protein synthesis and reducing splenomegaly. However, the levels of circulating inflammatory cytokines and MDSCs in the spleen remain elevated. Thus, the changes in MDSCs do not explain the cachectic phenotypes in these contexts.^319^

#### Cancer-associated fibroblasts

CAFs are prevalent in various tumor tissues and exert detrimental effects by promoting tumor growth, angiogenesis, and metastasis. Recent studies increasingly implicate the role of CAFs in cancer cachexia (Fig. 6). Hypoxia within cachectic tumor regions activates the TGF-β signaling cascade via noncanonical routes such as MAPK signaling to trigger fibroblast-to-myofibroblast transdifferentiation. This transition increases the levels of ECM components and inflammatory and angiogenic factors, thereby modulating tumor aggressiveness.^320^

CAFs can also secrete various proinflammatory factors to modulate cachectic phenotypes by influencing ECM components within the TME. For example, in lung cancer, CAFs secrete IL-6 via the circNOX4/miR-329-5p/FAP axis.^321^ In breast cancer, infiltrating TAMs secrete CCL18 to induce the production of IL-6 and IL-8 by breast fibroblasts through the NF-κB signaling pathway.^322^ A prior study indicated that CAFs can secrete GDF15, supporting the role of CAFs in regulating cachectic phenotypes.^323^

Fibroblast activation protein (FAP), also known as F19, a serine protease involved in ECM remodeling, is highly expressed in CAFs but virtually absent in normal fibroblasts or other normal tissues.^324^ Moreover, FAPα^+^ fibroblasts are implicated in immune suppression. For example, FAPα^+^ fibroblasts impair the responsiveness to the T-cell-activating immune checkpoint inhibitors α-CTLA-4 and α-PD-L1 via the secretion of CXCL12.^325^ Moreover, the secretion of CCL2 by FAP+ fibroblasts promotes the recruitment of MDSCs,^326^ indicating the importance of FAP in modulating the deleterious biological behaviors of CAFs.^327^ However, recent studies have suggested that inhibiting FAP-positive CAFs can induce cancer cachexia. Tran et al. reported that the adoptive transfer of T cells genetically engineered with FAP-reactive CARs into mice with subcutaneous tumors markedly induced cachexia and bone marrow toxicity. Further investigation revealed that FAP5-CAR-transduced T cells might target FAP-positive multipotent bone marrow stromal cells, which could explain the observed bone marrow toxicity.^328^ Similarly, Roberts et al. reported that the depletion of FAP^+^ stromal cells results in muscle wasting and altered hematopoiesis.^329^ Collectively, these observations implicate fibroblasts as critical regulators of muscle homeostasis, and the absence of fibroblasts in skeletal muscle can contribute to muscle atrophy in cancer cachexia.

#### Tumor-associated endothelium

ECs, which constitute the luminal layer of nascent blood vessels, play critical roles in maintaining vascular integrity and function across diverse tissues, including the tumor stroma, skeletal muscles, and CNS. Within the TME, these vessels serve as nutrient suppliers for the tumor and therefore regulate tumor progression and metastatic potential via intricate vascular endocrine signaling mechanisms.^330^ Elevated VEGF levels and increased CD34^+^ microvessel density are widely observed in cancer patients and indicate neoangiogenesis during the progression of malignant tumors.^331^ Additionally, the matricellular protein SPARC, a novel tumor-derived vascular permeability factor, disrupts the endothelial barrier through vascular cell adhesion molecule 1 and p38 MAPK-mediated signaling, thereby promoting the metastatic competence of tumor cells.^332^ The high expression of the oncoprotein BRAF^V600E^ in undifferentiated thyroid carcinoma tumor cells promotes in vitro angiogenesis and facilitates the secretion of factors such as VEGFA, VEGFC, and IL-6 by tumor cells, which contributes to cachexia and metabolic alterations in advanced thyroid cancer.^333^ Similarly, the delicate balance between anabolic protein synthesis and catabolic proteolytic breakdown of skeletal muscle partly depends on the integrity of the blood microcirculation. Healthy skeletal muscles are abundantly supplied with capillaries. When ECs are dysfunctional, the nutrient supply within muscles is impaired. Therefore, protein synthesis is reduced, impairing muscle regeneration and leading to fibrosis and compromised contractility.^334^ In KPC mice with cachexia, muscle tissue is characterized by increased capillary leakage and infiltration of immune cells and inflammatory factors such as IL-1β, IL-10, and IL-6. Notably, Activin-A inhibits PGC1α expression in ECs within muscle tissue, which disrupts vascular integrity, leading to vascular leakage and muscle loss.^335^ This finding is consistent with studies showing that PGC1α overexpression in myocytes can partially inhibit age-related muscle atrophy by activating mitochondrial oxidative metabolism and neovascularization.^336^

Moreover, the BBB serves as a crucial interface for communication between the CNS and peripheral tissues, facilitating the transport and interaction of appetite-regulating cytokines with brain ECs and thereby modulating the release of appetite-altering substances into the brain interstitial fluid.^337^ Endothelial peroxynitrite generation by cisplatin mediates neurotoxicity through neuronal caspase-1, which participates in abnormal neuronal γ oscillations in the hypothalamic arcuate nucleus, causing decreased food intake and weight loss in mice.^338^ ECs play pivotal roles not only in angiogenesis but also in regulating immune cell infiltration and inflammatory responses (Fig. 6). P-selectin, which is expressed on platelets and ECs, facilitates leukocyte recruitment by binding with PSGL-1, enabling leukocyte capture and rolling on stimulated ECs. This mechanism increases the aggregation of leukocytes and activated platelets and the secretion of inflammatory cytokines, which promote the manifestation of cachectic phenotypes.^339^ TNF-α and IL-1β can induce the expression of the notch ligand JAG1, and tumor-derived exosomes may also transfer DLL4 to activate notch receptors on distant ECs. This activation increases the synthesis of retinoic acid and IL-33 in ECs. Secreted IL-33, in turn, stimulates excessive production of retinoic acid in adjacent adipocytes and macrophages, which contributes to maintaining catecholamine-induced WAT reduction via increased UCP1 levels. Additionally, retinoic acid enhances the expression of IGFBP3, which promotes antiadipogenesis and apoptosis, thereby influencing lipid storage and adipocyte turnover.^340^ Tumor ECs release chemerin in response to chemotherapy, which can increase NK cell recruitment and alleviate WAT lipolysis and proteolytic effects on skeletal muscle. Targeting VEGF-A in myeloid cells can promote the release of chemerin and ameliorate cachexia.^341^

ECs are critical cellular components that form the vascular lumen, and their structural integrity and functional status are essential for nutrient delivery to peripheral tissues. Their destruction not only results in energy deficiency but also disrupts the blood vessel barrier, potentially promoting tumor cell metastasis, immune cell mislocalization, and the accumulation of inflammatory factors, ultimately accelerating cachexia progression.

#### Regulatory roles of other cell types in the TME

The development of cachexia is intricately intertwined with various cellular components and their secreted cytokines. It is well documented that distinct types of cells within the TME, including TAMs, TANs, TILs, MDSCs, CAFs, and ECs, modulate metabolic and immunologic responses. However, other cellular components within the TME should not be overlooked, as they may also have potential roles in cachexia. For example, in a lung cancer mouse model of cachexia, a significant increase in activated and degranulated mast cells was observed in skeletal muscle. Consistently, exposure of C2C12 myotubes to conditioned media from tumor-activated mast cells results in a marked reduction in myotube diameter, suggesting that tumor-activated mast cells secrete certain factors to mediate skeletal muscle atrophy.^342^ NK cells may alleviate cachexia by eradicating senescent tumor cells,^341^ and they may also contribute to cachexia pathogenesis in specific mouse models. For example, upregulated NK cell activity was identified in a peritonitis-induced sepsis model.^343^ Furthermore, NK cells swiftly activate the immune system by producing cytokines such as IFN-γ, TNF-α, IL-10, GM-CSF, and chemokines, which are canonical cachexia-associated factors.^344^ While the direct contribution of B lymphocytes to cachexia is less understood, the antibodies produced by B lymphocytes can modulate cancer cachexia progression by regulating immune responses and metabolic states.

In conclusion, the onset and progression of cachexia are intricately governed by diverse cellular components. Elucidating the intricate interplay between cachexia and cells within the TME is paramount for understanding its pathogenesis and identifying effective therapeutic interventions.

### Cachexia-associated inflammatory cytokines regulate metabolic reprogramming

As mentioned earlier, cancer cachexia is often accompanied by dysregulation of inflammation regulation. Therefore, in the past few decades, various studies have focused extensively on the role of proinflammatory cytokines in cachexia syndrome. A series of factors, including members of the interleukin family (IL-1, IL-4, IL-6, and IL-20), interferon (IFN-γ), TNF-α, and growth factors such as TGF-β, oncostatin M (OSM), leukemia inhibitory factor (LIF) and GDF15, as well as chemokines (LCN2), were identified. All of these factors are significantly elevated in cachexia patients.^345^ Metabolic abnormalities in various organs or tissues, such as the gut, muscles, and fat, can lead to elevated levels of cytokines. Prolonged high levels of inflammatory factors can further disrupt muscle and fat metabolism, exacerbating cachexia. However, numerous studies have indicated that anti-inflammatory treatments targeting these factors have not yielded satisfactory results, suggesting that a deep understanding of the pathology of cancer cachexia is needed. Recently, several new cachexia-related factors, such as the growth factor GDF15 and the chemokine LCN2, which participate in cachexia through mechanisms that amplify inflammatory signals beyond traditional pathways, have been reported (Table 2).

**Table 2:** Regulatory roles of inflammatory factors in systemic metabolic reprogramming

Classification	Mediators	Receptor	Major Activities	Mechanism of Action	References
Interleukin family	IL1	IL1R1, IL1R2	Inhibit food intake; promote protein and fat decomposition; enhance IL6 expression	Promoting α-MSH release from POMC neurons; stimulating hypothalamic neurons to release CRH	^350,351,353^
	IL4	IL4Rα, IL13Rα, γc	Inhibit adipogenic differentiation of FAPs by insulin; prevent fatty degeneration of muscle; promote clearance of injured muscle fibers	Activating the STAT6 signaling pathway	^366,367^
	IL6	IL6Rα, gp130	Inhibit myofibril synthesis and stimulate muscle breakdown; promote lipolysis and browning; massive liver hypertrophy and APR; splenomegaly; neuroinflammation; cardiac dysfunction and wasting	Activating the STAT signaling pathway; increasing the expression of Atrogin1; inhibiting phosphorylation of mTOR, EIF4EBP1, and RPS6 kinases	^360,362,363,527^
	IL20	IL22R1, IL20R2	Loss of adipose tissue	Enhancing PD-L1 via IFN-α; promoting macrophage infiltration to adipose tissue; increasing expression of ATGL and HSL	^368^
Tumor necrosis factor	TNFα	TNFR	Promote lipolysis; inhibit protein synthesis and promote muscle atrophy; weaken muscle strength	Inhibiting LPL expression; enhancing ATGL activity; activating the NF-κB signaling pathway; promoting ROS generation; activating the UPS; activating the AMPK pathway; inhibiting the PI3K/Akt pathway	^371,372,528^
Growth factors	TGFβ	TGFβR1, TGFβR2	Stimulate profibrotic muscle; inhibit muscle function recovery; promote skeletal myogenesis	Reversing the IGF-1/PI3K/Akt hypertrophy pathway by inhibiting Akt phosphorylation; inducing smad2/3/4 complex; regulating skeletal muscle oxidative metabolism	^376,379,529,530^
	OSM	OSMR, gp130, LIFR	Promote muscle atrophy; stimulate lipolysis and inhibit adipocyte insulin response	Activating the JAK/STAT signaling pathway; inducing amyotrophic-related genes expression; increasing EDA2R expression	^382,531^
	LIF	LIFR, gp130	Elevate lipolysis; induction of muscle atrophy; appetite suppression	Activating JAK/STAT3, PI3K/Akt/mTOR and MAPK signaling pathways; stimulating α-MSH release; increasing leptin expression	^353,387,390^
	GDF15	GFRAL, ALK1, ALK5	Regulate vagal sympathetic nervous system, inhibit appetite; enhance ATGL-dependent sympathetic nerve activation of adipose tissue lipolysis; promote osteoclast differentiation	Activating Smad pathway, PI3K/Akt signaling pathway and NF-κB signaling^387^ pathway; promoting activation of RET oncogene; activating HPA axis; promoting the expression of MuRF1 and MAFbx/atrogin-1	^155,398,532–534^
Interferon	IFNγ	IL28Rα, IL10Rβ	Accelerate protein degradation; suppression of appetite	Activating JAK/STAT signaling pathway; promoting inflammatory response	^1,11,345^
Chemokines	LCN2	MC4R, SLC22A17	Suppress appetite; activation of immune cells; promote lipolysis	Activating cAMP and MC4R signaling in the hypothalamus; inducing the ferroptosis	^66,67,301,535^

*IL1* interleukin 1, *IL1R1* interleukin-1 receptor 1, *IL1R2* interleukin-1 receptor 2, *α-MSH* α-melanocyte-stimulating hormone, *POMC* recombinant proopiomelanocortin*, CRH* corticotropin-releasing hormone*, IL4* interleukin 4, *IL4Rα* interleukin-4 receptor subunit alpha, *FAP* fibro/adipogenic progenitors, *IL13Rα* interleukin 13 receptor subunit alpha, *γc* cytokine receptor common gamma chain, STAT signal transducers and activators of transcription, *IL6* interleukin 6, *IL6Rα* interleukin-6 receptor subunit alpha, *APR* acute phase response, *EIF4EBP1* recombinant eukaryotic translation initiation factor 4E binding protein 1, *RPS6* ribosomal protein S6, *gp130* glucoprotein 130,*IL20R1* interleukin-20 receptor 1, *IL20R2* interleukin-20 receptor 2, *ATGL* antiadipose triglyceride lipase, *HSL* hormone-sensitive lipase, *TNFα* tumor necrosis factor-α, *TNFR* tumor necrosis factor receptor, *LPL* lipoprotein lipase, *NF-κB* nuclear factor kappa-B, *ROS* reactive oxygen species, *UPS* ubiquitin‒proteasome system, *AMPK* reactive oxygen species, TGFβ hormone-sensitive growth factor

#### Interleukin family

##### Interleukin-1

In advanced cancer patients, immune cells secrete large amounts of proinflammatory cytokines such as IL-1 and IL-6, which further exacerbates the progression of cachexia.^346^ Mechanistically, IL-1 triggers the release of α-MSH from POMC neurons^347^ and has an inhibitory effect on NPY neurons,^348^ which affects appetite by regulating MC4R expression in neurons. These phenotypes can be alleviated by a melanocortin receptor antagonist.^349^ IL-1 can also stimulate the release of corticotropin-releasing hormone (CRH) from hypothalamic neurons, promoting the secretion of adrenocorticotropic hormone and cortisol, thereby mediating the catabolic effects observed in cachexia.^350^ Following adrenalectomy, muscle catabolism is alleviated in mice, suggesting that the HPA axis may serve as a central pathway to mediate the impact of IL-1 on muscle degradation.^351^ IL-1 can be further classified into IL-1α and IL-1β on the basis of different coding genes. Compared with IL-1α, IL-1β has more pronounced proinflammatory effects.^352^ IL-1β facilitates the release of various inflammatory factors through central inflammation, resulting in the dysregulation of POMC and AgRP, which subsequently triggers protein hydrolysis and lipolysis.^353^

##### Interleukin-6

IL-6 was initially identified for its roles in B-cell differentiation and the acute phase of the immune response.^354,355^ Indeed, IL-6 is a crucial cytokine that regulates both innate and adaptive immunity and plays significant roles in various physiological processes, including inflammation, metabolism, apoptosis, cell differentiation, bone homeostasis, and angiogenesis.^356,357^ Although IL-6 exerts its anti-inflammatory and regenerative effects by regulating the JAK/STAT pathway, it also participates in proinflammatory responses through trans-signaling.^358^ Despite its dual function in inflammation, increased levels of IL-6 are detected in patients with cancer cachexia, supporting its role in promoting cancer cachexia.^359^ Indeed, short-term activation of IL-6 signaling can stimulate muscle growth. However, owing to its proinflammatory role, prolonged activation can cause muscle atrophy by stimulating muscle degradation and impairing oxidative capacity.^357,360^ Similarly, administration of recombinant IL-6 and IL-6 overexpression results in decreased muscle mass in a cancer cachexia mouse model,^359^ which can be alleviated by injecting IL-6 receptor antibodies.^361^ Not surprisingly, IL-6 can decrease the myofibrillar protein content and increase muscle wasting. Mechanistically, IL-6 inhibits the activity of the mTOR signaling pathway by reducing the phosphorylation of mTOR, ribosomal S6 kinase and eukaryotic translation initiation factor 4E-binding protein 1.^362,363^ In addition, IL-6 and PTHrP can reduce adipose tissue mass by promoting adipose tissue browning.^56,92^ In mouse models of cachexia, treatment with IL-6 receptor antibodies can suppress lipolysis and prevent browning of WAT.^364^ Recent studies have indicated that peripheral circulating IL-6 can enter the brain and activate neurons in the area postrema and its associated neural networks, leading to brain dysfunction and subsequent cachexia.^365^

In addition to IL-6, IL-4 and IL-8 also play regulatory roles in the process of cachexia. IL-4 is a pleiotropic cytokine that can promote the differentiation of fibro-/adipogenic progenitors into fibroblasts, enhancing the phagocytosis of cellular debris by activating the STAT6 signaling pathway. This process facilitates muscle regeneration. However, fibro-/adipogenic progenitors can also differentiate into adipocytes, which may promote muscle growth.^366,367^ Additionally, treatment with the proinflammatory cytokine IL-20, an anti-IL-20, can improve weight loss and prevent a reduction in adipose tissue mass.^368^

#### Tumor necrosis factor

##### Tumor necrosis factor α

TNF-α is a key regulatory factor in cancer cachexia, as its concentration is positively correlated with the degree of weight loss and muscle wasting. TNF-α influences the development of cachexia through multiple mechanisms, including lipid metabolism, protein homeostasis, and neural regulation. On the one hand, TNF-α inhibits the expression of lipoprotein lipase in adipocytes, resulting in reduced lipid uptake.^369^ On the other hand, TNF-α inhibits G0S2 while promoting the activity of ATGL, thereby enhancing ATGL-mediated lipolysis.^370^ Reduced intake and enhanced degradation collectively shift the lipid metabolic balance in a detrimental direction. In vitro studies using myogenic cell cultures revealed that TNF-α suppresses myoblast differentiation and induces protein loss via the NF-κB signaling pathway.^371^ TNF-α can also stimulate oxidative stress by promoting the production of ROS, which can further activate the NF-κB pathway, thereby increasing muscle protein degradation.^372^ Other studies reported that TNF-α stimulates protein degradation in muscle cells by activating the UPS and upregulating the expression of ubiquitin ligases (such as MuRF-1 and Atrogin-1). Moreover, TNF-α inhibits mTOR signaling by activating AMPK and suppressing the PI3K/Akt pathway, which leads to decreased muscle protein synthesis.^373^ Recent studies have suggested that TNF-α suppresses appetite and increases feelings of satiety by influencing the secretion of appetite-regulating neuropeptides in the hypothalamus, including both appetite suppressants and stimulants.^374^

#### Growth factors

##### Transforming growth factor-β

The TGF-β superfamily encompasses a variety of growth factors, including TGF-β itself, growth differentiation factors, bone morphogenetic proteins, activins and inhibins, which play physiological and pathological roles in different tissues. TGF-β primarily impacts skeletal muscle metabolism, exacerbating the occurrence of cachexia by inhibiting muscle protein synthesis and promoting muscle protein degradation. In a mouse model of Marfan syndrome characterized by fibrillin-1 deficiency, the administration of TGF-β antagonists significantly improved muscle regeneration, indicating that TGF-β is critical for maintaining muscle balance.^375^ In the context of muscle injury, the upregulation of TGF-β promotes muscle tissue fibrosis, further impairing muscle function and contributing to muscle wasting.^376^ The binding of TGF-β to its type I (TGF-βR1) and type II (TGF-βR2) receptors results in the formation of a heterotetrameric complex,^377^ which subsequently induces the formation of the Smad2/3/4 complex, facilitating its translocation into the nucleus. Activated TGF-β signaling, in turn, regulates the proliferation and differentiation of skeletal muscle precursor cells and the transcription of genes associated with protein degradation pathways in fibroblasts.^378,379^ Furthermore, recent studies have shown that TGF-β influences lipid metabolism. TGF-β derived from M2 macrophages in aged mice induces DNA damage through the production of reactive ROS, thereby leading to the senescence of adipose progenitor cells and the inhibition of adipogenesis. However, although elevated p16 expression and adipose tissue atrophy are observed in a cancer cachexia model, blocking TGF-β does not affect the number of p16-high adipose progenitor cells.^285^

##### Oncostatin M

Oncostatin M is involved in various physiological functions, including hematopoiesis, liver regeneration, wound healing, inflammation and metabolism. By binding to the glycoprotein gp130, OSM activates the downstream JAK/STAT signaling pathway and stimulates the expression of STAT-dependent genes by recruiting either the LIF receptor β or the OSM receptor β.^380^ Recent studies have established a link between OSM and cancer cachexia. The upregulated expression of OSM target genes, such as OSMR, is detected in the muscles of mice with tumor implantation. The introduction of recombinant OSM protein into myotubes promotes cellular atrophy by inducing the expression of several muscle atrophy-related genes, including Atrogin-1, which is mediated by the JAK/STAT signaling pathway.^381–383^ While IL-6, LIF, and OSM induce muscle atrophy via the JAK/STAT pathway, OSM appears to exert a more pronounced effect on the cellular transcriptome of myotubes, thus resulting in a stronger atrophic response. Recently, Bilgic et al. reported that OSM can increase the levels of EDA2R in muscle tissues, leading to increased skeletal muscle atrophy, suppressed adipocyte differentiation, and increased adipocyte degradation.^382^ Furthermore, depletion of OSMR in muscle fibers protects mice from weight loss and muscle wasting, which, at least in part, is attributed to suppression of the upregulation of *Eda2r* induced by tumors.^382^ In other words, in patients with cachexia, OSM has a significant effect on both muscle and fat metabolism.

##### Leukemia inhibitory factor

LIF plays a significant role in immune system activation and tumorigenesis, functioning as a pleiotropic cytokine. It affects muscle and fat metabolism and is involved in the CNS’s regulation of appetite, particularly in patients with cancer cachexia. LIF primarily binds to the surface heterodimeric glycoprotein complex gp130/LIF receptor, thereby activating downstream signaling pathways, including the JAK/STAT3, PI3K/Akt/mTOR, and MAPK signaling pathways.^384^ LIF can promote lipolysis and induce cachexia. Experimental findings indicate that LIF activates the JAK/STAT signaling pathway, which allows ATGL to hydrolyze triglycerides into free fatty acids while reducing lipoprotein lipase activity, further accelerating lipolysis.^385–387^ Moreover, LIF serves as an inducer of anorexia. Recent research has demonstrated that LIF expressed in POMC neurons located in the arcuate nucleus of the hypothalamus stimulates the release of α-MSH, thereby inducing anorexia.^353^ LIF secreted from tumor cells can interact with various neuropeptides, leading to the onset of anorexia.^388^ LIF also promotes anorexia by increasing the expression of leptin during acute inflammatory responses.^389^ Additionally, patients with muscle wasting show excessive expression of LIF, suggesting a potential link between LIF and muscle wasting, possibly related to the activation of signaling pathways such as the JAK/STAT, ERK1, and MAPK pathways.^390^ Furthermore, treatment with LIF antibodies such as PIAS3 and ruxolitinib effectively alleviated muscle wasting.^390^

##### Growth differentiation factor 15

GDF15 is a stress-induced cytokine and a new member of the TGF-β family that was first discovered in activated macrophages. Research has demonstrated that GDF15 expression is significantly elevated in various cancers, including prostate cancer, colorectal cancer, and breast cancer, and its serum levels in patients are nearly twice as high as those in healthy controls.^141,391^ Like TGF-β, GDF15 can activate the Smad signaling pathway, the PI3K/Akt signaling pathway, and the NF-κB signaling pathway.^392^ Johnen et al. reported that overexpression of GDF15 in tumor-bearing mice significantly reduced food intake, which indirectly caused decreased fat reserves and atrophy in the tibialis and gastrocnemius muscles.^393^ GDF15 can also bind to the specific receptor GFRAL to activate the Ret proto-oncogene, which promotes ERK and Akt phosphorylation, resulting in decreased appetite. It activates the HPA axis to promote the secretion of CRH and glucocorticoids.^155,394^ In cachectic mice, the activation of the GFRAL/RET signaling pathway upregulates the expression of iodothyronine deiodinase 2 and β3-adrenergic receptors (β3-AR), causing fat reduction.^155^ GDF15 is also an important factor for inducing muscle wasting. In vitro experiments have shown that GDF15 can promote the expression of MuRF1 and MAFbx/Atrogin-1, leading to a reduction in muscle fiber diameter.^395^ Lerner et al. confirmed that GDF15 facilitates the reduction of the gastrocnemius and soleus muscles by activating mitogen-activated protein kinase 11.^396^ Furthermore, GDF15 enhances the expression of the lipid-mobilizing factor ZAG through the JPA axis and stimulates lipolysis via β3-AR.^397^ Similarly, another study demonstrated that GDF15 promotes the expression of differentiation and thermogenic genes in brown adipocytes. Additionally, GDF15 promotes osteoclast differentiation and inhibits osteoblast differentiation both in vivo and in vitro, leading to bone metabolism disorders.^398^ These findings support the crucial role of GDF15 in regulating distinct cancer cachexia phenotypes via different mechanisms.

#### Interferon

##### Interferon-γ

IFN-γ, also known as immune interferon, is classified as a type II interferon that is produced primarily by activated Th1 cells, NK cells and CD8^+^ cytotoxic T lymphocytes. IFN-γ exacerbates systemic inflammatory responses and promotes protein breakdown, making it a key factor leading to the progression of cachexia. Moreover, IFN-γ is considered a crucial element in the progression of cachexia. Research has demonstrated that IFN-γ promotes the upregulation of muscle-specific E3 ubiquitin ligases, such as MuRF1 and Atrogin-1, by activating the JAK/STAT signaling pathway, which accelerates protein degradation.^399^ The elevation of IFN-γ is closely associated with the release of proinflammatory signaling molecules (such as TNF-α and IL-6), which collectively enhance systemic inflammatory responses and worsen the symptoms of cachexia.

#### Chemokines

##### Lipocalin 2

LCN2, which is synthesized primarily by the immune system, is secreted into the circulation in various diseases associated with cachexia.^400^ Recent studies have indicated that LCN2 has an appetite-inhibiting effect. When LCN2 is injected intraperitoneally into *Lcn2*-deficient mice, it specifically binds to MC4R in the paraventricular and ventromedial neurons of the hypothalamus and activates an MC4R-dependent anorexigenic pathway.^66^ Similarly, the absence of LCN2 mitigates the loss of lean and fat mass associated with cachexia-induced anorexia, whereas the restoration of *Lcn2* expression in the bone marrow rescues the anorexia feature of cachexia.^67^ Moreover, the increase in food intake due to LCN2 blockade is unrelated to systemic inflammatory status or immune activation.^67^ These findings suggest that LCN2 plays a key role in reducing appetite in cancer cachexia patients.

In summary, various cytokines secreted by different cells in the microenvironment play significant roles in the development of cancer cachexia. These cytokines include classical inflammatory factors such as TNF-α, IL-1, IL-6, and IFN-γ. Upon release into the circulation, these cytokines exert diverse effects on various tissues, such as the brain, muscle, and adipose tissues, leading to appetite suppression, suppression of muscular protein production, and promotion of lipolysis, which ultimately lead to the progression of cachexia. Furthermore, TNF-α and IL-1 can enhance the activation of the IL-6 signaling pathway, further exacerbating the progression of cachexia. Therefore, targeting cachexia-inducing factors may provide new avenues for managing cancer cachexia.

### Regulatory roles of cell signaling pathways

Cytokines constitute pivotal components in the pathogenesis of cachexia, primarily regulating skeletal muscle wasting and fat depletion via receptor-mediated signal transduction cascades. Therefore, various signaling pathways activated by proinflammatory cytokines or inflammation-related ligands are crucial for muscle atrophy through the regulation of muscle protein turnover. Additionally, various signaling pathways exacerbate fat depletion by regulating the activity of lipid metabolism-related enzymes and accelerating the browning of WAT. Thus, signaling pathways serve as the key hubs between cytokines and skeletal muscle wasting, as well as lipid metabolic dysregulation. A deeper investigation into these signaling pathways will enhance our understanding of the underlying pathological mechanisms of related diseases.

#### PI3K/Akt signaling pathway

The PI3K/Akt signaling pathway is involved in numerous physiological and pathological processes, including the regulation of cell proliferation and apoptosis, angiogenic responses, metabolic homeostasis, and the generation of muscle and fat tissue.^401^ This pathway is modulated by multiple signals, and in patients with cachexia, the PI3K/Akt pathway is modulated primarily by the HPA axis. The axis is a crucial component of the neuroendocrine system, integrating signals from the brain to peripheral systems and coordinating the endocrine system, nervous system, and immune system. The HPA axis consists of three main parts: the hypothalamic paraventricular nucleus, the anterior pituitary, and the adrenal cortex^402^ (Fig. 7). The paraventricular nucleus secretes CRH, which can suppress appetite by modulating leptin levels.^403^ The adrenal cortex synthesizes glucocorticoids that participate in various bodily functions, including digestion, reproductive behaviors, and energy storage and expenditure. Research has indicated that glucocorticoids induce muscle atrophy by suppressing the PI3K/Akt signaling pathway, leading to the upregulation of FoxO transcriptional regulators (FoxO3a and FoxO1). This activation increases the transcription of MAFbx and MuRF1, enhancing the proteolytic activity of the UPS. Concurrently, glucocorticoids can increase skeletal muscle resistance to anabolic regulators, such as IGF-1, promoting protein degradation while inhibiting protein synthesis, ultimately causing muscle atrophy.^404^ Importantly, the HPA axis is also modulated by inflammatory cytokines such as IL-1, IL-6, and TNF-α. These cytokines may individually or synergistically stimulate CRH secretion, ultimately leading to increased glucocorticoid release, appetite suppression, and exacerbation of muscle wasting in patients.^405,406^

#### NF-κB signaling pathway

The NF-κB signaling pathway is important for diverse biological activities, including inflammatory responses, immune responses, cell apoptosis, and stress responses. Hyperactivation of the NF-κB pathway has been linked to numerous human diseases, particularly tumorigenesis. Abnormalities in the NF-κB signaling pathway lead to dysregulation of cell proliferation, viability, motility, and invasion, thereby promoting tumor development.^407,408^ In cachexia patients, circulating concentrations of inflammatory factors (such as IL-1 and TNF-α) are significantly elevated. These bioactive molecules activate their respective receptors, recruiting adaptor proteins to activate I-kappa-B kinase, which in turn triggers the nuclear translocation of NF-κB. Among these, TRAFs serve as intracellular adaptors that interact with surface receptors such as TNFR-1, TNFR-2, TLR4, and IL-1R, integrating upstream inflammatory signals and activating the downstream NF-κB pathway.^409,410^ This activation upregulates the expression of MAFbx and MuRF1 (Fig. 7). The activation of NF-κB is considered a critical step in inflammation-mediated skeletal muscle atrophy. In models of systemic inflammation, the inhibition of muscle NF-κB signaling in genetically modified mice alleviated muscle wasting.^411^ This signaling pathway, particularly in response to IL-1 and TNF-α, serves as a significant mechanism for inducing muscle wasting. In vitro studies revealed that C2C12 myoblasts exhibit increased NF-κB pathway activation following TNF-α treatment. Moreover, the use of the NF-κB inhibitor PDTC effectively suppressed the upregulation of MuRF1 mediated by TNF-α intervention.^412^ IL-1, a proinflammatory cytokine with effects similar to those of TNF-α, is also significantly elevated in cachexia patients. Parallel experiments indicate that IL-1 activates NF-κB signaling, resulting in the upregulation of MuRF1.^413^ Additionally, NF-κB reduces the abundance and activity of MyoD and Myf-5 proteins, decreases MyoD mRNA expression, and inhibits Akt-mediated dephosphorylation of FoxO3a, facilitating its nuclear translocation and inducing the transcription of the target genes MAFbx and MuRF-1. This cascade ultimately impairs postnatal myogenesis.^412,414^

#### JAK-STAT signaling pathway

The JAK-STAT signaling pathway is a crucial inflammatory signaling cascade that plays significant roles in regulating cell development, proliferation, metabolism, inflammation, and cancer. Approximately 60 cytokines, including IFNs, colony-stimulating factors, interleukins, and growth factors, are known to activate the JAK/STAT signaling pathway.^415^ Upon cytokine binding to its receptor, JAKs are recruited and phosphorylated. The phosphorylated JAKs then activate STAT proteins, which dimerize and translocate to the nucleus, where they attach to target DNA sequences and modulate gene expression^415^ (Fig. 7). The JAK family consists of four nonreceptor tyrosine kinases, namely, JAK1, JAK2, JAK3, and tyrosine kinase 2 (TYK2), which interact noncovalently with the cytoplasmic domains of cytokine receptors.^415,416^ The STAT family includes STAT1, STAT2, STAT3, STAT4, STAT5A, STAT5B, and STAT6. Once phosphorylated, STAT proteins form dimers that act as transcription factors, altering chromatin accessibility and inducing gene transcription.^417^ In cachexia patients, the regulation of the JAK/STAT pathway by the IL-6 family of receptors (including IL-6, OSM, and LIF) is particularly pronounced. After binding to their receptor complexes, IL-6 family factors activate STAT transcription, promoting the expression of MSTN, MAFbx, MuRF1, and caspase-3 in muscle fibers,^418,419^ which leads to muscle protein catabolism. Studies have shown that IL-6 levels and the STAT3 signaling pathway are markedly increased in the skeletal muscle of cachexia mice induced by C26 cancer cells and that the inhibition of STAT3 alleviated muscle wasting. Similar results were observed through the inhibition of STAT3 signaling in C2C12 cells in vitro.^359^ Similarly, the administration of IL-6 receptor antibodies to C26 cachexia mice mitigated muscle loss.^420^ Hashimoto et al. demonstrated that recombinant IL-6 treatment reduced the diameter of C2C12 myotubes while increasing STAT3 phosphorylation and Atrogin-1 transcription.^421^ Overall, IL-6 phosphorylates STAT3 via the JAK/STAT pathway, leading to the overexpression of MAFbx and subsequent muscle wasting. Additionally, the STAT transcription factor family can be regulated by other kinases, such as mTOR and MAPK, which can phosphorylate STAT3.^422^ STAT transcription factors also interact with FoxO and NF-κB to regulate gene transcription.^423,424^ In addition to the IL-6 family, IFN-γ also utilizes the JAK/STAT signaling pathway to upregulate MuRF1 and MAFbx, accelerating protein degradation.^399^ Recent studies have shown that IL-6 can promote the expression of UCP-1 via the JAK/STAT signaling cascade, thereby facilitating WAT browning, and can also directly enhance ATGL-dependent lipolysis.^425–427^

#### TGF-β/SMAD signaling pathway

The SMAD signaling pathway involves an intracellular signaling mechanism that modulates cell growth, differentiation, migration, and invasion. The SMAD proteins are categorized into three classes: the first class comprises receptor-regulated SMADs (R-Smads), which are downstream signaling molecules of the TGF-β receptor complex and include mainly Smad1, Smad2, Smad3, Smad5, Smad8, and Smad9; the second class comprises comediator SMADs, of which only Smad4 has been identified in mammals; and the third class consists of inhibitory SMADs (I-Smads), which include Smad6 and Smad7. The SMAD signaling pathway is regulated by various ligands, with members of the TGF-β family playing a significant role in modulating SMAD signaling and regulating skeletal muscle generation and metabolism.^428^ In cachexia patients, TGF-β family members (such as MSTN, TGF-β, and GDF15) bind to receptor complexes (TGFβRI, TGFβRII, and ActRIIB), leading to the phosphorylation of Smad2 and Smad3, which then combine with Smad4. This complex stimulates FoxO-dependent transcription (Fig. 7), thereby regulating the expression of genes linked to the proliferation and differentiation of skeletal muscle progenitor cells, as well as protein breakdown in mature muscle fibers.^429–431^ Furthermore, phosphorylated Smad2/3 can inhibit Akt, leading to the activation of FoxO, which results in increased protein catabolism.^432^ Costelli et al. demonstrated that MSTN can induce cachexia in mice via ActRIIB.^433^ Conversely, blocking the ActRIIB receptor can prevent cachexia in mice with C26 tumors, with no significant alterations detected in the levels of IL-6, TNF-α, or IL-1β.^434^ Additionally, TGF-β can activate noncanonical signaling cascades, such as the MAPK pathway, to influence the development of cachexia.

#### MAPK signaling pathway

The MAPK signaling pathway is one of the oldest evolutionary signaling pathways, mediating various intercellular interactions and receiving various extracellular stimuli, including cytokines and environmental changes, to regulate cellular responses. The MAPK signaling cascade consists of three tiers: MAPK, MAPK/ERK kinase (MEK or MKK), and MAPK kinase kinase (MEKK or MKKK). Moreover, the MAPK pathway is subdivided into four distinct branches: ERK1/2, p38 MAPK, c-Jun N-terminal kinase (JNK), and ERK5.^435^ In skeletal muscle cells, the MAPK signaling pathway regulates myocyte growth and stress responses, particularly in cachexia patients, where MAPK activation promotes protein catabolism.^436^ This process is primarily mediated by IL-1 or TNF-α. When TNF-α binds to its receptors TNFR1/2, it activates a TRAF2-mediated JNK-dependent kinase cascade, sequentially activating MKKs, MAPKs, and ultimately p38 MAPK. After nuclear translocation, p38 MAPK upregulates the transcription of MuRF1 and Atrogin-1, leading to increased protein degradation^437^ (Fig. 7). Similarly, IL-1 was shown to upregulate Atrogin-1 expression through the phosphorylation of p38 MAPK.^413^ Additionally, JNK can inhibit muscle formation by phosphorylating MRF4, which suppresses the expression of late-selective myogenic genes, thus adversely affecting myogenesis.^438^ These findings underscore the crucial function of the MAPK signaling pathway in the manifestation of cancer cachexia.

## Clinical trials and therapeutic approaches

Despite considerable attention being given to the fundamental mechanisms of cancer cachexia, the complicated pathogenesis of cancer cachexia across different cancer types remains incompletely understood, leading to a lack of effective treatment options for different cancer types. To date, most interventional studies have concentrated on dietary and appetite-enhancing measures, anabolic stimulation, and anticytokine therapies. In this section, we summarize the results of clinical trials conducted over the past several years (Table 3) and discuss the clinical trials and promising agents used in preclinical models aimed at delineating cancer cachexia across different cancer types.

**Table 3:** Clinical trials of anticachexia treatment

Compound/drug	Target/agent	Study	Phase	Status	Cancer Type	Treatment outcomes	References
Anamorelin	Ghrelin receptor agonist	NCT00267358	II	Completed	BRCA,CRC,LC,PC,RCC and other	Increased LBM	^536^
		NCT00219817	II	Completed			
		NCT01387282 NCT01387269	III	Completed	NSCLC	Increased LBM, but not handgrip, strength	^499^
		NCT01395914	III	Completed	NSCLC	Improved BW and symptom burden	^500^
		JapicCTI-142451	II	Completed	NSCLC	Increased LBM; improved anorexia symptoms and the nutritional state	^502^
		JapicCTI-111415	II	Completed	NSCLC	Increased LBM; improved QoL	^501^
		JapicCTI-163426	-	Completed	GI cancer	Increased LBM and BW	^451^
		Garcia JM et al.	-	Completed	BRCA,CRC,LC,NHL,PC and other	Increased BW and food intake; improved appetite	^537^
		NCT03743064	III	Completed	NSCLC	Increased BW	NA
		NCT03743051	III	Completed	NSCLC	Increased BW	NA
		NCT03637816	II&III	Active, not recruiting	NSCLC	NA	NA
		NCT04844970	II	Recruiting	PDAC	NA	NA
		NCT01505764	II	Terminated	NSCLC,CRC	Terminated due to poor recruitment	NA
Macimorelin	Ghrelin receptor agonist	NCT01614990	II	Completed	GI cancer,LC,NE, Hematologic cancer	Improved BW and QoL	^450^
Ponsegromab	GDF15	NCT05546476	II	Active, not recruiting	NSCLC,PDAC,CRC	Increased BW and overall activity level; reduced cachexia symptoms	^473^
		NCT04299048	Ib	Active, not recruiting	NSCLC,PDAC,CRC	Improved BW, appetite, and physical activity; suppressed serum GDF15 levels	^472^
		NCT04803305	I	Completed	NSCLC,PDAC,CRC,PC,BRCA,OC	NA	NA
AV-380	GDF15	NCT05865535	I	Recruiting	CRC,PDAC	NA	NA
LY2495655	Myostatin	NCT01505530	II	Completed	PDAC	No clinical benefit	^538^
Enobosarm/Gtx-024	Androgen Receptor	NCT00467844	II	Completed	NSCLC, CRC and other	Improved LBM	^494^
		NCT01355497 NCT01355484	III	Completed	NSCLC	NA	^492^
Testosterone	Androgen Receptor	NCT00878995	I	Completed	HNSCC,CC	Improved LBM, QoL and physical activity	^511^
APD209	Androgenmetabolism	NCT00895726	II	Completed	NA	NA	NA
Espindolol/MT-102	ß-adrenergic	NCT01238107	II	Completed	NSCLC,CRC	Reversed weight loss; improved fat free mass; maintained fat mass	^485^
VT-122	ß-adrenergic	NCT00527319	II	Completed	NSCLC	NA	NA
		NCT01265576	II	Unknown	HCC	NA	NA
Xilonix/MABp1	IL-1α	NCT02138422	III	Completed	CRC	A significant reduction in systemic inflammation and thrombocytosis	^457^
ALD518	IL-6	NCT00866970	II	Completed	NSCLC	NA	NA
Selumetinib	MEK	Prado CM et al.	II	Completed	BTC	Promoted muscle gain	^482^
		Bekaii-Saab T	II	Completed	BTC	Gained nonfluid weight	^481^
Binimetinib	MEK	NCT00959127	I	Completed	BTC	Increased nonfluid weight	^483^
Etanercept	TNF-α	NCT00046904	III	Completed	GI cancer,LC and other	Etanercept does not appear to palliate the cancer anorexia/weight loss syndrome	^462^
Infliximab	TNF-α	NCT00040885	III	Completed	NSCLC	Early evidence of the lack of efficacy promoted early trial closure	^539^
		NCT00060502	II	Completed	PDAC	No statistically significant differences in LBM	^478^
Curcumin	NF-кB	NCT04208334	II	Completed	HNSCC	Increased muscle mass	^509^
		TCTR20220521003	IIa	Completed	LC,HNC,GC,CRC,BRCA and other	Slow progression of hand-grip muscle strength loss, and basal metabolic rate; increased the body composition(not statistically significant)	^459^
Ruxolitinib	JAK/STAT	NCT04906746	I	Recruiting	NSCLC	NA	NA
		NCT02072057	II	Terminated	Any type	Terminated due to poor recruitment	NA
Ketorolac	NSAIDs	NCT05336266	I	Active, not recruiting	PDAC	NA	NA
Celecoxib	NSAIDs	Lai V et al.	II	Completed	HNC,GI cancer	Increased BW and BMI; improved QoL	^460^
		Mantovani G et al.	II	Completed	HNC,LC,CRC,OC,GC,BRCA	Increased LBM; improved grip strength, QoL, performance status; decreased of TNF-alpha	^461^
		IRCT201407222027N4	III	Completed	GI cancer	Adding celecoxib to megestrol could not enhance anti-cachexic effects of megestrol	^446^
Lenalidomide	Immunomodulatory agent	NCT01127386	I/II	Completed	Any type	NA	NA
Thalidomide	Immunomodulatory agent	Gordon JN et al.	-	Completed	PDAC	Increased weight and arm muscle mass; improved physical function	^477^
		Davis M et al.	II	Completed	LC,BRCA,HNC,GI cancer, and other	Improved appetite and QoL	^463^
		Yennurajalingam S et al.	-	Completed	HNC,BRCA,MM,GI and Genitourinary cancer	Both the thalidomide and the placebo groups showed significant reduction in cytokines, improvement were not significantly different in two group	^464^
		Wen HS et al.	-	Completed	LC,BRCA,HPB,GI cancer,	A combination regimen of Megestrol acetate and thalidomide is more effective than Megestrol acetate alone	^465^
OHR/AVR118	Immunomodulatory agent	NCT01206335	II	Unkown	PDAC,LC,CRC,GC,HNSCC,PC	Weight stabilization or gain; improvements in anorexia, dyspepsia, strength, and depression	^466^
N-acetylcysteine	Antioxidant	NCT00196885	II	Completed	GI cancer,LC	Increased knee extensor strength and the sum of all strength paramete; decreased plasma TNF-alpha	NA
L-CARnitine	Antioxidant	NCT01330823/ISRCTN83465351	III	Suspended	PDAC	Increased BMI; improved nutritional status (body cell mass, body fat) and QoL; increased overall survival	^479^
Insulatard	Insulin	NCT00329615	IV	Completed	BTC,PDAC,GI cancer, and other	NA	NA
Insulin	Insulin	Lundholm K et al.	-	Completed	GI cancer,HPB	Increased carbohydrate intake; increased whole body fat; improved metabolic efficiency during exercise; decreased serum-free fatty acids,	^468^
Pioglitazone	Insulin sensitizer	NCT05919147	II	Recruiting	NSCLC,GI cancer	NA	NA
Nandrolone	Corticosteroid	NCT03263520	-	Completed	GI cancer,HPB	NA	NA
Melatonin	Pleiotropic hormone	NCT00513357	III	Completed	LC,GI cancer	Oral melatonin 20 mg at night did not improve appetite, weight, or QoL compared with placebo	^540^
Adenosine Triphosphate	ATP	Beijer S et al.	-	Completed	LC,HNC,PDAC,HL,PC,CC,HCC,GI cancer and other	Increased triceps skin fold thicknes; improved survival	^541^
		NCT00014248	I	Completed	Any type	NA	NA
Olanzapine	Antipsychotic, Appetite stimulant	Sandhya L et al.	-	Completed	GC,HPB,LC	Improved appetite; increased weight	^454^
		NCT05243251	III	Completed	Any type	NA	NA
		NCT00489593	I	Completed	Any type	NA	NA
		NCT06517199	III	Recruiting	Any type	NA	NA
		NCT05705492	II	Recruiting	ESCC,GC,HPB,LC	NA	NA
Mirtazapine	Antidepressant, Appetite stimulant	Riechelmann RP et al.	II	Completed	GI cancer, BRCA, LC and other	Increased BW; improved appetite and health-related QoL	^455^
		NCT03283488	II	Completed	PC,LC,HNC,GI cancer and other	Weight gain and appetite improved	^456^
		NCT04748523	-	Completed	NSCLC	Increased energy intake(mainly in fat intake); achieved energy requirements; improved QoL	^490^
		NCT03254173	II&III	Completed	Any type	NA	NA
		NCT05380479	II	Unkown	Any type	NA	NA
		NCT01501396	II	Withdrawn	Any type	NA	NA
Megestrol acetate	Appetite stimulant	NCT00006799	III	Completed	HNC and LC	Maintained BW; improved QoL	^506^
		NCT00439101	-	Completed	AML,ALL,HL,NHL OS,ES (Children)	Increased weight	^542^
		IRCT201407222027N4	III	Completed	GI cancer	Increased BW; improved grip strength, appetite score and QoL	^446^
		NCT00031785	III	Completed	LC	NA	NA
		NCT00004912	II	Completed	Any type	NA	NA
Nanocrystalline megestrol acetate	Appetite stimulant	NCT06793228	II	Not yet recruiting	SCLC	NA	NA
Ghrelin	Appetite stimulant	NCT00933361	I&II	Completed	PDAC,MESO,PC, NSCLC,CCA, GI and Urogenital cancer	No grade 3/4 toxicity or stimulation of tumor growth, Ghrelin is well tolerated and safe	^448^
Cyproheptadine	Appetite stimulant	NCT00066248	II	Completed	Children cancers	Cyproheptadine is a safe and effective way to promote weight gain	^543^
		NCT01132547	III	Terminated	Children cancers	Terminated due to slow accrual	NA
		NCT05856500	-	Not yet recruiting	GI cancer	NA	NA
Pancrelipase	Appetite stimulant	NCT04098237	II	Recruiting	PDAC	NA	NA
Nabilone	Cannabis	NCT02802540	II&III	Unknown status	LC	Increased caloric intake; improved QoL	^491^
Cannabis	Cannabis	NCT02359123	-	Completed	LC,PDAC,PC,GC,MM,HNSCC,Sarcoma	Increased BW without significant side effects	^453^
Cannabidiol	Cannabis	NCT04585841	I	Completed	Any type	NA	NA
PPP011/CAUM	Cannabis	NCT04001010	III	Suspended	Any type	NA	NA
Omega-3 fatty acids	Nutritional supplement	NCT00003077	I&II	Completed	Any type	A majority of patients did not gain weight, but a small but definite subset of patients had weight stabilization or weight gain	^544^
		KA080091	-	Completed	HNSCC	Improved BW and serum albumin and prealbumin levels	^507^
		NCT01596933	II&III	Completed	HNSCC	Failed to protect against weight loss, or improve nutritional parameters	^508^
		Abe K et al.	-	Completed	PDAC, BTC	Increased skeletal muscle mass, NK cell activity, and absorption of omega-3 fatty acids	^545^
		Werner K et al.	-	Completed	PDAC	Weight and appetite stabilization and improved QoL	^251^
Creatine	Nutritional supplement	NCT00081250	III	Completed	LC,GI cancer and other	Creatine, as prescribed in this trial, had no effect on the cancer anorexia/weight loss syndrome	^443^
Remune	Nutritional supplement	NCT04131426	I	Completed	PDAC,BTC,NSCLC,GI cancer	NA	NA
Oligo-Fucoidan	Nutritional supplement	NCT05623852	II	Recruiting	NSCLC,CRC,HNC, PDAC	NA	NA
Kanglaite	Nutritional supplement	NCT03631459	-	Unknown	LC,HCC,PDAC,GC	NA	NA
		NCT02553187	IV	Unknown	NSCLC,CRC,PDAC	NA	NA
IMN1207	Nutritional supplement	NCT01046383	III	Terminated	NSCLC	Terminated due to slow recruitment	NA
Sipjeondaebo-Tang	Kampo medicine	NCT02468141	-	Completed	BRCA,CC,CRC,GC,LC,TC	Sipjeondaebo-tang did not show a significant effect on anorexia	^546^
Fecal Microbiota Transplantation(FMT)	Microbiota	de Clercq NC et al.	II	Completed	ESCC,GC	Allogenic FMT did not improve any of the cachexia outcomes but improved response and survival	^547^
SXRN	SXRN Plasmid DNA Technique	NCT06736275	I	Recruiting	Any type	NA	NA

*LC*, lung cancer; *NSCLC*, non-small cell lung cancer; *SCLC*, small-cell lung cancer; *GI*, gastrointestinal cancer; *ESCC*, esophageal squamous cell carcinoma; *GC*, gastric cancer; *CRC*, colorectal cancer; *HPB*, hepatopancreaticobiliary; *HCC*, hepatocellular cancer; *BTC*, biliary tract cancer; *PDAC*, pancreatic ductal adenocarcinoma; *HNC*, head and neck cancer; *HNSCC*, head and neck squamous cell cancer; *TC*, thyroid cancer; *NHL*, non-Hodgkin lymphoma; *HL*, Hodgkin’s lymphoma; *CLL*, chronic lymphocytic leukemia; *BRCA*, breast cancer; *CC*, cervical cancer; *OC*, ovarian cancer; *NEC*, neuroendocrine cancer; *MESO*, mesothelioma; *MM, melanoma; OS*, osteosarcoma; NSAID, nonsteroidal anti-inflammatory drugs; *LBM*, lean body mass; *QoL*, quality of life; *BW*, body mass index; *NA*, not available

### Gastrointestinal cancers

Gastrointestinal cancer affects many individuals with lesions from the esophagus to the stomach and down to the rectum. Gastrointestinal cancer patients are more susceptible to cancer cachexia, as direct mechanical or digestive issues related to the tumor burden often impair their appetite and oral intake, and the systemic inflammation and metabolic disorders caused by the tumor also contribute to this risk.^439^ Multiple trials have evaluated treatment methods for cachexia in patients with gastrointestinal cancer and have shown encouraging outcomes.

Considering the widespread accessibility and economic cost of dietary supplements, they undoubtedly hold significance in the management of gastrointestinal cancer. For example, fish oil, which is rich in ω-3 fatty acids, EPA, DHA, and other PUFAs, can lower the concentration of proinflammatory mediators.^439,440^ The use of fish oil may benefit patients with gastrointestinal cancers through increased skeletal and lean muscle mass caused by decreased CRP levels.^441^ However, another study indicated that fish oil can lead to decreased global quality of life and increased appetite loss.^442^ Thus, larger trials are needed in gastrointestinal cancer patients to determine the efficacy of fish oil and to determine which composition of fish oil strongly contributes to its efficacy. Creatine, an amino acid derivative, has no effect on cancer anorexia syndrome in patients with gastrointestinal cancers but may augment muscle strength and improve strength in CRC patients.^443^ Some dietary supplements, such as fish oil and guarana, have favorable effects in the treatment of cachexia, whereas others have not. Therefore, more extensive clinical trials are needed to explore the influence of dietary supplements on cancer cachexia.

Appetite stimulants, including steroids, progestational agents, cyproheptadine, and cannabinoids, were first used to treat cachexia.^444^ For example, Megestrol acetate, a synthetic progestin, is commonly employed to stimulate appetite by acting on NPY present in the ventromedial hypothalamus or by decreasing the synthesis and liberation of proinflammatory cytokines.^445^ Consistently, a randomized, double-blind clinical trial in which Megestrol acetate 320 mg/day was used demonstrated significant improvements in appetite, body weight, grip strength, and quality of life in gastrointestinal cancer patients.^446^ Ghrelin is a neuropeptide that is currently undergoing clinical trials for cancer cachexia. Ghrelin was significantly correlated with the BMI loss ratio and Glasgow prognostic score in gastric cancer patients and demonstrated efficacy in treating cancer cachexia.^447^ Another trial revealed that there were no statistically significant differences in nutritional intake or eating-related symptoms between the groups receiving ghrelin and those receiving a placebo.^448^ Anamorelin and macimorelin are ghrelin receptor agonists that can promote ghrelin secretion by activating the ghrelin receptor, thereby increasing appetite, weight, and muscle mass.^449,450^ In addition, clinical trials have shown their ability to treat cachexia in patients with gastrointestinal tumors.^450–452^ Cannabis, which mainly contains Δ9-tetrahydrocannabinol and cannabidiol, has a notable effect on stimulating appetite and enhancing body weight among HIV-positive individuals and cancer patients. Additionally, clinical trials in gastrointestinal cancer patients confirmed this finding.^453^ In a clinical trial, olanzapine, an antipsychotic medication with antagonistic effects on dopamine and serotonin receptors, was found to enhance appetite and result in weight gain in patients with gastric cancer.^454^ Mirtazapine, a widely used tetracyclic antidepressant with serotonergic and noradrenergic action, has potential as a promising treatment option because of its ability to increase body weight and appetite.^455,456^

Chronic inflammation is one of the main hallmarks of cancer cachexia, and targeting inflammatory cytokines may alleviate the symptoms of cancer cachexia.^13^ MABp1, an antibody targeting IL-1α, resulted in a significant reduction in systemic inflammation and thrombocytosis in CRC patients but did not improve cancer cachexia symptoms. Therefore, larger clinical trials should be conducted to evaluate the treatment efficacy of MABp1 in patients with cancer cachexia.^457^ Curcumin, a polyphenolic compound obtained from turmeric, attenuated proteolysis and muscle wasting and suppressed adipose wasting in mouse models bearing MAC16 and C26 tumors.^458^ Nevertheless, it failed to show superiority over placebo in enhancing body composition among patients with gastrointestinal cancers; thus, additional research is warranted.^459^ An increasing amount of evidence has demonstrated that celecoxib, an inhibitor of cyclooxygenase 2 (COX-2), alleviates cachexia in preclinical models and patients with gastrointestinal cancer through the downregulation of the serum levels of IL-6, VEGF, or TNF-α.^446,460,461^ However, etanercept (a TNF-decoy receptor) does not seem to alleviate cancer anorexia syndrome in patients.^462^ As an immunomodulatory and anti-inflammatory agent, thalidomide has been shown to prevent weight loss, improve appetite, and enhance quality of life in patients with some types of cancer, including gastrointestinal cancer, largely because of its ability to reduce the generation of TNF-α, degrade TNF-α mRNA, and suppress NF-κB pathway activation.^463–465^ Similarly, OHR/AVR118, another immunomodulatory agent, significantly improved anorexia, dyspepsia, and strength and achieved weight stabilization or gain in patients with gastric cancer and CRC.^466^ N-acetylcysteine enhances muscular performance and decreases TNF-α levels in elderly individuals.^467^ Furthermore, a clinical trial demonstrated that N-acetylcysteine decreased TNF-α levels in gastrointestinal cancer patients, thereby alleviating cancer cachexia (NCT00196885).

Cancer cachexia is also driven by metabolic alterations, including increased energy expenditure, elevated plasma glucose, and insulin resistance. Insulin is an important hormone that maintains organ vitality and regulates the metabolism of glucose, proteins, and lipids. Decreased insulin sensitivity leads to reduced glucose absorption by organs and leads to atrophy and a reduction in skeletal muscle and adipose tissue.^468^ Insulin treatment increased carbohydrate intake, lowered serum-free fatty acids, and increased body fat, mainly in the trunk and legs, without affecting lean tissue mass. More importantly, it enhances metabolic efficiency during exercise and improves survival in patients without affecting tumor growth.^468^

GDF15, a member of the TGF-β superfamily, modulates food consumption, the energy metabolism rate, and body weight.^469^ Elevated GDF15 concentrations are correlated with weight loss and poor outcomes in patients with gastrointestinal cancer.^470,471^ Ponsegromab is a potent and highly selective humanized monoclonal antibody designed to bind to circulating GDF15, which effectively blocks the interaction between GDF15 and the GFRAL receptor.^472,473^ A phase Ib study with 10 patients, including 4 CRC patients, revealed that ponsegromab led to improvements in weight, appetite, and physical activity by reducing serum GDF15 levels.^472^ Soon after, a larger trial involving 187 cancer patients reported that inhibiting GDF15 with ponsegromab resulted in increased weight gain, improved overall activity levels, and reduced cachexia symptoms.^473^ These findings support the use of ponsegromab as a therapeutic option for cachexia.

Accumulating evidence suggests that elevated levels of MSTN and its analog activin A lead to the progression of atrophy and cachexia.^474^ ActRIIB, a high-affinity activin type 2 receptor, plays a crucial role in mediating signaling pathways activated by a particular group of TGF-β family ligands, including MSTN, activin, and GDF11. Among these ligands, activins are the most potent negative regulators of muscle mass.^474^ Treatment with sActRIIB, an ActRIIB decoy receptor, not only reversed the decrease in skeletal muscle mass and cancer-induced cardiac atrophy but also prolonged the survival of C26 tumor-bearing mice.^434^ Unfortunately, clinical trials involving decoy ActRIIB led to treatment-related bleeding problems in volunteers, causing their discontinuation as a potential treatment for cachexia (NCT01099761).

### Hepatopancreatic biliary cancers

Hepatopancreato-biliary (HPB) cancers are characterized by poor prognosis and surgically challenging management. Older patients diagnosed with HPB malignancies often suffer from malnutrition and cancer cachexia, resulting in accelerated loss of muscle mass and function beyond the typical age-related decline.^475^ In patients with HPB malignancy, the causes of malnutrition and weight loss are diverse and complex; thus, treatment strategies aimed at maintaining weight and muscle indices are necessary to improve postoperative outcomes.

Systemic inflammation resulting from elevated levels of CRP, an increased neutrophil-to-lymphocyte ratio, and heightened levels of proinflammatory cytokines, including TNF-α, IL-1α, and IL6, is correlated with pancreatic cancer cachexia, poor treatment response, and adverse prognoses.^476^ Thalidomide, an inhibitor of TNF-α, has good tolerability and effectiveness in reducing the loss of weight and lean body mass in patients with cachexia owing to advanced PDAC.^477^ However, infliximab, a monoclonal antibody that targets TNF-α, failed to increase lean body mass.^478^ L-carnitine can regulate inflammatory response mechanisms and improve nutritional status (including body mass and body fat), along with parameters related to quality of life, while also increasing overall survival rates in patients with PDAC.^479^ As mentioned previously, insulin and ponsegromab treatment could alleviate the symptoms of cachexia in patients with gastrointestinal tumors, and the same effect was observed in patients with PDAC.^468,473^ Although patients with BTC generally have a poor prognosis, a recent study demonstrated that the MAPK signaling pathway could play a crucial role in these patients.^480,481^ For example, patients treated with selumetinib, a MEK1/2 inhibitor, experienced an average increase in nonfluid weight and enhanced muscle gain, and another MEK1/2 inhibitor, binimetinib, also demonstrated muscle gain in patients with BTC.^481–483^

For liver cancer, one clinical study, VT-122 (NCT01265576), is under investigation to evaluate the therapeutic effects of clinical drugs for cachexia. However, many preclinical drugs have been reported to alleviate cachexia in mouse models bearing AH-130 Yoshida ascites ascites hepatoma, a cachectic rat tumor. In the Yoshida rat model, the atypical β-blocker S-oxprenolol dose-dependently decreased both weight and lean mass.^484^ In a cancer cachexia clinical trial, MT-102 led to great improvements in lean mass and handgrip strength.^485^ Similarly, in the Yoshida rat model, MT-102 prevented the progressive decrease in fat mass, lean mass, and body weight by decreasing overall protein degradation and stimulating protein synthesis.^485,486^ L-carnitine treatment also ameliorated atrophy of both slow- and fast-twitch muscle fibers, reversed muscle structural abnormalities, and reduced oxidative stress, proteolytic activity, and signaling markers, thereby relieving symptoms in Yoshida tumors in mice.^479^ Therefore, more drugs that have proven effective in preclinical models of liver cancer can be expeditiously transitioned into clinical studies to ascertain their effectiveness and safety in combating cachexia in patients with liver cancer.

### Lung cancer

Lung cancer is the leading contributor to cancer death globally, with NSCLC accounting for the majority (85%) of cases.^487^ Patients with advanced cancer have a heightened risk of developing cancer cachexia. However, approximately 20% of individuals diagnosed with early-stage NSCLC exhibit signs of cachexia, even without any changes in caloric intake.^488,489^ In addition, ~40% of patients with metastatic NSCLC develop cancer cachexia, which is associated with shorter survival.^489^ Therefore, early identification and intervention of cancer cachexia should be considered a top priority and a vital component in the management of NSCLC.

Among the drugs discussed earlier, some can also alleviate cachexia symptoms in lung cancer patients. For example, among patients with NSCLC, mirtazapine led to a significant increase in the intake of energy, including protein, carbohydrate, and fat, as well as a reduction in the proportion of patients who experienced sarcopenia.^490^ Nabilone, a cannabinoid, has been demonstrated to be a safe and adequate therapeutic compound for increasing caloric intake and enhancing the quality of life of lung cancer patients diagnosed with anorexia.^491^ As mentioned in gastrointestinal cancer, ponsegromab enhances body mass and increases appetite among cancer patients, including those with NSCLC.^472,473^

Selective androgen receptor modulators (SARMs) have the potential to increase lean body mass and muscle mass without adverse effects. Enobosarm, a nonsteroidal SARM, is currently under clinical development as a therapy for preventing and treating muscle wasting in cancer patients, as demonstrated in the POWER 1 and 2 trials.^492^ Upon activation, it elicits conformational shifts in the androgen receptor, selectively modulating its interaction with coactivator and corepressor proteins present in various tissues, thereby altering the receptor’s capacity to modulate gene expression.^493^ The enobosarm was granted fast-track designation by the U.S. Food and Drug Administration (FDA), and the outcomes of the phase III trials will be instrumental in determining its approval for clinical use in preventing and treating muscle wasting in patients with NSCLC.^492^ Nevertheless, a completed trial involving patients with NSCLC and CRC revealed the effectiveness of the enobosarm in enhancing lean body mass without exhibiting the toxic impacts typically correlated with androgenic and progestational agents.^494^

β2-adrenergic agonists serve as potent muscle growth accelerators, whereas nonselective β receptor blockade can decrease catabolism. Additionally, antagonism of the central 5-HT1a receptor can lead to reduced fatigue and thermogenesis.^485,495^ Espindolol, a novel nonselective β-blocker, has multiple effects on the aforementioned three pharmacological targets.^485^ It markedly reversed weight loss, enhanced fat-free mass, preserved fat mass, and augmented handgrip strength in advanced NSCLC patients and CRC-related cachexia patients in an ACT-ONE trial.^485,496^ VT-122, another β2-adrenergic agonist for NSCLC, is in progress (NCT00527319).

The most extensively studied anticachexia drug in clinical trials for treating lung cancer is anamorelin. It is a novel selective ghrelin receptor agonist that enhances anabolic processes and stimulates appetite by promoting growth hormone secretion.^497^ In a murine xenograft model of lung cancer, anamorelin significantly increased body weight without promoting tumor growth.^498^ Since then, numerous clinical trials have reported the therapeutic efficacy of anamorelin in patients with NSCLC.^499–502^ For example, in phase 3, the international, randomized, double-blind, placebo-controlled trials ROMANA 1 (323 to anamorelin, 161 to placebo) and ROMANA 2 (330 to anamorelin, 165 to placebo), anamorelin was well tolerated and achieved an increase in lean body mass after 12 weeks, without significant effects on handgrip strength.^499^ ROMANA 3 served as a safety extension of ROMANA 1 and 2, with a total of 513 patients enrolled, including 345 patients receiving anamorelin and 168 patients receiving placebo. Throughout the 24-week treatment period, anamorelin was easily tolerated and significantly increased body weight compared with the placebo.^500^ Concurrently, two randomized phase 2 trials comprising Japanese patients with NSCLC demonstrated that anamorelin treatment resulted in improvements in lean body mass, body weight, appetite, and quality of life without any tolerability concerns.^501,502^ On the basis of the aforementioned clinical study conducted in Japanese cancer cachexia patients, anamorelin (Adlumiz), the first drug used to treat cancer cachexia, was approved for the treatment of cancer cachexia in Japan on January 22, 2021.^503^

### Head and neck cancer

Head and neck cancer (HNC), which directly involves the aerodigestive tract, leads to swallowing dysfunction due to local tumor infiltration and treatment toxicity, resulting in varying degrees of malnutrition in ~50–70% of HNC patients.^504^ Among patients with HNC, those with head and neck squamous cell carcinoma (HNSCC) are particularly prone to developing malnutrition. This is because HNSCC not only promotes the development of cachexia due to its direct physiological effects (leading to dysphagia, odynophagia, and restricted dietary intake) but also exacerbates this process through the secretion of inflammatory cytokines and other cachexia-inducing mediators.^505^

Several pharmacological treatments have been employed in the therapeutic approach for cachexia among patients with HNC. A phase III, placebo-controlled, double-blind randomized study revealed that taking 800 mg of Megestrol acetate per day for 12 weeks helped patients with HNC maintain their weight during curative radiotherapy and enhanced their quality of life.^506^ An Ethanwell/Ethanzyme regimen enriched with ω-3 fatty acids, micronutrients, and probiotics was found to increase body weight, as well as serum albumin and prealbumin levels, in patients with HNC.^507^ However, echium oil, a plant source of ω-3 fatty acids, fails to protect against weight loss or improve nutritional parameters.^508^ Studies conducted both in vitro and in vivo have demonstrated that curcuminoids, extracts derived from curcumin, have an inhibitory effect on NF-κB via intracellular phosphorylation.^458^ Notably, patients treated with curcumin exhibited an increase in muscle mass, along with a decrease in handgrip muscle strength and absolute lymphocyte count.^509^ Cachectic patients with HNC receiving celecoxib can gain weight, experience increased BMI, and improve quality of life without adverse events.^460,461^ By binding to muscle-specific androgen receptors, testosterone represents a well-established approach for addressing skeletal muscle loss.^510^ Indeed, compared with placebo, adjunct testosterone was found to increase lean body mass, improve quality of life, and enhance physical activity in patients with HNSCC.^511^ Immunomodulatory agents, such as thalidomide and OHR/AVR118, also have potential in combating cachexia associated with many cancer types, including HNC; however, standalone cohort studies specifically focused on this unique cancer are lacking.^463,464^

### Other types of cancers

Breast cancer is a life-threatening disease for females and the primary cause of death among women. Some studies have shown that breast cancer patients often have increased serum levels of PGE2 and proinflammatory cytokines, including TNF-α, IL-1β, IL-6, and CRP, which exacerbates the progression of cancer cachexia.^512^ In a randomized, double-blind, controlled study, the plasma fatty acid composition of breast cancer patients who received EPA and DHA significantly altered, and stable CD4^+^ T-cell counts and serum hsCRP levels were maintained, indicating a potential beneficial impact on the immune system and reduced inflammatory activity.^513^ Owing to the increase in proinflammatory cytokines among breast cancer patients, anti-inflammatory drugs may be beneficial for these patients.

HT1080 tumor-bearing mice, which serve as a fibrosarcoma mouse model, can secrete high levels of GDF15 into mouse serum, mimicking the condition of cancer cachexia.^155,396,514^ The relevant signaling complex, which comprises GFRAL and RET in brainstem neurons, was shown to mediate the weight loss induced by GDF15 in mice.^515,516^ A preclinical study demonstrated that 3P10, a therapeutic antagonistic monoclonal antibody targeting GFRAL, can mitigate high-level lipid oxidation and prevent cancer cachexia in tumor-bearing mice by inhibiting RET signaling by blocking the GDF15-induced interaction between RET and GFRAL on the cell surface, even when the mice are under calorie-restricted conditions.^155^ Another RET-selective inhibitor, selpercatinib (LOXO-292), notably increased food intake, enhanced skeletal muscle mass and strength, and promoted adipose tissue while simultaneously reducing body weight loss without exerting a significant effect on tumor growth in tumor-bearing animals. Compared with traditional antibodies, nanobodies, considered next-generation antibody-derived tools, were developed and evaluated in different stages of clinical trials for cancer treatment.^517^ GB18-06, a nanobody specifically directed against GDF15, effectively blocks the GDF15-GFRAL signaling pathway in vitro and reduces weight loss (>20%) in HT1080 tumor-bearing mice.^518^ Bringing these drugs into clinical research as soon as possible will greatly benefit patients with cachexia.

Although several clinical studies have investigated genitourinary, hematologic, and urogenital cancers, the small number of patients involved necessitates further clinical and preclinical studies to identify effective pharmacological agents for these specific cancer types.

## Conclusion and future perspectives

In recent decades, investigative efforts in cancer cachexia pathophysiology have focused on skeletal muscle wasting and adipose tissue depletion. Currently, cancer cachexia is recognized as a systemic disease. The progression of cancer cachexia involves other abnormalities in the gut, brain, bone, and heart, highlighting the significant contributions of metabolism, inflammation, and immunology. In this review, we elucidate the intricate crosstalk between various organs/tissues involved in cachexia, which is characterized by metabolic disorders. However, many interaction axes have not been fully characterized, and their roles remain unclear. Research has indicated that altered gut microbiota composition in obese individuals can lead to increased release of gut-derived inflammatory factors into both portal and lymphatic circulations.^519^ Importantly, these factors can induce adipose tissue inflammation by directly infiltrating mesenteric WAT. Therefore, the gut-adipose-liver axis is crucial in obesity and potentially in cancer cachexia. Recently, the liver‒brain axis has also received widespread attention. On the one hand, the regulation of eating behavior within the CNS is influenced by stimuli originating from the liver. On the other hand, neural signals emanating from the CNS influence glucose, lipid, and protein metabolism in the liver.^520^ Additionally, several studies have underscored the importance of the gut‒bone axis^521^ and the bone‒adipose axis in metabolic diseases,^522^ hinting their potential relevance in cachexia. Elucidating the molecular underpinnings of organ crosstalk in cachexia could provide new insights into its pathogenesis and facilitate the development of novel therapeutic strategies.

Various cancer-promoting metabolites circulate in the bloodstream and can elicit inflammatory responses by either directly infiltrating or releasing cachexia-related metabolites and cytokines into secondary sites. Inflammatory responses exacerbate metabolic abnormalities and form a vicious cycle. However, few studies have directly investigated the role of metabolites in cellular responses in cancer cachexia progression. This research gap may represent a pivotal breakthrough in the etiology of cancer cachexia. By delving into how metabolites influence and reshape cellular structure and function, we can gain a deeper understanding of the underlying causes of these cellular alterations in cancer cachexia. This exploration not only offers a novel perspective on the pathophysiology of cachexia but also provides crucial clues and evidence for the development of innovative therapies targeting these cellular alterations.

The role of immune and stromal cells in cachexia is particularly complex. Some cells can mediate both protective and pathogenic responses in cancer cachexia. The inconsistencies observed in research findings within this domain can be attributed to several contributing factors. First, the immune system is highly dynamic and responsive to an extensive range of stimuli, thereby posing significant challenges in isolating the specific contributions of cells in cachexia. Second, the complex interplay between diverse immune cell types and their interactions with other biological systems, such as the nervous and endocrine systems, further enhances the complexity of elucidating the role of certain cells in cancer cachexia. Furthermore, cachexia itself is a multifaceted condition with a plethora of underlying causes, which can further complicate the interpretation of research outcomes. These intricate interactions can influence muscle function and mass in manners that are yet to be fully understood.

Intriguingly, cachexia arising from diverse pathological origins may involve distinct underlying mechanisms. Specifically, macrophage-derived TGF-β1 has been implicated in mediating the senescence of adipocyte progenitors in aged mice, yet it appears to be nonfunctional in the context of cancer cachexia.^285^ Analogously, cytokines that are pertinent to cancer cachexia do not significantly vary with infection-related cachexia.^308^ Furthermore, the functional role of a particular molecule can differ under different pathological scenarios. In response to cold exposure, GDF15 secreted by BAT targets macrophages to suppress the expression of proinflammatory genes.^140^ Conversely, in the context of cancer cachexia, GDF15 is frequently associated with proinflammatory activities. These observations imply that, despite the clinical similarities among these metabolic disorders, their underlying mechanisms cannot be generalized. Investigating the underlying causes of these discrepancies may provide deeper insights into the fundamental mechanisms governing these diseases.

Notably, the incidence rate of cancer cachexia significantly varies among different tumor types, likely because of primary tumor characteristics and specific therapeutic interventions. This variability highlights the importance of the precise elucidation of the distinct molecular pathogenesis characterizing each tumor subtype when developing targeted therapeutic strategies. Our analysis also revealed a significant discrepancy between preclinical and clinical outcomes for certain treatments. While these treatments have shown promise in preclinical models, their efficacy has been markedly reduced in clinical settings. This suggests that current preclinical models may lack the complexity required to fully recapitulate human diseases, limiting the translation of preclinical findings to clinical practice. Therefore, there is an urgent need to develop more sophisticated preclinical models that better mimic human physiopathology. This could involve the use of advanced tissue engineering techniques, such as organoids or patient-derived xenografts, to create more physiologically relevant models. Additionally, incorporating immune components and the TME into these models may further increase their value for clinical translation.

Current treatment strategies for cancer cachexia primarily focus on a multifaceted approach that includes nutritional support, anti-inflammatory therapies, immunotherapy, exercise therapy, and psychological interventions. Studies have revealed that metabolic dysregulation in cancer cachexia patients is closely associated with epigenetic mechanisms such as DNA methylation and histone modifications.^523^ Specific epigenetic changes may lead to an imbalance in muscle synthesis and degradation.^524^ Recent studies have reported that ncRNAs, such as microRNAs, long noncoding RNAs (lncRNAs), and circular RNAs (circRNAs), play a significant role in cancer cachexia.^525,526^ For example, Shi et al. reported that circANAPC7, via the CREB-miR-373-PHLPP2 axis, suppressed tumor growth and muscle wasting in pancreatic cancer.^526^ Therefore, the development of targeted drugs and therapies aimed at epigenetic markers, particularly demethylation agents, holds promise for breakthroughs in the treatment of cancer cachexia.

Overall, the detailed mechanisms of cachexia are complex. Although several causes have been identified and related drugs have been developed, only a very limited number of emerging drugs and other intervention strategies for cancer cachexia exist. Since research aimed at identifying the systematic mechanisms of cancer cachexia is far from saturated, additional studies on this topic are likely to contribute to a more comprehensive understanding of cancer cachexia progression. Overall, a more systematic integration of immunologic and metabolomics approaches, together with the utilization of optimal animal models to mimic the cachectic state, is necessary to uncover novel targets of cancer cachexia.
